# Novel *N*-methylsulfonyl-indole derivatives: biological activity and COX-2/5-LOX inhibitory effect with improved gastro protective profile and reduced cardio vascular risks

**DOI:** 10.1080/14756366.2022.2145283

**Published:** 2022-12-01

**Authors:** John N. Philoppes, Mohamed A. Abdelgawad, Mohammed A. S. Abourehab, Mohamed Sebak, Mostafa A. Darwish, Phoebe F. Lamie

**Affiliations:** a Department of Pharmaceutical Organic Chemistry, Faculty of Pharmacy, Beni-Suef University, Beni-Suef, Egypt; b Department of Pharmaceutical Chemistry, College of Pharmacy, Jouf University, Sakaka, Saudi Arabia; c Department of Pharmaceutics, College of Pharmacy, Umm Al-Qura University, Makkah, Saudi Arabia; d Microbiology and Immunology Department, Faculty of Pharmacy, Beni-Suef University, Beni-Suef, Egypt; e Department of Pharmacology and Toxicology, Faculty of Pharmacy, Nahda University, Beni-Suef, Egypt

**Keywords:** Indole, thiosemicarbazide, thiazolidinone, ADME, dual COX-2/5-LOX, cardiotoxicity

## Abstract

Three novel series of *N*-methylsulfonylindole derivatives **3a&b**, **4a–e,** and **5a–e** were synthesised. Different biological activities of the synthesised compounds were studied. Antimicrobial activity showed that, compounds **4b**, **4e** and **5d** had selective antibacterial activity against the Gram-negative bacteria, *Salmonella enterica and/or E. coli*. The anti-oxidant activity of the synthesised compounds was evaluated by DPPH radical scavenging activity. *In vitro* anti-inflammatory activity was estimated. Compounds **4d**, **4e**, **5b**, and **5d** showed the highest anti-inflammatory activity. The COX-1, COX-2 and 5-LOX inhibitory activities were measured using enzyme immune assay (EIA) kits. Due to the dual COX-2/5-LOX inhibitory activity of compound **5d**, its cardiovascular profile was determined by measuring cardiac biomarkers (LDH, CK-MB, and Tn-I). Besides, the histopathological study of the heart muscle and stomach were examined for the most active COX-2 inhibitors **4e** and **5d**. Finally, a molecular modelling study and pharmacokinetic properties were obtained using different computational methods.

## Graphical abstract

**Figure:**
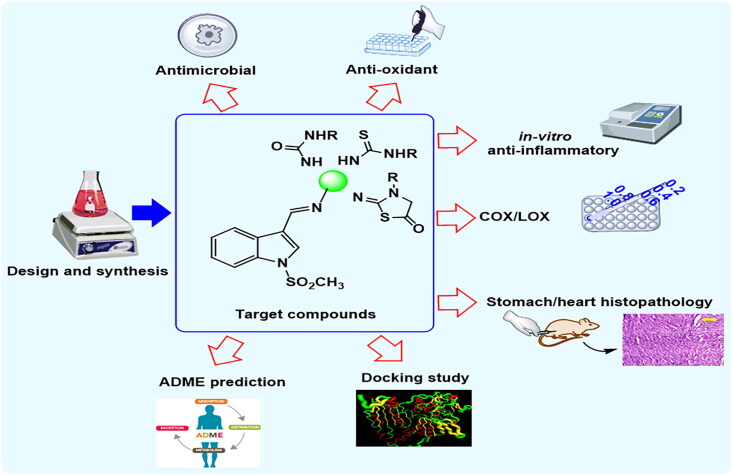

## Introduction

Pathogens, damaged cells and irritants are harmful stimuli that lead the body to make a complex response known as inflammation^1^^,^^2^.

Inflammation is important for the tissue repair process, but in the case of its chronic form, it causes negative effects on the body. Anti-inflammatory agents aim to relieve inflammatory symptoms such as pain, redness, heat and swelling^3^^,^^4^.

**Figure 1.**
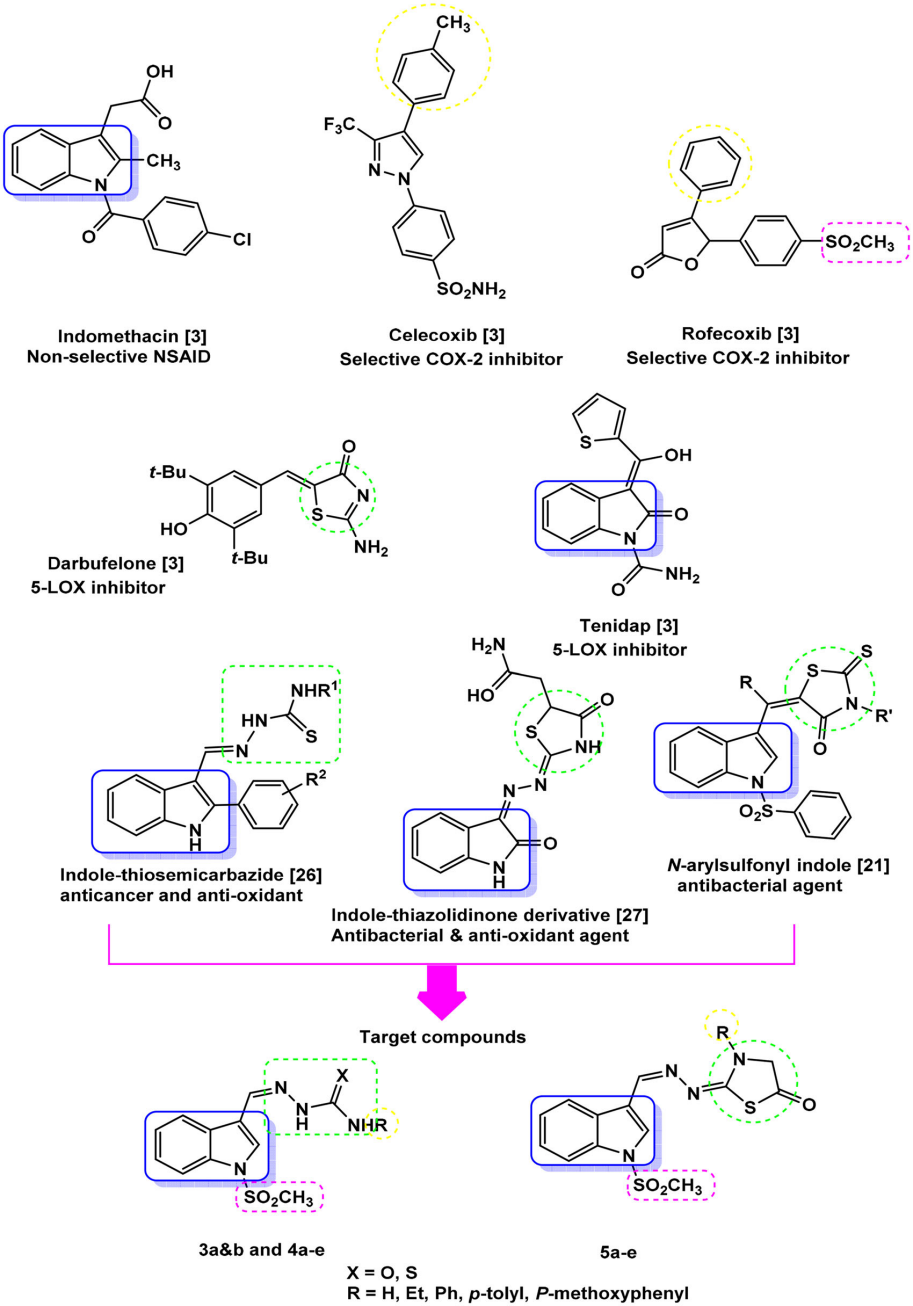
Rational design for the target compounds **3a&b**, **4a–e** and **5a–e**.

Non-steroidal anti-inflammatory drugs (NSAIDs) are the most common method of treatment of inflammatory symptoms. They act through inhibition of biotransformation of arachidonic acid (AA), a membrane bound phospholipid, to prostaglandins (PGs), prostacyclines (PGI2) and thromboxane A2 (TXA2) by the action of cyclooxygenase (COX) enzymes (COX-1, 2, 3)^5–7^. COX-pathway inhibition leads to unwanted side effects such as ulcerogenicity, hepatic and renal toxicity, which have arisen due to COX-1 inhibitors and cardiovascular disorders caused by COX-2 inhibitors. Both NSAIDs that could inhibit both COX-1 and COX-2 enzymes, such as aspirin, phenazone and indomethacin, (Figure 1), as well as selective COX-2 inhibitors, especially like, roficoxib and valdecoxib, (Figure 1) can increase the cardiovascular risks specially in patients with pre-existing cardiovascular disease. This can be caused by the imbalance in PGI2 (potent vasodilator and antithrombotic)/TXA2 (prothrombotic) ratio. Consequently, most drugs have been used in a restricted manner or even withdrawn from the market^8–10^. This leads to a search for new compounds that act in another way to metabolise AA. Liopoxygenase (LOX) enzymes (5-, 8-, 12- and 15-LOX) convert AA to leucotrienes. 5-LOX is the one associated with inflammation, bronchoconstriction, allergy and asthma^11–13^.

As a result, designing new compounds with dual COX-2/5-LOX inhibitory activity might solve the problem by offering new options for developing anti-inflammatory agents with the advantages of selective COX-2 inhibition and at the same time have better cardioprotective profile^2^^,^^14^^,^^15^.

Several biomarkers were reported to be used for the assessment of heart function such as sera containing aspartate aminotransferase (AST), alkaline phosphatase (ALP), lactate dehydrogenase (LDH), troponin (Tn-I) and creatine kinase-MB (CK-MB) in addition to tumour necrosis factor-α (TNF-α), a key player in the inflammatory response and cardiac depression, interleukin-6 (IL-6) and interleukin-1β (IL-1β) as the decrease in their level indicates low risk of cardiovascular toxicity^16–18^.

Besides, tissue including glutathione (GSH) acts as an anti-oxidant in cardiac tissue. It was reported that chronic administration of celecoxib resulted in an increase in lipid peroxidation *via* elevation of oxidative stress markers, which in turn increases oxidative kidney damage, leading to a decrease in GSH level^19^.

Moreover, one of the most important issues related to inflammation is with bacterial infections, which can be considered a primary cause of inflammation area as well as a second complication due to the accumulation of fluid inside the injured area^20^.

Resistance is a serious medical problem that has been observed in the currently used antibacterial agents. It makes the treatment of infectious diseases very difficult. So, the discovery of novel antibacterial agents might be the best way to overcome this episode^21^^,^^22^.

Celecoxib, (Figure 1), the drug of choice as a COX-2 inhibitor, was investigated as an antimicrobial agent as well. It showed potent effects in the reversal of multidrug resistance in MRSA, besides the increased sensitivity of *M. smegmatis* and *S. aureus* to antibiotics^23^.

Another way that affects inflammation is the free radical formation which causes cell damage and inflammation. So, anti-oxidant agents are taken side by side with anti-inflammatory drugs in most cases^24^^,^^25^.

Searching the literature, it was found that indole is a nucleus of choice as a multi-target scaffold for the treatment of inflammation besides its antimicrobial and anti-oxidant activities^26–29^.

This lead scaffold is a universal constituent in pharmacologically active natural products as well as synthetic drugs^30^.

Thus, indole thiosemicarbazide derivatives showed both anticancer and anti-oxidant activity^26^, indole alkaloids obtained from *Alstonia scholaris* and *Topsentia sponge* showed antibacterial activity^4^^,^^31^. Indomethacin, the indole derivative, is considered one of the most promising synthetic drugs as an anti-inflammatory and analgesic agents^8^.

Moreover, it was reported that the indole-thiazolidinone hybrid, (Figure 1), showed equal potency as antimicrobial and antifungal activity (MIC <0.98 µg/ml) if compared to standard drug ciprofloxacin (MIC <3.90 µg/ml)^27^. Additionally, *N*-arylsulfonyl indoles, (Figure 1), exerted remarkable inhibitory activity against Gram-Positive bacteria including multidrug resistance clinical isolates^21^.

On the other hand, carbazones (semi/thiosemi), (Figure 1), are reported to have potential biological activities as antiviral, antimycobacterial, antitrypanosomal, anticonvulsant, antituberculosis, anticancer, anti-inflammatory, antimicrobial, and anti-oxidant^29^^,^^31–35^.

Taking into consideration the various reported biological activities associated with indole, carbazones, and thiazolidinone skeletons, it was worth incorporating these moieties together in a single frame “privileged medicinal scaffolds” to obtain a more potent and biologically active multi-target drug with fewer side effects and a high safety profile.

The design of our newly suggested compounds using the active multi-target drug strategy, (Figure 1), depends on; (i) the main scaffold is indole as indomethacin, the lead compound, (ii) COX-2 pharmacophore, SO_2_Me, from rofecoxib structure, (iii) electron donating substituent on *p*-position of the phenyl ring, to mimic celecoxib, the selective COX-2 inhibitor drug, (iv) introducing carbazone (semi/thiosemi) moieties with reported anti-inflammatory, anti-oxidant and antimicrobial activities on indole C-3, (v) merging thiazolidinone ring with indole scaffold comes from 5-LOX inhibitors drabufelone and tenidap, respectively^3^.

The structure and stereochemical configuration of three series of target compounds **3&b, 4a–e** and **5a–e** were confirmed. *In vitro* anti-inflammatory activities through the determination of TNF-α inhibition in RAW264.7 macrophages, COX-1/2 and 5-LOX inhibitory activities were evaluated. The gastrointestinal and cardiovascular evaluation was determined. The plausible binding interactions inside COX-2 and 5-LOX active sites were explored using a molecular modelling study. Moreover, anti-oxidant and antimicrobial activities were investigated. Finally, ADME prediction and drug-likeness parameters were investigated.

## Experimental

### Chemistry

Melting points were determined using the Griffin apparatus and were uncorrected. Values of IR spectra were measured using Shimadzu IR-435 spectrophotometer with KBr discs and represented in cm^−1^. ^1^H NMR and ^13^C NMR were carried out using the Bruker instrument at 400 MHz for ^1^H NMR and 100 MHz for ^13^C NMR spectrophotometer, (Faculty of Pharmacy, Beni-Suef University, Beni-Suef, Egypt), in DMSO-*d6* (as a solvent), D_2_O using TMS as an internal standard and chemical shifts were recorded in ppm on the δ scale using DMSO-*d6* (2.5) as a solvent. Coupling constant (*J*) values were estimated at Hertz (Hz). Splitting patterns were designated as follows: s, singlet; d, doublet, t, triplet; q, quartette; m, multiplet. Hewlett Packard 5988 spectrometer (Palo Alto, CA) was used to record the electron impact (EI) mass spectra (Microanalytical centre, Cairo University). Microanalysis was performed for C, H, and N on Perkin-Elmer 2400 at the Microanalytical Centre, Cairo University, Egypt and was within ±0.4% of theoretical values. Analytical thin-layer chromatography (TLC), pre-coated plastic sheets, 0.2 mm silica gel with UV indicator (Macherey-Nagel) was employed routinely to follow the course of reactions and to check the purity of products. All other reagents, solvents and compound **1** were purchased from the Aldrich Chemical Company (Milwaukee, WI) and were used without further purification.

### General method for preparation of compounds 3a&b and 4a-e

A mixture of indole derivative **2** (2.23 g, 0.01 mol) and the appropriate semicarbazone or thiosemicarbazone derivative (0.01 mol) in absolute ethanol (20 ml) containing drops of DMF, was heated under reflux for 2–4 h. The obtained solid was filtered, dried, and crystallised from 95% of ethanol to give compounds **3a&b** and **4a-e**.

#### (E)-2{[1-(methylsulfonyl)-1H-indol-3yl]methylene}hydrazinecarboxamide (3a)

Yield 79%; white crystals; mp 238–240^ᵒ^C; IR_._ (cm^−1^): 3404–3122 (NH_2_ and NH), 3122 (NH), 1696 (C = O), 1227, 1165 (SO_2_); ^1^H NMR (DMSO-d_6_) *δ* 3.49 (s, 3H, SO_2_CH_3_), 6.40 (s, 2H, NH_2_, D_2_O exchangeable), 7.37–7.48 (m, 2H, indole H-5, H-6), 7.87 (d, *J* = 8.0 Hz, 1H, indole H-7), 7.97 (s, 1H, indole H-2), 8.11 (s, 1H, N = CH), 8.35 (d, *J* = 7.6 Hz, 1H, indole H-4), 10.22 (s, 1H, NH, D_2_O exchangeable); ^13^C NMR (DMSO-d_6_) *δ* 41.51 (SO_2_CH_3_), 113.25, 117.51, 123.57, 124.39, 125.87, 127.07, 128.87, 135.37, 135.46, 157.10 (C = O); Anal. Calcd for C_11_H_12_N_4_O_3_S (280.06): C, 47.13; H, 4.32; N, 19.99. Found: C, 47.44; H, 4.07; N, 19.82.

#### (E)-2{[1-(methylsulfonyl)-1H-indol-3yl]methylene}-N-phenylhydrazinecarboxamide (3b)

Yield 72%; white crystals; mp 234–236^ᵒ^C; IR_._ (cm^−1^): 3219 and 3123 (2NH), 1673 (C = O), 1238, 1166 (SO_2_); ^1^H NMR (DMSO-d_6_) *δ* 3.52 (s, 3H, SO_2_CH_3_), 6.94–6.98 (m, 3H, phenyl H-3, H-4, H-5), 7.24–7.28 (m, 2H, indole H-5, H-6), 7.31 (d, *J* = 7.6 Hz, 2H, phenyl H-2, H-6), 7.50 (d, *J* = 8.0 Hz, 1H, indole H-7), 8.11 (s, 1H, indole H-2), 8.23 (d, *J* = 8.0 Hz, 1H, indole H-4), 8.70 (s, 1H, N = CH), 9.77 (s, 1H, NH, D_2_O exchangeable), 10.67 (s, 1H, NH, D_2_O exchangeable); ^13^C NMR (DMSO-d_6_) *δ* 41.81 (SO_2_CH_3_), 113.54, 117.18, 118.95, 120.23, 122.33, 123.14, 124.59, 125.94, 127.17, 129.16, 135.42, 136.59, 140.15, 156.50 (C = O); EIMS (*m/z*): 356.98 (M^+^, 35.15%), 192.46 (100.00%); Anal. Calcd for C_17_H_16_N_4_O_3_S (356.40): C, 57.29; H, 4.52; N, 15.72. Found: C, 57.46; H, 4.37; N, 15.68.

#### (E)-2{[1-(methylsulfonyl)-1H-indol-3yl]methylene}-hydrazine-1-carbothioamide (4a)

Yield 82%; white crystals; mp 178–180^ᵒ^C; IR_._ (cm^−1^): 3435 and 3324 (NH_2_), 3171 (NH), 1230, 1166 (SO_2_), 1129 (C = S); ^1^H NMR (DMSO-d_6_) *δ* 3.52 (s, 3H, SO_2_CH_3_), 7.37–7.49 (m, 2H, indole H-5, H-6), 7.65 (s, 1H, NH, D_2_O exchangeable), 7.87 (d, *J* = 8.4 Hz, 1H, indole H-7), 8.22 (s, 1H, indole H-2), 8.32 (s, 2H, NH_2_, D_2_O exchangeable), 8.37 (s, 1H, N = CH), 8.39 (d, *J* = 7.6 Hz, 1H, indole H-4), 11.43 (s, 1H, SH, D_2_O exchangeable); ^13^C NMR (DMSO-d_6_) *δ* 41.63 (SO_2_CH_3_), 113.25, 116.80, 123.72, 124.50, 126.00, 126.78, 130.55, 135.41, 138.71, 177.94 (C = S); Anal. Calcd for C_11_H_12_N_4_O_2_S_2_ (296.37): C, 44.58; H, 4.08; N, 18.90. Found: C, 44.75; H, 3.89; N, 19.13.

#### (E)-N-Ethyl-2{[1-(methylsulfonyl)-1H-indol-3yl]methylene}-hydrazinecarbothioamide (4b)

Yield 73%; white fluffy crystals; mp 240–242^ᵒ^C; IR_._ (cm^−1^): 3278 and 3149 (2NH), 1306, 1207 (SO_2_), 1058 (C = S); ^1^H NMR (DMSO-d_6_) *δ* 1.18 (t, *J* = 7.6 Hz, 3H, CH_2_CH_3_), 3.56 (s, 3H, SO_2_CH_3_), 3.69 (q, *J* = 7.6 Hz, 2H, CH_2_CH_3_), 7.41–7.50 (m, 3H, indole H-5, H-6 and NH, D_2_O exchangeable), 7.88 (d, *J* = 8.0 Hz, 1H, indole H-7), 8.18 (d, *J* = 7.6 Hz, 1H, indole H-4), 8.22 (s, 1H, indole H-2), 8.35 (s, 1H, N = CH), 11.40 (s, 1H, NH, D_2_O exchangeable); ^13^C NMR (DMSO-d_6_) *δ* 15.17 (CH_3_), 38.91 (CH_2_), 41.64 (SO_2_CH_3_), 113.33, 116.81, 123.37, 124.47, 126.00, 126.88, 130.10, 135.39, 138.11, 176.88 (C = S); Anal. Calcd for C_13_H_16_N_4_O_2_S_2_ (324.42): C, 48.13; H, 4.97; N, 17.27. Found: C, 48.44; H, 5.09; N, 17.31.

#### (E)-2{[1-(methylsulfonyl)-1H-indol-3yl]methylene}-N-phenylhydrazinecarbothioamide (4c)

Yield 79%; white crystals; mp 220–222^ᵒ^C; IR_._ (cm^−1^): 3333 and 3122 (2NH), 1270, 1198 (SO_2_), 1129 (C = S); ^1^H NMR (DMSO-d_6_) *δ* 3.51 (s, 3H, SO_2_CH_3_), 7.23–7.40 (m, 5H, indole H-5, H-6 and phenyl H-3, H-4, H-5), 7.44 (d, *J* = 8.4 Hz, 2H, phenyl H-2, H-6), 7.61 (d, *J* = 7.2 Hz, 1H, indole H-7), 8.21 (s, 1H, indole H-2), 8.35 (d, *J* = 7.6 Hz, 1H, indole H-4), 8.45 (s, 1H, N = CH), 9.80 (s, 1H, NH, D_2_O exchangeable), 11.78 (s, 1H, NH, D_2_O exchangeable); ^13^C NMR (DMSO-d_6_) *δ* 41.69 (SO_2_CH_3_), 113.36, 116.65, 123.22, 124.47, 125.78, 126.00, 126.20, 126.98, 128.36, 130.43, 135.38, 138.87, 139.71, 176.18 (C = S); Anal. Calcd for C_17_H_16_N_4_O_2_S_2_ (372.07): C, 54.82; H, 4.33; N, 15.04. Found: C, 54.56; H, 4.19; N, 14.89.

#### (E)-2{[1-(methylsulfonyl)-1H-indol-3yl]methylene}-N-(p-tolyl)hydrazinecarbothioamide (4d)

Yield 81%; white crystals; mp 237–239^ᵒ^C; IR_._ (cm^−1^): 3341 and 3129 (2NH), 1200, 1163 (SO_2_), 1127 (C = S); ^1^H NMR (DMSO-d_6_) *δ* 3.33 (s, 3H, CH_3_), 3.54 (s, 3H, SO_2_CH_3_), 7.19 (d, *J* = 8.4 Hz, 2H, *p*-tolyl H-3, H-5), 7.39–7.47 (m, 4H, indole H-5, H-6 and *p*-tolyl H-2, H-6), 7.89 (d, *J* = 8.0 Hz, 1H, indole H-7), 8.20 (s, 1H, indole H-2), 8.34 (d, *J* = 7.6 Hz, 1H, indole H-4), 8.43 (s, 1H, N = CH), 9.75 (s, 1H, NH, D_2_O exchangeable), 11.75 (s, 1H, NH, D_2_O exchangeable); ^13^C NMR (DMSO-d_6_) *δ* 21.07 (CH_3_), 41.68 (SO_2_CH_3_), 113.36, 116.67, 123.18, 124.46, 126.00, 126.24, 126.99, 129.10, 130.31, 135.02, 135.37, 137.12, 138.69, 176.26 (C = S); EIMS (*m/z*): 386.60 (M^+^, 42.55%), 261.41 (100.00%); Anal. Calcd for C_18_H_18_N_4_O_2_S_2_ (386.09): C, 55.94; H, 4.69; N, 14.50. Found: C, 56.16; H, 4.78; N, 14.23.

#### (E)-N-(4-Methoxyphenyl)-2{[1-(methylsulfonyl)-1H-indol 3yl]methylene}hydrazinecarbothioamide (4e)

Yield 80%; white crystals; mp 214–216^ᵒ^C; IR_._ (cm^−1^): 3431 (broad, 2NH), 1248, 1167 (SO_2_), 1127 (C = S); ^1^H NMR (DMSO-d_6_) *δ* 3.54 (s, 3H, SO_2_CH_3_), 3.78 (s, 3H, OCH_3_), 6.95 (d, *J* = 8.8 Hz, 2H, *p-*methoxyphenyl H-3, H-5), 7.39–7.42 (m, 4H, indole H-5, H-6 and *p*-methoxyphenyl H-2, H-6), 7.44 (d, *J* = 8.0 Hz, 1H, indole H-7), 8.89 (s, 1H, indole H-2), 8.36 (d, *J* = 7.6 Hz, 1H, indole H-4), 8.43 (s, 1H, N = CH), 9.69 (s, 1H, NH, D_2_O exchangeable), 11.74 (s, 1H, NH, D_2_O exchangeable); ^13^C NMR (DMSO-d_6_) *δ* 41.68 (SO_2_CH_3_), 55.70 (OCH_3_), 113.34, 113.82, 116.70, 123.26, 124.43, 125.98, 127.00, 128.10, 130.25, 132.61, 135.37, 138.58, 157.50, 176.61 (C = S); Anal. Calcd for C_18_H_18_N_4_O_3_S_2_ (402.08): C, 53.71; H, 4.51; N, 13.92. Found: C, 53.98; H, 4.65; N, 13.73.

### General method for preparation of compounds 5a–e

A mixture of the appropriate indole derivative **4a–e** (0.0025 mol), ethyl chloroacetate (0.30 g, 0.0025 mol), and sodium acetate (0.82 g, 0.01 mol) in absolute ethanol (20 ml) containing drops of DMF, was heated under reflux for 4–6 h. The obtained solid was filtered, dried, and crystallised from 95% of ethanol to give compounds **5a–e**.

#### (E)-2-(E)-{[1-(methylsulfonyl)-1H-indol-3yl)methylene]hydrazono}thiazolidin-5-one (5a)

Yield 72%; white crystals; mp >300^ᵒ^C; IR_._ (cm^−1^): 3429 (NH), 1712 (C = O), 1255, 1172 (SO_2_); ^1^H NMR (DMSO-d_6_) *δ* 3.55 (s, 3H, SO_2_CH_3_), 3.93 (s, 2H, CH_2_), 7.42–7.51 (m, 3H, indole H-5, H-6 and NH, D_2_O exchangeable), 7.90 (d, *J* = 8.0 Hz, 1H, indole H-7), 8.15 (s, 1H, indole H-2), 8.36 (d, *J* = 8.4 Hz, 1H, indole H-4), 8.63 (s, 1H, N = CH); ^13^C NMR (DMSO-d_6_) *δ* 33.53 (CH_2_), 41.71 (SO_2_CH_3_), 113.50, 117.32, 123.50, 124.59, 126.10, 127.25, 131.77, 135.51, 151.43, 164.07, 172.52 (C = O); Anal. Calcd for C_13_H_12_N_4_O_3_S_2_ (336.04): C, 46.42; H, 3.60; N, 16.66. Found: C, 46.28; H, 3.46; N, 16.37.

#### (E)-3-Ethyl-2-(E)-{[1-(methylsulfonyl)-1H-indol-3yl)methylene]hydrazono}thiazolidin-5-one (5b)

Yield 68%; white crystals; mp 230–232^ᵒ^C; IR_._ (cm^−1^): 1715 (C = O), 1239, 1166 (SO_2_); ^1^H NMR (DMSO-d_6_) *δ* 1.19 (t, *J* = 7.2 Hz, 3H, CH_2_CH_3_), 3.59 (s, 3H, SO_2_CH_3_), 3.78 (q, *J* = 7.2 Hz, 2H, CH_2_CH_3_), 4.01 (s, 2H, CH_2_), 7.42–7.52 (m, 2H, indole H-5, H-6), 7.90 (d, *J* = 7.2 Hz, 1H, indole H-7), 8.17 (s, 1H, indole H-2), 8.38 (d, *J* = 7.6 Hz, 1H, indole H-4), 8.69 (s, 1H, N = CH); ^13^C NMR (DMSO-d_6_) *δ* 12.85 (CH_2_CH_3_), 32.66 (CH_2_), 38.26 (CH_2_CH_3)_, 41.74 (SO_2_CH_3_), 113.53, 117.21, 123.46, 124.64, 126.13, 127.22, 131.99, 135.52, 152.55, 164.07, 172.52 (C = O); EIMS (*m/z*): 364.65 (M^+^, 18.87%), 173.17 (100.00%); Anal. Calcd for C_15_H_16_N_4_O_3_S_2_ (364.07): C, 49.43; H, 4.43; N, 15.37. Found: C, 49.22; H, 4.66; N, 15.09.

#### (E)-2-(E)-{[1-(methylsulfonyl)-1H-indol-3yl)methylene]hydrazono}-3-phenylthiazolidin-5-one (5c)

Yield 79%; white crystals; mp 260–262^ᵒ^C; IR_._ (cm^−1^): 1620 (C = O), 1242, 1165 (SO_2_); ^1^H NMR (DMSO-d_6_) *δ* 3.56 (s, 3H, SO_2_CH_3_), 4.12 (s, 2H, CH_2_), 7.41–7.56 (m, 7H, indole H-5, H-6 and phenyl H-2, H-3, H-4, H-5, H-6), 7.90 (d, *J* = 8.0 Hz, 1H, indole H-7), 8.06 (s, 1H, indole H-2), 8.42 (d, *J* = 8.4 Hz, 1H, indole H-4), 8.53 (s, 1H, N = CH); ^13^C NMR (DMSO-d_6_) *δ* 32.83 (CH_2_), 41.75 (SO_2_CH_3_), 111.94, 113.34, 123.42, 126.13, 127.18, 128.58, 128.75, 129.13, 129.60, 131.96, 134.50, 135.50, 141.58, 152.45, 172.51 (C = O); Anal. Calcd for C_19_H_16_N_4_O_3_S_2_ (412.07): C, 55.32; H, 3.91; N, 13.58. Found: C, 55.53; H, 4.17; N, 13.87.

#### (E)-2-(E)-{[1-(methylsulfonyl)-1H-indol-3yl)methylene]hydrazono}-3-(p-tolyl)phenylhiazolidin-5-one (5d)

Yield 76%; white crystals; mp 271–273^ᵒ^C; IR_._ (cm^−1^): 1621 (C = O), 1241, 1164 (SO_2_); ^1^H NMR (DMSO-d_6_) *δ* 2.39 (s, 3H, CH_3_), 3.55 (s, 3H, SO_2_CH_3_), 4.12 (s, 2H, CH_2_), 7.28 (d, *J* = 8.4 Hz, 2H, *p-*tolyl H-3, H-5), 7.32 (d, *J* = 8.4 Hz, 2H, *p*-tolyl H-2, H-6), 7.45–7.50 (m, 2H, indole H-5, H-6), 7.89 (d, *J* = 8.0 Hz, 1H, indole H-7), 8.09 (s, 1H, indole H-2), 8.39 (d, *J* = 8.0 Hz, 1H, indole H-4), 8.51 (s, 1H, N = CH); ^13^C NMR (DMSO-d_6_) *δ* 21.23 (CH_3_), 32.59 (CH_2_), 42.03 (SO_2_CH_3_), 113.38, 117.08, 123.45, 126.13, 127.12, 128.49, 130.16, 132.00, 132.83, 135.33, 138.74, 149.81, 152.69, 165.40, 172.45 (C = O); Anal. Calcd for C_20_H_18_N_4_O_3_S_2_ (426.08): C, 56.32; H, 4.25; N, 13.14. Found: C, 56.18; H, 4.36; N, 12.89.

#### (E)-3–(4-Methoxyphenyl)-2-(E)-{[1-(methylsulfonyl)-1H-indol-3yl)methylene]hydrazono}-hiazolidin-5-one (5e)

Yield 74%; white crystals; mp 273–275^ᵒ^C; IR_._ (cm^−1^): 1620 (C = O), *1248, 1166* (SO_2_); ^1^H NMR (DMSO-d_6_) *δ* 3.56 (s, 3H, SO_2_CH_3_), 3.83 (s, 3H, OCH_3_), 4.12 (s, 2H, CH_2_), 7.33 (d, *J* = 8.8 Hz, 2H, *p-*methoxyphenyl H-3, H-5), 7.44 (d, *J* = 8.8 Hz, 2H, *p*-methoxyphenyl H-2, H-6), 7.47–7.52 (m, 2H, indole H-5, H-6), 7.90 (d, *J* = 7.6 Hz, 1H, indole H-7), 8.10 (s, 1H, indole H-2), 8.41 (d, *J* = 7.2 Hz, 1H, indole H-4), 8.53 (s, 1H, N = CH); ^13^C NMR (DMSO-d_6_) *δ* 32.71 (CH_2_), 41.74 (SO_2_CH_3_), 55.89 (OCH_3_), 113.53, 114.80, 117.17, 123.47, 124.65, 126.12, 127.20, 128.12, 129.84, 131.95, 135.49, 152.64, 159.66, 165.55, 172.69 (C = O); Anal. Calcd for C_20_H_18_N_4_O_4_S_2_ (442.08): C, 54.28; H, 4.10; N, 12.66. Found: C, 54.49; H, 4.37; N, 12.46.

## Biological evaluation

### Antimicrobial activity screening

All compounds were screened for their antimicrobial activity at the Microbiology and Immunology Department, Faculty of Pharmacy, Beni-Suef University. The antimicrobial activity screening was carried out using the disc diffusion method, as described earlier^36–38^, with some adjustments.

The compounds were evaluated for their bioactivity against the Gram-positive bacteria, *Staphylococcus aureus* (ATCC 43300), *Listeria monocytogenes* (ATCC 7644) and *Enterococcus faecalis* V853, as well as the Gram-negative bacteria, *Escherichia coli* (E. coli) (ATCC 25922), *Pseudomonas aeruginosa* (ATCC 27853) and *Salmonella enterica* (ATCC 14028) in addition to the yeast, *Candida albicans* (ATCC 60193).

For each indicator microbe, a solution of half McFarland turbidity was prepared in sterile saline, followed by surface streaking of the strains on Mueller-Hinton agar with a sterile cotton swab. Then, sterile filter paper discs (5 mm diameter) preloaded with 100 µg of each compound were applied to the surface of the pre-inoculated plates. Then, the plates were chilled at 4 °C for 100 min before being incubated overnight at the proper temperature for microbial growth. Finally, the diameter of the inhibition zones around each disc was measured to evaluate the antimicrobial activity of the compounds. Ciprofloxacin was used as a standard antibacterial agent at a concentration of 20 µg/disc. Furthermore, DMSO-loaded discs were used as a negative control.

### Anti-oxidant activity

Preparation of samples was occurred at the following concentrations: Samples **3a**, **4a**, **5b**, **5d** and **5e** at final concentrations of 0.5 mg/ml in methanol: DMSO 9:1 *v/v*. Samples **4b** and **4d** at final concentrations 0.025 mg/ml in methanol: DMSO 9:1 *v/v*, sample **5a** at final concentrations 0.25 mg/ml in methanol: DMSO 9:1 *v/v*, Sample **4c**, **5c** and **4e** at final concentrations 0.05 mg/ml in methanol: DMSO 9:1 *v/v*, sample **3b** at final concentration 0.75 mg/ml methanol: DMSO 9:1 *v/v*. A stock solution of 100 µM concentration of Trolox, a standard used in this experiment, was prepared in methanol from which seven concentrations were prepared, including *50, 40, 30, 20, 15, 10* and 5 µM.

The DPPH (2,2-diphenyl-1-picryl-hydrazyl-hydrate) free radical assay was carried out according to the method of Boly et al. 2016^39^. Briefly, freshly prepared DPPH reagent (100 µl, 0.1% in methanol) was added to 100 µl of the sample on 96 well plates (*n* = 6). The reaction was incubated at room temp for 30 min in the dark. After completion of the incubation time, the reduction in DPPH colour intensity was measured at 540 nm. Data obtained was represented as means ± SD, according to the following equation, percentage inhibition = [(Average absorbance of blank – average absorbance of the test)/(Average absorbance of blank)] *100. The results were recorded using a microplate reader, FluoStar Omega.

Microsoft Excel^®^ was used to analyse the obtained results. The IC_50_ values were calculated using Graph pad Prism 6^®^ by converting the concentrations to their logarithmic value and selecting the non-linear inhibitor regression equation (log (inhibitor) vs. normalised response – variable slope equation). The methods used and the final test report (R-SO7320) were obtained from Nawah Scientific.

## Anti-inflammatory activity

### Measurements of TNF-α in RAW264.7 macrophage cells

#### Reagents and chemicals

Both Lipopolysaccharides (LPS) and hispidin were purchased from Sigma (Sigma-Aldrich, St. Louis, MO, USA)^40^. The foetal bovine serum (FBS) and Dulbecco’s modified Eagle’s medium (DMEM) were purchased from Hyclone (General Electric Healthcare Life Sciences, Mississauga, Canada), penicillin streptomycin (P/S) was purchased from Solarbio (Solarbio life sciences, Beijing, P. R. China). Results of all *in vitro* anti-inflammatory screening were obtained from the confirmatory diagnostic unit, VACSERA, Egypt.

#### Cell culture

DMEM was used to cultivate the macrophage cells RAW264.7 (Shanghai BOGO Industrial Co., Ltd., Shanghai, China), and supplemented with 10% FBS and 1% P/S (100 U/ml and 100 mg/ml, respectively). The cells were maintained in DMEM at 37 °C and 5% CO_2_. They were treated with various concentrations of hispidin, then with 1 μg/ml LPS for the indicated time.

#### Cell viability assay

The RAW264.7 macrophage cells were seeded into 96 well plates at a concentration of 4 × 10^3^ cells per well, with different concentrations (100 to 0.4 μg/ml) of hispidin treated 24 h. In the next step, to each well, a solution of 5 mg/ml 3–(4,5-dimethylthiazol-2-yl)-2,5-diphenyltetrazolium bromide (MTT; Sigma-Aldrich,) was added and incubated (37 °C, 5% CO_2_) for 4 h. After that, the supernatant was removed and DMSO was added to dissolve formazan. Finally, the absorbance was measured at 490 nm using a UV MAX and kinetic microplate reader (Molecular Devices, LLC).

#### Preparation of reverse transcription quantitative PCR (RT-qPCR) -ready cell lysates

PBS (1 ml/well) was used to wash the cells in 24-well plates. Then, cell monolayers were exposed to 200 ml/well of Bio-Rad iScript (Bio-Rad SPR; 170–8898) to prepare cell lysates (cell-Lysis, CL). The final formulation of CL buffer consisted of 10 mM Tris-HCl, pH 7.4, 0.25% Igepal CA-630, and 150 mM NaCl. CL Buffer was freshly prepared from the following stock solutions: 1 M Tris-HCl (T2194; Sigma), 10% Igepal CA-630 (I8896; Sigma); and 5 M NaCl (351–036-100; Quality Biological, Inc.). For the experiments, CL Buffer included MgCl_2_ (M1028; Sigma) or RNasin Plus RNase Inhibitor (N2615; Promega). Before use, both Bio-Rad SPR and CL buffer were equilibrated to room temperature. Cells were exposed for 2 min to Bio-Rad SPR and 5 min to CL buffer. Carefully, the resulting lysates were collected and either analysed immediately or stored frozen (220 or 280uC).

#### Reverse transcription quantitative PCR (RT-qPCR) gene expression

RT-qPCR analysis was performed in one-step SYBR Green RT-qPCR. Each reaction contained: 1 ml of cell lysate, iScript One-Step SYBR Green RT-PCR Supermix (170–8893; Bio-Rad), 600 nM of each primer, and nuclease-free water to 10 ml. A CFX96 real-time PCR instrument (Bio-Rad) was used under the following protocol: 50uC for 10 min, 95uC for 5 min, 95uC for 10 s/61uC for 15 s/72uC for 30 s.

After the 72 °C extension step, data collection occurred. Total RNA purified from cells was used as an RT-qPCR quantification standard.

#### In vitro cyclooxygenase (COX-1 and COX-2)

Enzyme immune assay (EIA) kits (Catalog no. K548-100, Cayman Chemical, Ann Arbour, MI) were used to test the ability of the prepared compounds to specifically inhibit ovine COX-1 and COX-2 (IC_50_ value, µM), according to reported methods^41–43^. The COX-2 S.I. values were calculated from the ratio (COX-1 IC_50_)/(COX-2 IC_50_) and compared with that of celecoxib, as a selective COX-2 inhibitor.

#### In vitro lipoxygenase (5-LOX) inhibition assay

The ability of the test compounds to inhibit the 5-LOX enzyme (IC_50_ value, μM) was detected using the Cayman Human Lipoxygenase Inhibitor Screening Assay (EIA) kit (catalog no. K980-100, Cayman Chemical). The IC_50_ values of test compounds were measured in µM according to the manufacturer’s instructions and reported method^43^.

## Evaluation of ulcerogenic effect

### Ulcerogenic liability

Chemicals, kits, celecoxib, and indomethacin were purchased from Sigma-Aldrich (St. Louis, MO, USA). CK-MB, Troponin-I, and LDH were purchased from Thermo Fisher Scientific (USA).

#### Animal and ethics statement

In this study, adult male Wister albino rats weighing 160–180 g were used. Before any experimental procedure, rats were given 14 days to acclimate. These rats were maintained in a controlled setting with food and water available. All experiments were conducted in accordance with laboratory animal care guidelines. All experimental procedures were carried out in accordance with the regulations of the Committee of Ethics for Scientific Research on Living Organisms, Faculty of Pharmacy, Nahda University, Beni-Suef (NUB). The approval number was (NUB-019–025).

### Ulcerogenic liability

Ulcerogenic susceptibility was assessed for the most selected candidates (**5b** and **4e**) and compared to indomethacin using celecoxib as a standard. Before drug administration, rats were fasted for 18 h and then separated into four groups. The control group received a suspension of the vehicle (10% DMSO in saline was given P.O (100 mg/kg) once daily). Other groups were given candidates, celecoxib, and indomethacin, in accordance with the described methodology^44^. Rats were given the required dose for three consecutive days. Following the last dose, the stomachs of each rat were dissected and opened via the greater curvature and rinsed with saline. For investigation, the dissected stomach was stretched with pins on a cork board. Through illuminated magnifying lenses, the stomach mucosa was examined for the existence of ulcers. Cho and Ogle’s method^45^^,^^46^ was used to calculate the ulcer index.

### Histopathological examination of stomach

Histopathological tests were performed to evaluate the effects of chemicals (**5b** and **4e**) on the stomach on those of celecoxib and indomethacin, which served as a positive control. In brief, the stomachs of each animal group’s rat were fixed in 10% neutral buffered formalin for 48 h. The sections were then dehydrated, cleared, fixed in paraffin, sectioned (5 m), and stained with H&E stain.

### Cardiovascular evaluation

Eighteen rats were divided into three groups at random (6 animals in each group). The first group was given a vehicle and served as normal control. For two weeks, a suspension of the studied compound **5b** and celecoxib in a 10% DMSO in saline was given P.O (100 mg/kg) once daily. Ketamine (100 mg/kg) was used to anaesthetise the animals on the 15th day. Blood samples were obtained from the retro orbital, allowed to clot, centrifuged at 1000 g for 15 min, and utilised to examine heart function biomarkers (LDH, CK-MB, and troponin). The abdomen of each rat was opened, and the heart was extracted and washed in ice-cold physiological saline before being harnessed for histological investigation.

### Assessment of the cardiac function biomarkers

Troponin, LDH, and CK-MB activities were measured in rats’ sera by spectrophotometer using commercial kits in accordance with manufacturers’ instructions to assess the effect of compound **5d** on cardiac function biomarkers in rat serum and then compared to celecoxib^47–49^.

### Histopathological studies of heart

A histopathological examination was conducted to compare the effect of compound **5b** on the heart to that of celecoxib. In brief, the hearts of rats given compound **5b** and celecoxib, as a control, were fixed in 10% neutral buffered formalin for 48 h. The sections were then dehydrated, cleared, fixed in paraffin, sectioned (5 m), and stained with H&E stain^46^^,^^50^.

### Molecular docking study

Molecular Operating Environment (MOE, 2014.0901) software was used in this study for docking analysis. The crystal structure of both COX-2 bound to its ligand celecoxib and 5-LOX with its ligand arachidonic acid were downloaded from the protein data bank (PDB: 3LN1, https://www.rcsb.org/structure/3LN1 and PDB: 3V99, https://www.rcsb.org/structure/3V99, respectively), with the resolution of 2.40 Ǻ for COX-2 isozyme and 2.25 Ǻ for 5-LOX enzyme. For two enzymes, the crystallised ligands were docked and the binding energy scores, amino acid interactions and relative mean square deviation (rmsd) were calculated. The well fitted and the least energetic poses were selected. The same docking protocol was operated for the synthesised compounds inside both COX-2 and 5-LOX active sites. For each docked compound, the most superposed conformer with the ligand with binding interactions resembling that of the ligand was chosen. The energy binding scores (Kcal/mol) and bond length values (Ǻ) were recorded. 2D and 3D pictures of the most predicted active compounds were taken.

## Computational analysis

### Molecular properties and drug-likeness

Molinspiration (2018.02 version)^51^ was used to calculate molecular properties such as molecular weight (MW), the number of hydrogen-bond acceptors (HBA), number of hydrogen-bond donors (HBD), partition coefficient (MlogP), number of rotable bonds (nrotb), topological polar surface area (TPSA), and Violation of Lipinski’s rule of five (n-violation). Drug-likeness scores were also determined for all target compounds **3a&b**, **4a–e** and **5a–e**.

### Bioactivity prediction

Bioactivity properties of the target compounds **3a&b**, **4a–e** and **5a–e** were checked. The obtained results of a G-protein coupled receptor (GPCR) ligand, ion channel modulator, a kinase inhibitor, nuclear receptor ligand, protease inhibitor, and enzyme inhibitor using molinspiration^51^ were also recorded.

### In silico ADME prediction

To predict in silico ADME properties of the synthesised compounds **3a&b**, **4a–e** and **5a–e**. PreADME online surver^52^ was used. Human intestinal absorption (HIA), cell permeability of CaCo-2 cell and Madin-Darby Canine Kidney (MDCK) cell, plasma protein binding (PPB), blood brain barrier (BBB) and skin permeability (SP) were calculated.

### Metabolism prediction

The most important parameters used to measure metabolism and excretion were cytochrome P450 (CYP) isoforms. Metabolism prediction parameters for the tested compounds **3a&b**, **4a-e** and **5a–e** were examined using the Swissadme online server^53^.

### Statistical analysis

The obtained data was represented as means ± standard deviations (SD). Significant results were considered when *p˂0.05 or **p˂0.005 using a student’s *t-*test compared to reference drugs. The obtained values were represented as a result of triple independent experiments.

## Results and discussion

### Chemistry

#### General chemistry information

**Scheme 1.**
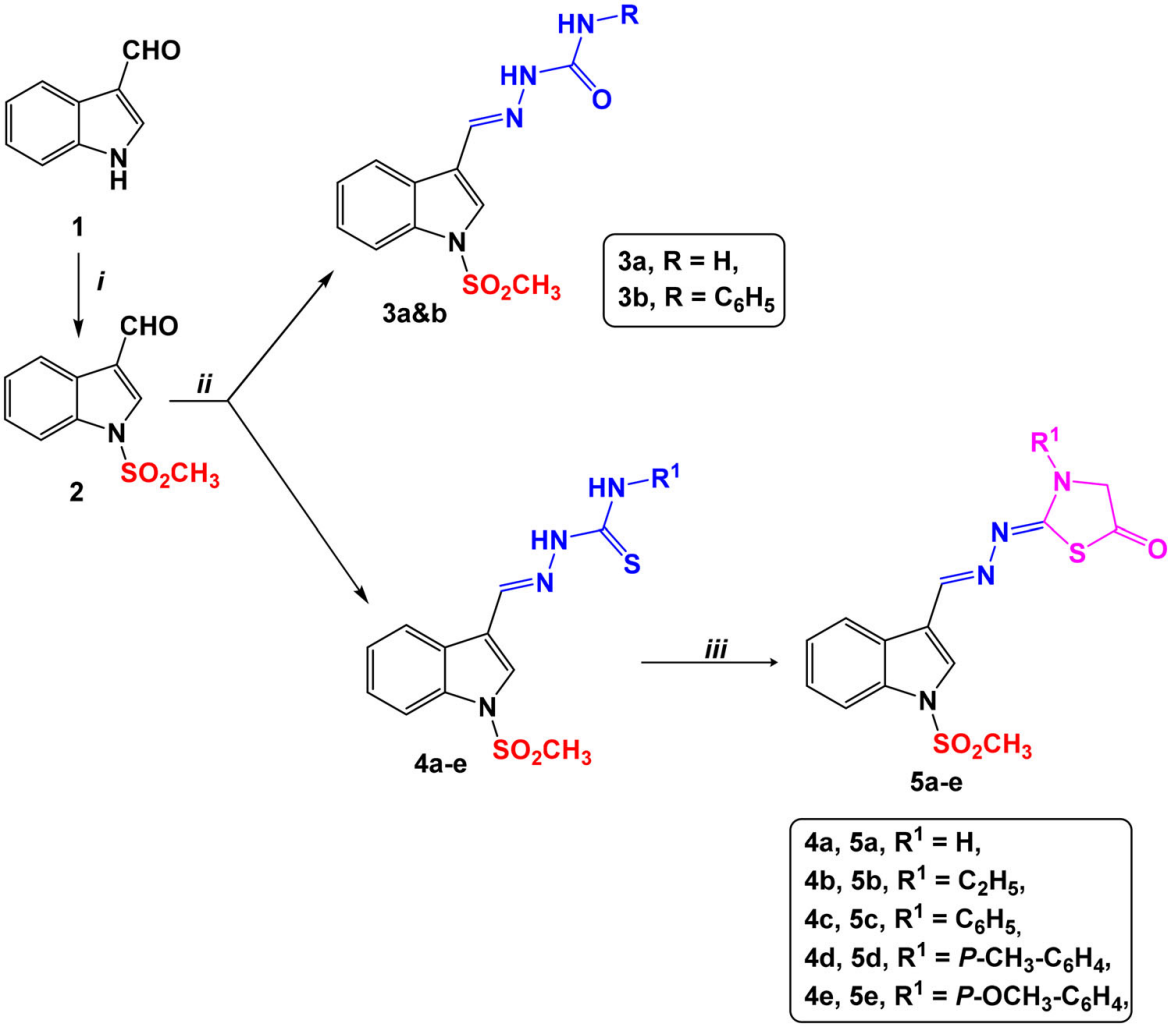
Synthetic routes for preparation of indole semicarbazide **3a&b**, thiosemicarbazide **4a–e** and thiazolidinone **5a–e** derivatives.

The synthetic procedures for semicarbazone derivatives **3a&b**, thiosemicarbazone derivatives **4a–e** and thiazolidinone derivatives **5a–e** were performed as depicted in Scheme 1.

Starting from indole-3-carboxaldehyde (**1**), *N*-methylsulfonylindole carboxaldehyde derivative **2** was prepared through an *N*-alkylation reaction using a strong base such as sodium hydride^54^. The synthesised key intermediate **2**, reacted with semi/thiosemicarbazides in the presence of absolute ethanol containing a few drops of DMF to obtain semi/thiosemicarbazone derivatives **3a&b/4a–e** via a condensation reaction. Afterward, the obtained thiosemicarbazone derivatives **4a–** were cyclized using ethyl chloroacetate and sodium acetate to provide the respective thiazolidinone derivatives **5a–e** at good yields ranging from 68 to 79%.

To confirm the chemical structure of the synthesised compounds **3a&b**, **4a–e,** and **5a–e**, ^1^H NMR, ^13^C NMR (DEPT-Q), mass spectral data and elemental analysis were determined.

IR spectra of compounds **3a&b,** and **4a–e** displayed stretching of NH and C = O/or C = S bonds at (3431–3122), (1696 and 1673), or (1129–1058) cm^−1^, respectively. Additionally, ^1^H NMR spectra for semi/thiosemicarbazone derivatives **3a&b/4a–e** displayed signals at *δ* 3.49–3.54 and 8.11–8.70 ppm corresponding to -SO_2_CH_3_ and azomethine –CH = N– protons, sequentially. Moreover, two D_2_O exchangeable singlet signals were observed at *δ* 7.50–11.78 ppm attributed to NH protons of compounds **3a&b** and **4a–e**.

^13^C NMR spectra of **3a&b** and **4a–e** showed peaks at *δ* (41.51–41.81), (156.50 and 157.10) or (176.18–177.94) ppm owing to carbons of SO_2_CH_3_, C = O or C = S, in a sequent.

On the other hand, the structure of thiazolidinone derivatives **5a–e** was confirmed using IR spectra through the appearance of an absorption band at 3429 cm^−1^ attributed to thiazolidinone NH in compound **5a** and a band in the range of 1620–1715 cm^−1^ characteristic of C = O in **5a–e**. Additionally, the disappearance of the NH group of the parent compounds in all derivatives **5a–e** confirmed the cyclisation process.

Another evidence for ring closure in compounds **5a–e** was obtained from ^1^H NMR spectra, in which a new singlet signal appeared at the range of *δ* 3.93–4.12 ppm attributed to CH_2_ protons, in addition to the disappearance of D_2_O exchangeable singlet signals for their parent compounds **4a–e**, while, the appearance of thiazolidinone NH proton of compound **5a** at *δ* 7.51 ppm.

Similarly, the ^13^C NMR spectra of **5a–e** showed chemical shifts for carbons of CH_2_ and C = O groups in a region of *δ* 32.59–33.53 ppm and *δ* 172.45–172.69 ppm, respectively. Additionally, mass spectral data for compound **5b** exerted its molecular ion peak at *m/z* 364.65 by intensity equals 18.87%.

##### Reagents & conditions

(i) CH_3_SO_3_Cl, NaH, DMF, RT, 3 h; (ii) semicarbazone derivatives for **3a&b**/or thiosemicarbazone derivatives for **4a–e**, abs. EtOH/drops DMF, reflux, 2–4 h; (iii) ClCH_2_COOEt, NaAc, abs. EtOH/drops DMF, reflux, 4–6 h.

#### (Z/E) stereochemical determination

To detect the relative configuration (*E* or *Z*) for the synthesised indole derivatives **3a&b**, **4a–e** and **5a–e**, 2D NMR NOESY (Nuclear Over Hauser Effect Spectroscopy) experiment was performed.

Compound **4b**, as a representative example, was chosen for the NOESY experiment and data obtained revealed the spatial correlation between = N–NH– proton at *δ* 7.43 ppm and azomethine –CH = N– proton at *δ* 8.35 ppm (Bond length of NOESY correlating bonds = 1.7 Å in *E*-form), and between indole H-2 at *δ* 8.22 ppm and azomethine proton (Bond length of NOESY correlating bonds = 4.5 Å in *E*-form). While, no spatial interaction was observed between azomethine proton/NH or azomethine proton/indole H-2 in *Z*-form (Bond length of NOESY correlating bonds >6 Å in *Z*-form). The obtained results suggested the *E*-configuration for the synthesised indole derivatives.

**Figure 2.**
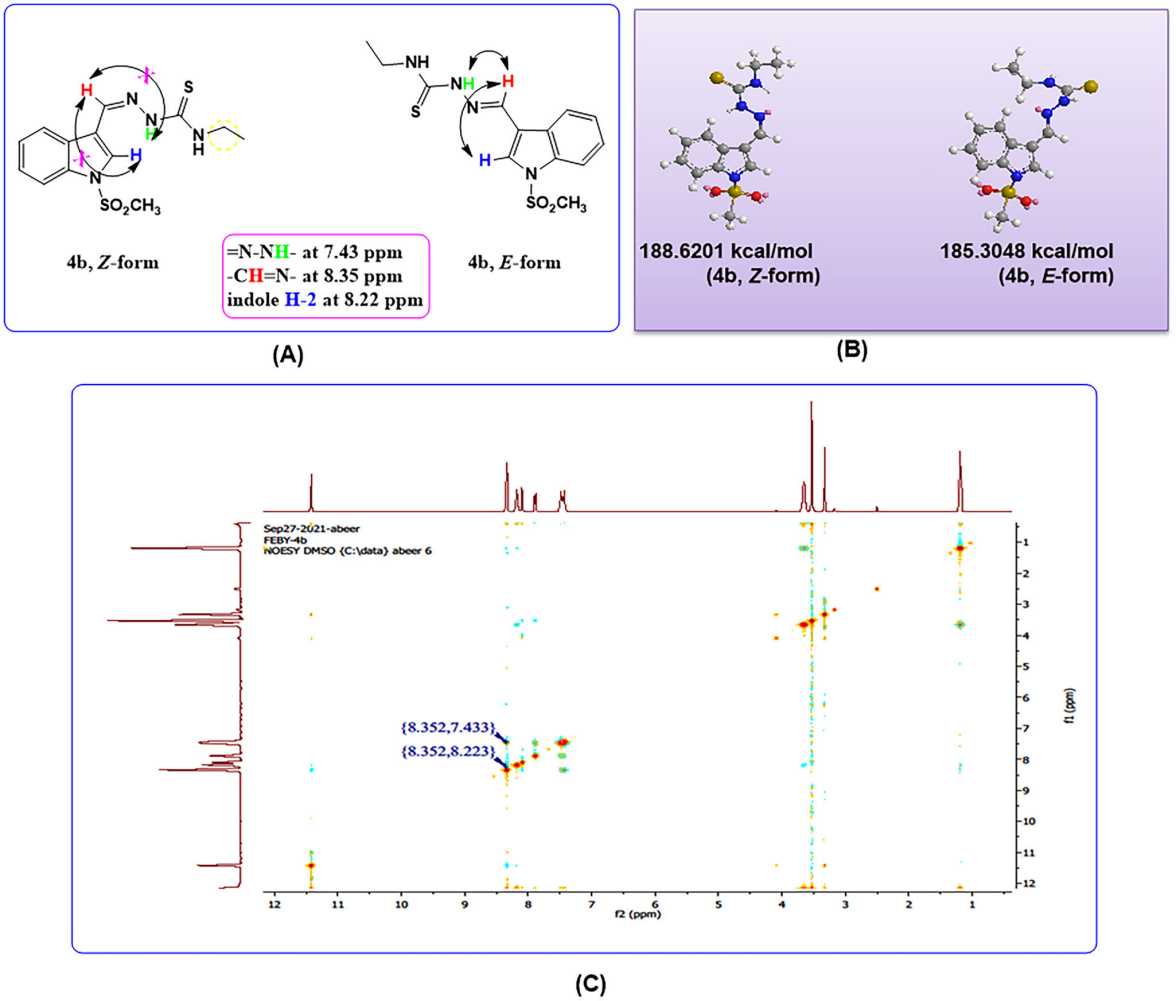
(**A**) 2D structure of **4b** *Z*- and *E*-forms with predicted correlations, (B) 3D structures of **4b** *Z*- and *E*-forms, (C) NOESY for compound **4b**.

Moreover, a theoretical method was performed to confirm the *E*-configuration for the synthesised derivatives using Chem3D Ultra 12.0 and MM2 properties. Total energy, the sum of stretch, bending, stretch-bend, torsion, non-1,4 Van Der Waals, 1,4 Van Der Waals, and dipole/dipole interactions, was calculated for each *Z*-/*E*-conformer. It was noticed that the total energy of the *E*-form exceeded that of the *Z*-form for all the target compounds, (Table 1 and Figure 2).

**Table 1.** Predicted total energy for *Z*- and *E*-forms of the target compounds **3a&b**, **4a–e** and **5a–e**.

Compd. No.	Total energy (Kcal/mol)		Compd. No.	Total energy (Kcal/mol)	
	*Z*-form	*E*-form		*Z*-form	*E*-form
**3a**	172.86	168.47	**4e**	384.02	184.85
**3b**	177.55	172.82	**5a**	194.40	188.92
**4a**	186.24	182.13	**5b**	202.84	192.42
**4b**	188.62	185.30	**5c**	215.54	204.54
**4c**	234.29	179.12	**5d**	211.25	204.24
**4d**	304.41	178.82	**5e**	221.18	210.16

### Biological evaluation

#### Antimicrobial activity screening

**Figure 3.**
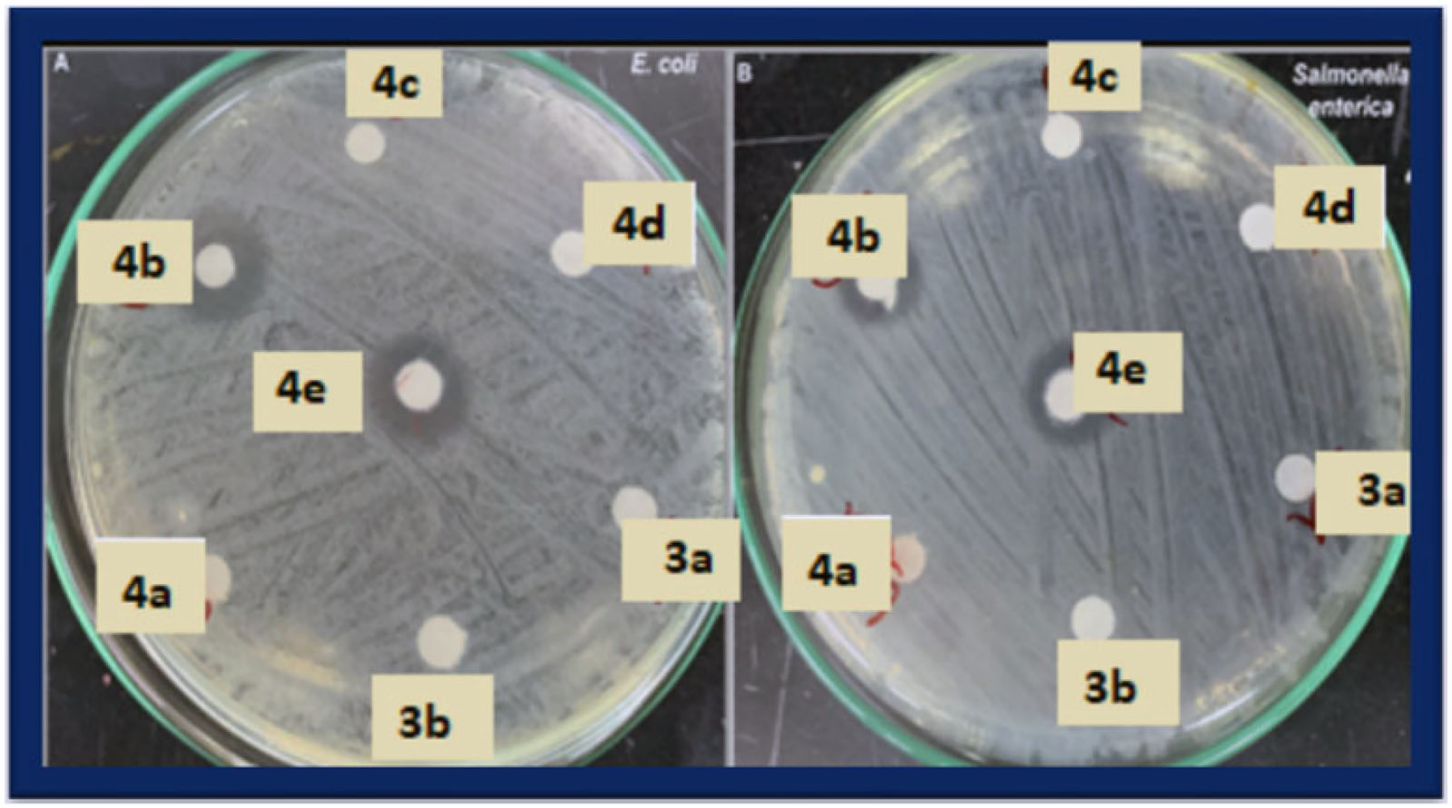
Positive antibacterial activity of compounds **4b** and **4e** against: (A) *E. coli*; and (B) *Salmonella enterica.*

All tested compounds were subjected to antimicrobial screening against Gram-positive bacteria, Gram-negative bacteria, and yeast fungus. The obtained data revealed that the ethylthiosemicarbazide derivative, **4b,** and *p*-methoxyphenylthiosemicarbazide compound, **4e** showed selective antibacterial activity against the Gram-negative bacteria, *E. coli*, and *Salmonella enterica*. Whereas, *p*-tolylthiazolidinone derivative **5d** exclusively exhibited bioactivity against *E. coli* with diameters ranging from 8 to 15 mm at 100 µg/disc concentration compared to the standard ciprofloxacin which had inhibition zones against all tested Gram-positive and Gram-negative bacteria with diameters’ range from 34 to 48 mm at a concentration of 20 µg/disc. Although our bioactive compounds showed modest antibacterial activity compared to the ciprofloxacin standard, further modifications to their structures could help increase their bioactivity or even broaden their antimicrobial spectrum of activity. On the other hand, the other nine compounds did not display antifungal or antibacterial activity, Figure 3. The result obtained was in accordance with what was reported about indole derivatives and their antimicrobial activity^4^^,^^31^. Thus, indole derivatives bearing thiazolidinone part and an aliphatic group such as Me or OEt showed the highest antimicrobial activity against both Gram-positive bacteria and Gram-negative bacteria^27^. Additionally, indole-hydrazone scaffold and methoxyphenyl substitution exhibited promising antibacterial activity, as reported by Nassar et al^31^.

#### Anti-oxidant activity using DPPH radical scavenging activity

The *in vitro* antioxidant activity of semicarbazides **3a&b**, thiosemicarbazides **4a–e** and thiazolidinone derivatives **5a, 5c** and **5e** were determined by measuring the reduction capacity of DPPH radicals spectrophotometrically at 540 nm. Trolox, a water-soluble analog of vitamin E, was used as a standard. By inspecting data in Table 2, it was found that the highly anti-oxidant compound between all the tested derivatives was phenylthiazolidinone **5c** with a value of 707.52 µM Trolox equivalent/mg. generally, thiosemicarbazide derivatives **4b** and **4d** showed high scavenging activity on DPPH with values of 426.65 and 389.82 µM Trolox equivalent/mg, in a sequent. Whereas, their thiazolidinone analogs, **5b** and **5d**, didn’t dissolve in DMSO nor in methanol. The turbidity of these samples hinders their appropriate measurement.

**Table 2.** DPPH free radical assay results of indole derivatives **3a&b**, **4a–e** and **5a–e** relative to Trolox.

Compd. No.	Test results (µM trolox equivalent/ 1mg sample ± SD)	Compd. No.	Test results (µM trolox equivalent/ 1mg sample ± SD)
**3a**	5.91 ± 1.87	**4e**	161.21 ± 7.98
**3b**	14.38 ± 0.85	**5a**	12.71 ± 1.16
**4a**	24.55 ± 1.61	**5b**	Not Detected
**4b**	426.65 ± 28.02	**5c**	707.52 ± 49.91
**4c**	263.29 ± 12.30	**5d**	Not Detected
**4d**	389.82 ± 19.25	**5e**	13.22 ± 1.89

Phenylthiazolidinone derivative **4c** showed an increase in anti-oxidant activity of about 263.29 µM Trolox equivalent/mg. Moreover, *p*-methoxyphenylthiosemicarbazide derivative **4e** exerted very high anti-oxidant activities (161.20 µM Trolox equivalent/mg) if compared to its thiazolidinone analogs **5e** (13.22 µM Trolox equivalent/mg).

The lowest scavenging activity on DPPH was observed in semicarbazone derivatives **3a** and **3b**, thiosemicarbazide derivative **4a**, its thiazolidinone derivative **5a** and *p*-methoxyphenylthiazolidinone derivative **5e** with values ranging from 24.55 to 5.91 µM Trolox equivalent/mg.

It is worth mentioning that between all tested series, thiosemicarbazide moiety increased the antioxidant activity except for phenylthiazolidinone derivative **5c**.

### Anti-inflammatory activity

#### In vitro measurements of TNF-α in RAW264.7 macrophage cells

The synthesised compounds were evaluated for their *in vitro* anti-inflammatory activity using RAW264.7 macrophage cells. The excellent anti-inflammatory activity was obtained by decreasing TNF-α fold, which indicates inhibition of TNF-α production in RAW264.7 cells.

**Figure 4.**
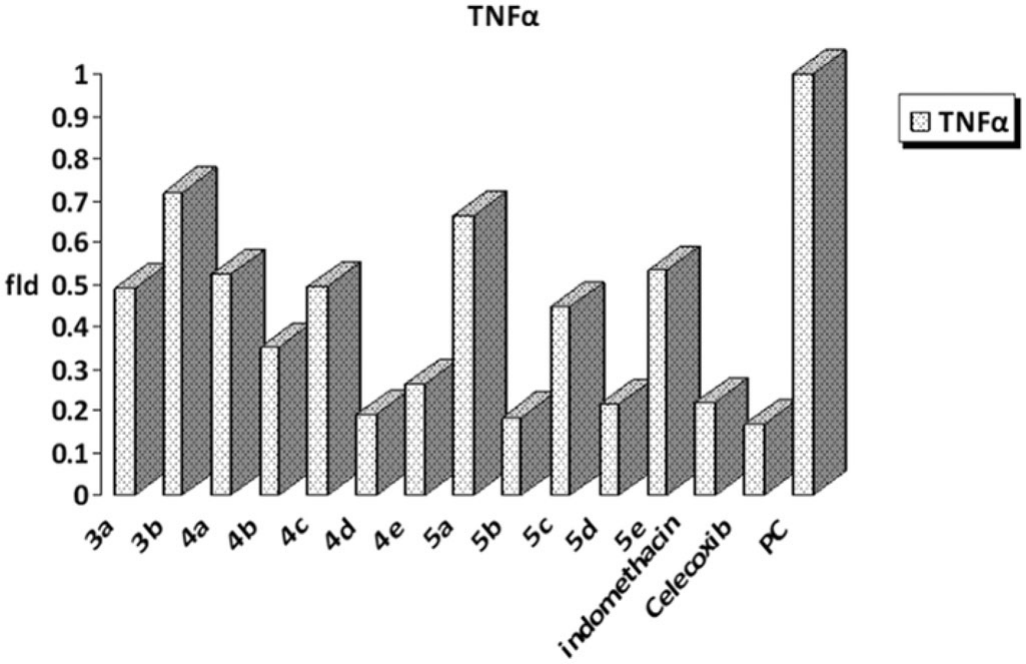
*In vitro* measurements of TNF-α in RAW264.7 macrophage cells in tested compounds and reference drugs.

The obtained results represented in Figure 4 revealed that compounds **4d**, **4e**, **5b,** and **5d** showed the highest anti-inflammatory activity by decreasing TNF-α to reach 0.19, 0.26, 0.18, and 0.21, respectively, relative to that of indomethacin (0.22) and celecoxib (0.16). The rest of the compounds showed fold changes ranging from 0.35 to 0.53. Both the phenyl semicarbazone derivative, **3b** and thiazolidinone derivative, **5a** exerted the lowest activity, showing fold changes equal to 0.71 and 0.66, respectively.

#### COX-1, COX-2 and 5-LOX inhibitory activity determination

All of the synthesised compounds **3a&b**, **4a–e** and **5a–e** were evaluated for their *in vitro* COX-1, COX-2, and 5-LOX inhibitory activities. Indomethacin, celecoxib, and zileuton were used as reference drugs for COX-1, COX-2, and 5-LOX, respectively. Colorimetric enzyme immunoassay (EIA) kits were done to screen for the isozyme-specific inhibition. To evaluate the effectiveness of the synthesised compounds against COX-1, COX-2, and 5-LOX enzymes, a minimum dose that causes 50% inhibition (IC_50_) was determined using serial concentrations (100, 10, 1, and 0.1 μM) for each compound. The COX-2 selectivity index values (S.I.) were calculated as [IC_50_ (COX-1)/IC_50_ (COX-2)] and compared to the standard drugs, indomethacin (a non-selective COX-inhibitor) and celecoxib (a selective COX-2 inhibitor).

From the obtained data, (Table 3), it was observed that all the tested compounds might be of good safety profile as they could inhibit the COX-1 enzyme at higher concentrations (IC_50_ = 3.95–32.02 uM) than indomethacin (IC_50_ = 0.38 uM).

**Table 3.** *In vitro* COX-1, COX-2 and 5-LOX inhibitory activity results and S.I. of the newly synthesised compounds **3a&b**, **4a–e** and **5a–e** with indomethacin, celecoxib, and ziluton as reference drugs.

Compd. No.	IC_50_ COX-1 (µM)^a^	IC_50_ COX-2 (µM)^a^	S.I^b^	IC_50_ 5-LOX (µM)^a^
**3a**	21.14 ± 0.60	27.14 ± 1.43	0.78	48.83 ± 2.58
**3b**	32.02 ± 0.91	22.01 ± 1.16	1.46	14.81 ± 0.78
**4a**	11.34 ± 0.32	4.05 ± 0.21	2.80	2.31 ± 0.12
**4b**	28.46 ± 0.80	86.15 ± 4.55	0.24	29.17 ± 1.54
**4c**	5.01 ± 0.14	1.61 ± 0.09	3.12	4.03 ± 0.21
**4d**	17.13 ± 0.49	7.212 ± 0.38	2.38	17.92 ± 0.95
**4e**	24.71 ± 0.70	0.819 ± 0.04	30.17	4.08 ± 0.22
**5a**	3.95 ± 0.11	6.67 ± 0.35	0.59	9.23 ± 0.49
**5b**	22.20 ± 0.63	89.22 ± 4.72	0.25	47.82 ± 2.53
**5c**	29.24 ± 0.83	8.06 ± 0.43	3.63	79.43 ± 4.20
**5d**	5.79 ± 0.16	0.67 ± 0.04	8.60	1.10 ± 0.06
**5e**	47.50 ± 1.35	4.88 ± 0.26	9.73	6.65 ± 0.35
Indomethacin	0.39 ± 0.01	11.37 ± 0.02	0.03	–
Celecoxib	7.25 ± 0.04	0.46 ± 0.02	15.65	–
Zileuton	–	–	–	0.58 ± 0.03

^a^The concentration of test compound produces 50% inhibition of COX-1, COX-2, and 5-LOX enzymes; ^b^The *in-vitro* COX-2 selectivity index (IC_50_ COX-1/IC_50_ COX-2).

On the other hand, the *p*-methoxyphenylthiosemicarbazide derivative **4e** and *p*-tolylthiazolidinone derivative **5d** showed high potency towards COX-2 enzyme inhibition with IC_50_ = 0.81 and 0.67 μM, sequentially, compared to celecoxib (IC_50_=0.46 μM). While compounds **4a**, **4c**, **4d**, **5a**, **5c,** and **5d** exerted moderate potency towards COX-2 enzyme inhibition with IC_50_ values ranging from 1.60 to 8.06 μM. The least activity was observed in ethylthiosemicarbazide derivative **4b** and its thiazolidinone derivative **5b** with IC_50_ = 86.15 and 89.22 μM, in a sequent.

Regarding COX-2 selectivity index values, all tested compounds exhibited COX-2 selectivity over COX-1 enzyme (S.I = 0.23–30.17) higher than that of indomethacin (S.I = 0.03). The thiosemicarbazide derivative, **4e**, showed a superior S.I. value (30.17) towards COX-2 over COX-1 enzyme than that of celecoxib (S.I.=15.65).

Moreover, compounds containing a thiazolidinone ring with electron donating groups (CH_3/_OCH_3_), **5d** and **5e**, had good COX-2 S.I values (8.59 and 9.72, in order). As a result, compounds **4e**, **5d,** and **5e** might have a safe gastric profile more than or nearly equal to that of celecoxib.

Concerning 5-LOX inhibitory activity results, compound **5d** (*p*-tolylthiazolidinone derivative) was the only one among all tested compounds that showed inhibitory activity against 5-LOX enzyme (IC_50_ = 1.10 μM) if compared to zileuton, the reference drug, (IC_50_ = 0.57 μM). Additionally, good to moderate 5-LOX inhibitory activity was observed in compounds **4a**, **4c**, **4e**, **5a,** and **5e** (IC_50_ = 2.31–9.23 μM). Dual COX-2/5-LOX inhibitory activity was achieved mainly by compound **5d**, from which, it might be expected to have anti-inflammatory activity as well as cardioprotective effect.

### Evaluation of ulcerogenic effect

#### Ulcerogenic liability

The most selective compounds, **4e** and **5d**, were shown to have an ulcerogenic impact when compared to the reference drug celecoxib and the ulcerogenic non-selective COX-2 inhibitor indomethacin, utilising a 50 mg/kg dosage for three consecutive days to show their gastrointestinal safety profile. The acquired findings are depicted in (Table 4). Our findings suggested that all tested compounds had a lower ulceration impact than celecoxib and indomethacin, the reference drugs, as evidenced by an ulcer index range (U.I. = 0.2–1.3) as compared to celecoxib (U.I. = 3.79) and indomethacin (U.I. = 6.8). The most selective molecule, *p*-methoxyphenyl thiosemicarbazide derivative **4e**, was also the least ulcerogenic (U.I. = 0.2). In addition, the *p*-tolylthiazolidinone derivative **5d**, demonstrated an excellent ulcer index (1.3) as compared to celecoxib (U.I. = 3.97).

**Table 4.** Ulcerogenic liability for the highest S.I. **4e**, **5d** and reference drugs celecoxib and indomethacin.

Compound	Ulcer index
**5d**	1.3^a,b^
**4e**	0.2^a,b^
Celecoxib	3.79^b^
Indomethacin	6.8

Data are expressed as mean of 6 rats ± SEM.

Multiple comparisons were done using one-way ANOVA test followed by Tukey-Kramer as *post hoc* test.

^a^Significantly different from indomethacin group (*p* < 0.05).

^b^Significantly different from celecoxib group (*p* < 0.05).

#### Stomach histopathological examination

**Figure 5.**
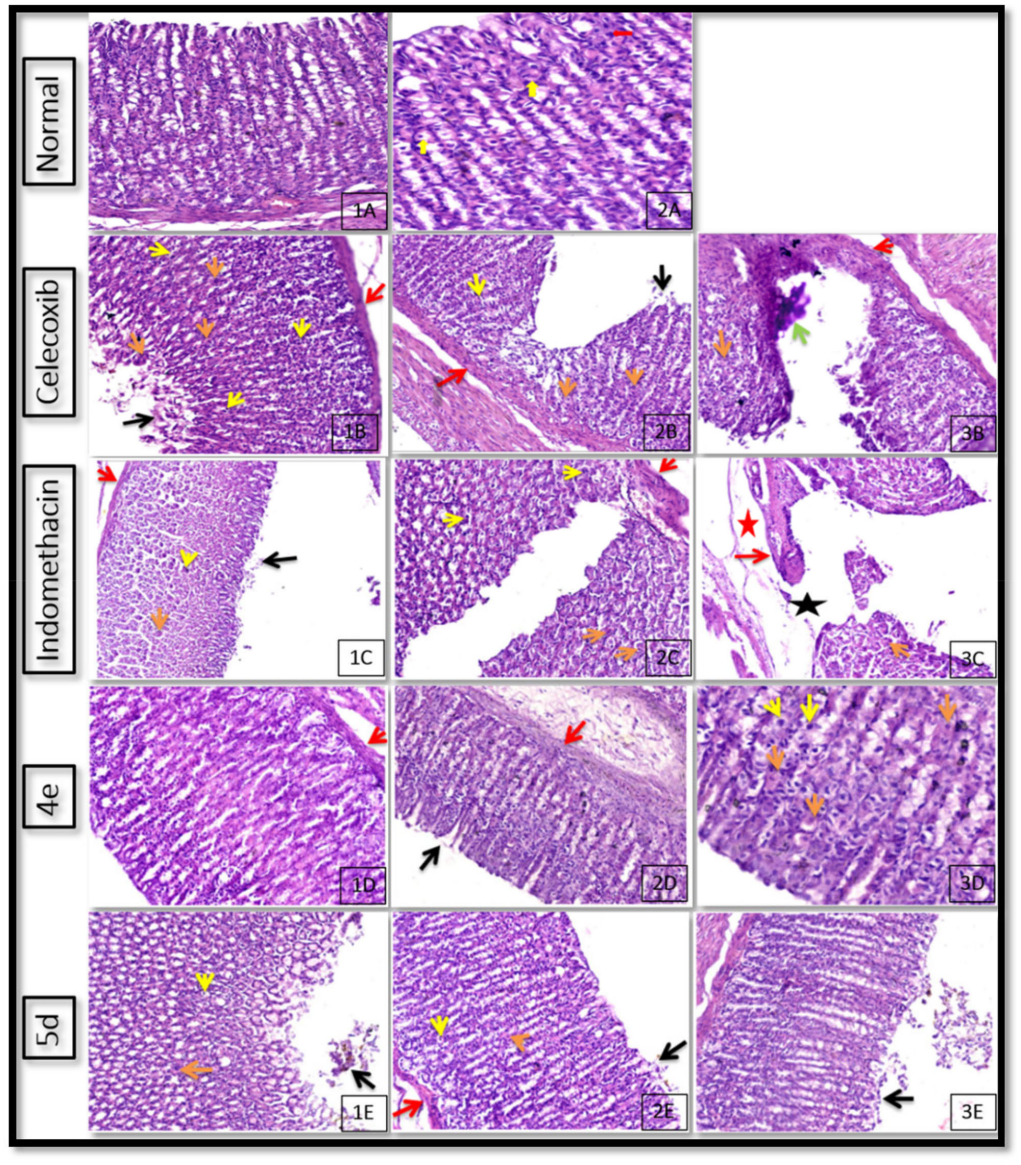
Effects of celecoxib, indomethacin, and the highest selective compounds **4e** and **5d** on the stomach histology (H & E X 400 & 200).

In order to compare the severity of lesions to those caused by the reference drugs celecoxib and indomethacin as well as to the lesions in the control negative group, evaluation of the ulcerogenic effect of tested compounds on various stomach portions through examination of variable histopathological lesions was carried out. Normal control rats showed normal gastric mucosa with a normal pattern and architecture of the gastric gland (H & E X 200) (Figure 5(1A)); another view showed normal gastric mucosa with normal pattern and architecture of gastric gland with preservation of parietal cells (yellow arrow) and chief cells (red arrow) (H & E X 400) (Figure 5(2A)). On the other hand, celecoxib group showed gastric tissue with superficial mucosal erosion showing disrupted mucous layer (black arrow), degenerated mucosal epithelial cells, decrease number of parietal cells (orange arrow) in superficial mucosa, average number of parietal and chief cells (yellow arrow) in deep mucosa and average muscularise mucosa (red arrow) (H & E X 200) (Figure 5(1B)); another view indicated mucosal ulceration showing destructed mucus layer, degenerated mucosal epithelial cells in the base of the ulcer, absence of parietal cells and chief cells in the base of the ulcer with decreasing number in adjacent mucosa, and average muscularise mucosa (red arrow) (H & E X 200) (Figure 5(2B)); Also the view in deep mucosa revealed marked mucosal ulceration showing completely destructed mucous layer, complete loss of parietal and chief cells, marked inflammations and beginning of fibrosis in ulcer bases (increase thickening of muscularis mucosa), devitalised mucosal epithelial cells (green arrow) in ulcer margins and adjacent mucosa, with decreasing number of parietal cells and chief cells in adjacent mucosa (H & E X 200) (Figure 5(3B)). In conclusion, celecoxib administration resulted in multiple complete ulcers and superficial mucosal erosions with disrupted mucous layer, average number of parietal and average chief cells in deep mucosa with average muscularise mucosa, degenerated mucosal epithelial cells in the base of the ulcer, marked inflammations and beginning of fibrosis in ulcer bases, and devitalised mucosal epithelial cells in ulcer margins and adjacent mucosa.

While indomethacin administration viewed gastric tissue with superficial mucosal erosion showing disrupted mucous layer (black arrow), degenerated mucosal epithelial cells, edoema and decrease number of parietal cells (orange arrow) in superficial mucosa, average number of parietal and chief cells (yellow arrow) in deep mucosa and average muscularise mucosa (red arrow) (H & E X 200) (Figure 5(1C)); another view showed gastric tissue with marked mucosal ulceration showing marked congested vessels in the base of the ulcer, decreasing number of parietal cells in adjacent mucosa, and inflamed thickened muscularise mucosa (red arrow) (H & E X 200) (Figure 5(2C)); another view showed ulcer bases with perforated mucosal ulcer (black star) showing marked inflammations, edoema (red star), beginning of fibrosis, devitalised mucosal epithelial cells in ulcer base and adjacent mucosa, with decreasing number of parietal cells and chief cells in adjacent mucosa, inflamed thickened muscularise mucosa (H & E X 200) (Figure 5(3C)). In conclusion, indomethacin administration resulted in multiple complete and perforating ulcers and superficial erosions, marked mucosal ulceration showing marked congested vessels in the base of the ulcer, decreasing number of parietal cells in adjacent mucosa, and inflamed thickened muscularise mucosa as well as ulcer bases with a perforated mucosal ulcer with marked inflammations, edoema, beginning of fibrosis, devitalised mucosal epithelial cells in ulcer base and adjacent mucosa, with decreasing number of parietal cells and chief cells in adjacent mucosa, inflamed thickened muscularise mucosa.

Scanning the stomach of the rats treated with the tested compounds **4e** and **5d** showed the following:

*For compound* ***4e****:* gastric tissue showed retaining of the mucous layer (black arrow), regenerated mucosal epithelial cell layers, preserved a number of parietal cells (orange arrows) and chief cells (yellow arrow), with minimal mucosal, submucosal edoema, inflammations and average muscularis mucosa (red arrow) (H & E X 200 & 400) (Figure 5(1D–3D)). In conclusion, administration of the *p*-methoxythiosemicarbazide derivative **4e** resulted in partial loss of mucous layer with mild superficial erosions accompanied by mucosal epithelial cell layers’ regeneration, preserved parietal cells and chief cells in superficial and deep mucosa, minimal mucosal, submucosal edoema, inflammations and average muscularis mucosa.

*For compound* ***5d****:* gastric tissue showing minimally disrupted mucous layer (black arrow), regenerated mucosal epithelial cells layers with minimal residual surface epithelial erosions, preserved number of parietal cells (orange arrows) and chief cells (yellow arrow), with mild mucosal, submucosal edoema and inflammations and minimally decrease thickening of muscularis mucosa (red arrow) (H & E X 200) (Figure 5(1E–3E)). In conclusion, administration of the *p*-tolylthiazolidinone derivative **5b** resulted in partial loss of the mucous layer with mild, superficial erosions, preserved number of parietal cells and chief cells in superficial and deep mucosa, mild mucosal, submucosal edoema and inflammations and minimally decrease thickening of muscularis mucosa.

## Cardiovascular evaluation

### Assessment of the cardiac function biomarkers

It has been previously noted that celecoxib has cardio-toxicity in experimental rats^55^. Heart function indicators such as Troponin I, Creatine kinase-MB (CK-MB), and lactate dehydrogenase (LDH) were assessed in the current investigation to determine how the heart responded to the most active and most selective target compound, **5d**. Additionally, a comparison of the assessed compound **5d** to celecoxib as a reference standard was done regarding the study its histopathological changes. All of the findings were displayed in (Table 5). The findings showed that the injection of celecoxib caused a significantly higher level of the serum cardiac biomarkers Troponin I, CK-MB, and LDH when compared to normal control rats. Contrarily, animals treated with thiazolidinone derivative, **5d**, had substantially lower serum levels of these indicators than rats treated with celecoxib. These findings demonstrated that, in comparison to celecoxib, compound **5d** carries a considerably lower risk of cardiovascular damage.

**Table 5.** Cardiac effects of compound **5d** and the reference drug celecoxib.

Compound	LDH (U/L)	CK-MB (ng/ml)	Troponin I (ng/ml)
Control	340 ± 23	0.015 ± 0.005	0.0015 ± 0.0005
Celecoxib	690 ± 08^a^	0.635 ± 0.065^a^	0.1500 ± 0.0420^a^
**5d**	536 ± 28^a,b^	0.315 ± 0.035^a,b^	0.0015 ± 0.0005^b^

Data are expressed as mean of 6 mice ± SEM.

Multiple comparisons were done using one-way ANOVA test followed by Tukey-Kramer as *post hoc* test.

(1) Control group receiving only buffer. (2) Celecoxib group was given in a dose (50 mg/kg, p.o.) for 2 weeks. (3) **5d** was given in a dose of (50 mg/kg/day, p.o.) for 2 weeks.

^a^Significantly different from control group (*p* < 0.05).

^b^Significantly different from celecoxib group (*p* < 0.05).

### Histopathological examination of the heart

**Figure 6.**
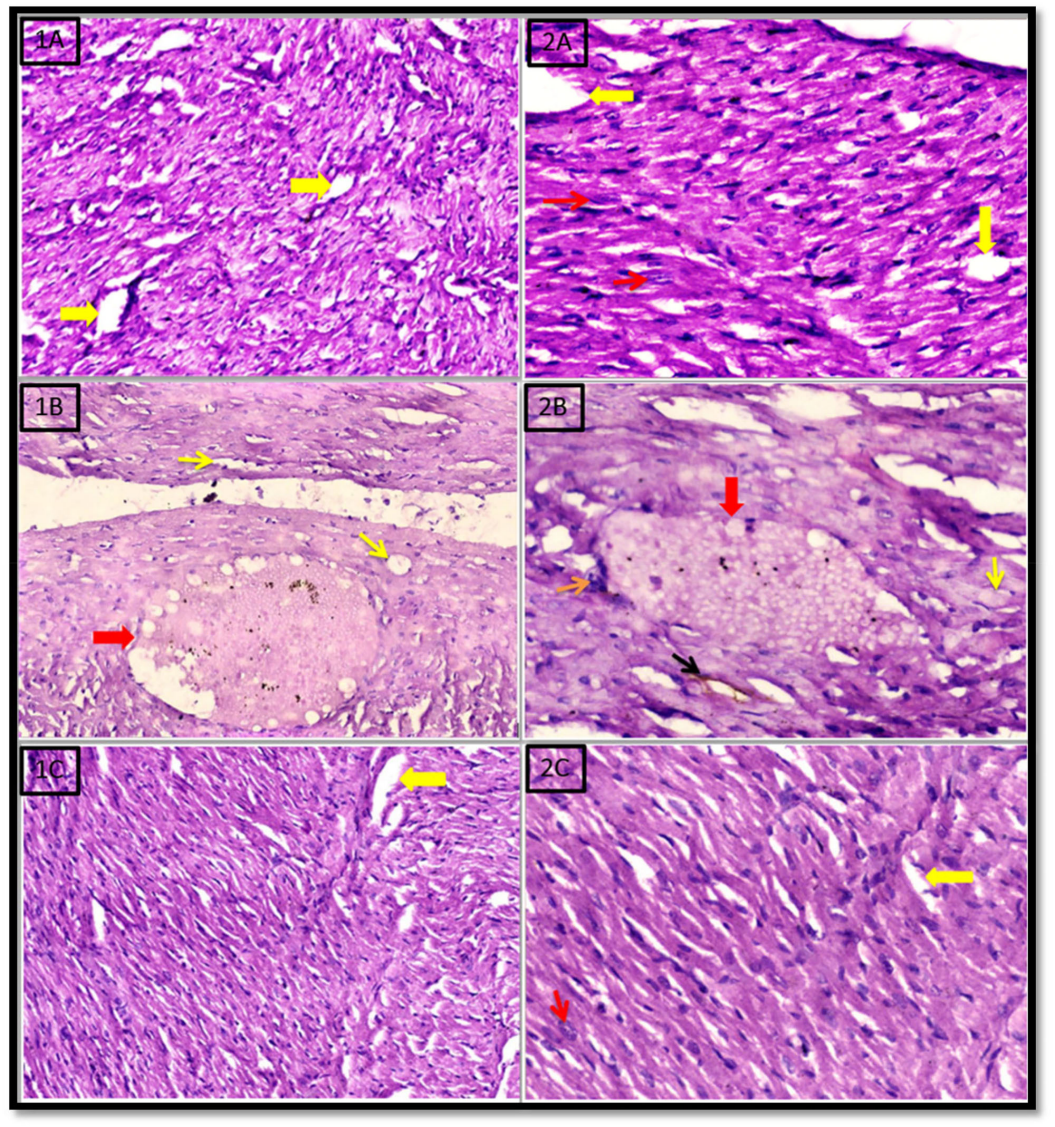
Histopathological examination of the heart in rats treated with control, celecoxib and the target compound **5d**.

Histopathological examination of the heart muscle of the control group shows a normal pattern of cardiac muscle fibres with normal cigar shaped nuclei (red arrows) and normally arranged blood vessels (yellow arrows) (H & E X 200 & X 400) (Figure 6(1A and 2A)). On the contrary, the heart section obtained from celecoxib group showed markedly inflamed cardiac muscle fibres with vacuolar degenerated myocytes (yellow arrow), edoema and dilated ectatic blood vessels containing plasma fluid (red arrow) (H & E X 200) (Figure 6(1B)); another view revealed markedly inflamed cardiac muscle fibres with vacuolar degenerated myocytes (yellow arrow), degenerated myocytes with brown pigmentation (black arrow) edoema, inflammatory cells (orange arrow) and dilated ectatic blood vessels containing plasma fluid (red arrow) (H & E X 400) (Figure 6(2B)). In conclusion, prolonged administration of celecoxib resulted in scattered inflamed cardiac muscle fibres with vacuolar degenerated myocytes with brown pigmentation, edoema as well as markedly congested and dilated ectatic blood vessels containing plasma fluid in the heart muscle. Alternatively, cardiac histopathological examination of the thiazolidinone derivative **5d** treated group revealed little or no toxic effects on the heart and showed viable cardiac muscle fibres retained to their normal pattern with regenerated myocytes and normal arranged blood vessels (yellow arrows) (H & E X 200) (Figure 6(1C)); another view revealed cardiac muscle fibres retained to their normal pattern with regenerated myocytes, normal cigar shape nuclei (red arrow) and normal arranged blood vessels (yellow arrows) (H & E X 400) (Figure 6(2C)).

### Molecular docking study

In this study, the mode of action of N-methylsulfonyl indole derivatives **3a&b**, **4a–e,** and **5a–e** as novel COX‐2, and 5-LOX inhibitors were determined via molecular docking studies. Docking of COX-2 ligand, celecoxib, and 5-LOX ligand, arachidonic acid, inside the binding site of COX‐2 (Protein Data Bank code: 3ln1) and 5-LOX (Protein Data Bank code: 3V99) was performed to compare between the key structural features for both ligands and the designed candidates. The docking energy affinity (Kcal/mol), interactions and amino acid residues indicated the binding mode for the developed new compounds such as COX‐2 and/or 5-LOX inhibitors.

**Figure 7.**
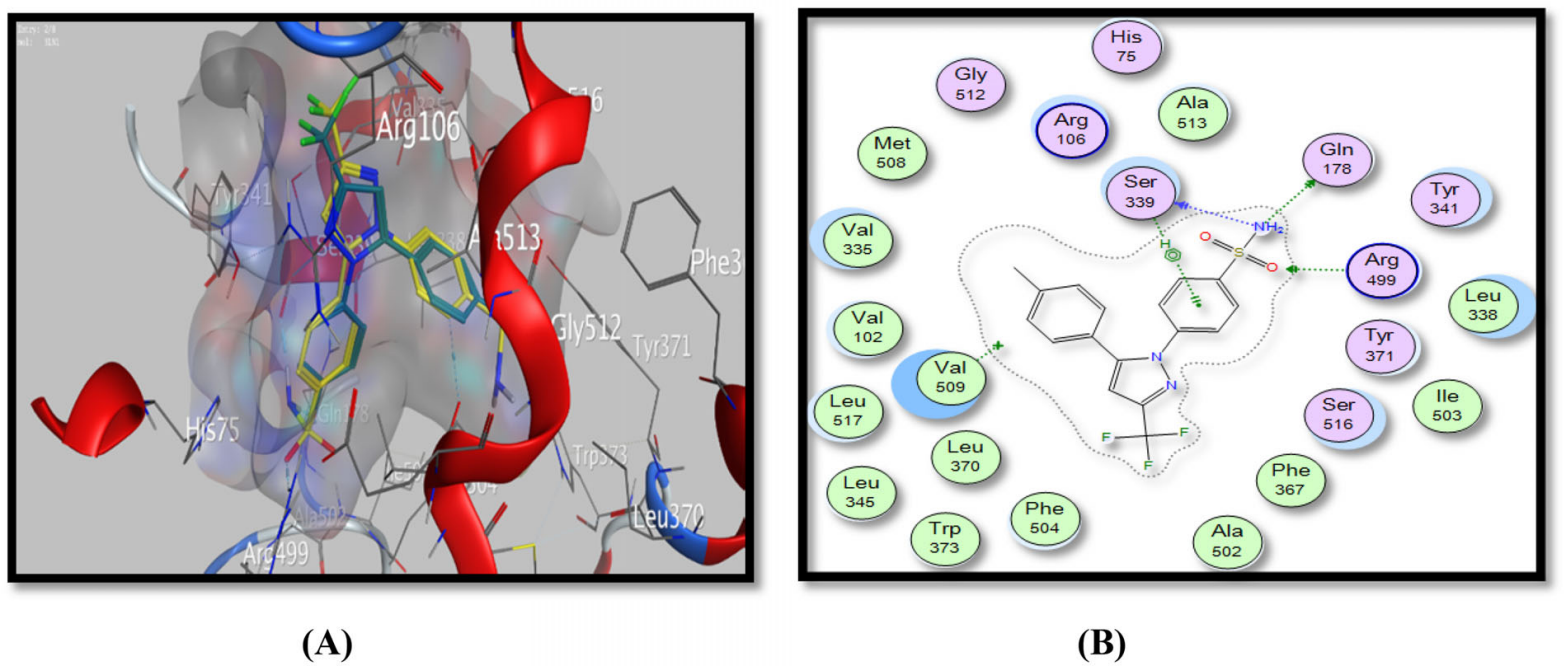
Binding mode of celecoxib inside COX-2 active site, (A) 3D visualisation of co-crystallised celecoxib ligand (yellow colour) superimposed with redocked celecoxib (cyan colour), indicating good fitting inside the pocket, (B) 2D binding mode of celecoxib inside COX-2 active site showing three H-bonding interactions with Arg499, Gln178, and Ser339 amino acid residues and one arene-H interaction with Ser339 amino acid residue.

Regarding the COX-2 docking study, the docking study of celecoxib, a selective COX-2 inhibitor, showed a binding energy of −7.52 Kcal/Mol. It formed three hydrogen bonding interactions with Gln178, Ser339 and Arg499 amino acid residues with NH_2_ and SO_2_ groups and one arene-H interaction with *P-*SO_2_NH_2_Ph moiety, (Figure 7, Table 6).

**Table 6.** Results of molecular docking study for synthesised indole derivatives ligands of COX-2 and 5-LOX enzymes.

Compd. No.	COX-2				5-LOX			
	E Score (Kcal/Mol)	Interactions	Functional group	AA (Distance Ǻ)	E Score (Kcal/Mol)	Interactions	Functional group	AA (Distance Ǻ)
Ligand	−7.5299	H-donor H-donor H-acceptor Arene-H	NH_2_ NH_2_ SO_2_ *P*-SO_2_NH_2_-Ph	Gln178 (2.69) Ser339 (2.65) Arg499 (3.04) Ser339 (3.84)	−7.8201	H-acceptor H-acceptor	–COO^-^ –COO^-^	His432 (2.69) His600 (2.65)
**3a**	−5.6512	H-donor	NH_2_	Met508 (4.35)	−6.0383	–	–	–
**3b**	−4.4368	H-acceptor Arene-H	C = O Ph	Arg499 (2.53) Ser339 (4.50)	−6.8655	H-acceptor	SO_2_CH_3_	His367 (3.13)
**4a**	−5.6976	H-acceptor H-acceptor	C = S C = S	Val509 (4.41) Gly512 (3.94)	−7.1936	H-donor H-acceptor	NH SO_2_CH_3_	Gln363 (3.11) Asn554(3.27)
**4b**	−2.6296	–	–	–	−6.6112	–	–	–
**4c**	−4.5533	H-acceptor Arene-H	C = S Indole-Ph	Ser516 (3.90) Ser339 (4.09)	−7.358	H-acceptor Arene-H	C = S Ph	His600 (3.78) His432 (4.30)
**4d**	−4.3818	H-donor H-acceptor H-acceptor	SO_2_CH_3_ C = S SO_2_CH_3_	Ser516 (2.39) Arg499 (4.04) Ser516 (2.93)	−6.9609	H-acceptor H-acceptor	C = S SO_2_CH_3_	Gln557 (4.26) His432 (2.92)
**4e**	−6.3765	H-acceptor H-acceptor H-Arene Arene-H Arene-H	C = S SO_2_CH_3_ SO_2_CH_3_ Indole-Ph Indole-Pyrrol	Ser516 (3.93) Phe504 (2.97) His75 (4.35) Ser339 (4.32) Ser339 (4.36)	−7.6282	H-acceptor	SO_2_CH_3_	His367 (3.27)
**5a**	−5.5167	H-donor H-donor H-arene	C = O NH CH_2_	Met508 (3.35) Met508 (3.07) Tyr371 (4.09)	−7.7883	H-donor	SO_2_CH_3_	His372 (3.75)
**5 b**	−2.4026	Arene-H	Indole-Pyrrol	Ser339 (3.77)	−6.5923	–	–	–
**5c**	−4.6980	H-donor Arene-H	Thiazolidinone-S Indole-Pyrrol	Val 509 (3.62) Ser339 (3.64)	−6.5977	H-donor	Thiazolidinone-S	Lys 409(4.06)
**5d**	−6.4414	Arene-H	*p*-tolyl	Ser339 (3.91)	−9.9152	Arene-H Arene-H	Indole-Ph Indole-Ph	His432 (4.08) His600 (3.49)
**5e**	−5.2844	Arene-H Arene-H	Indole-Ph Indole-Pyrrol	Ser339 (4.33) Ser339 (4.11)	−7.2508	H-acceptor	SO_2_CH_3_	His432 (2.97)

**Figure 8.**
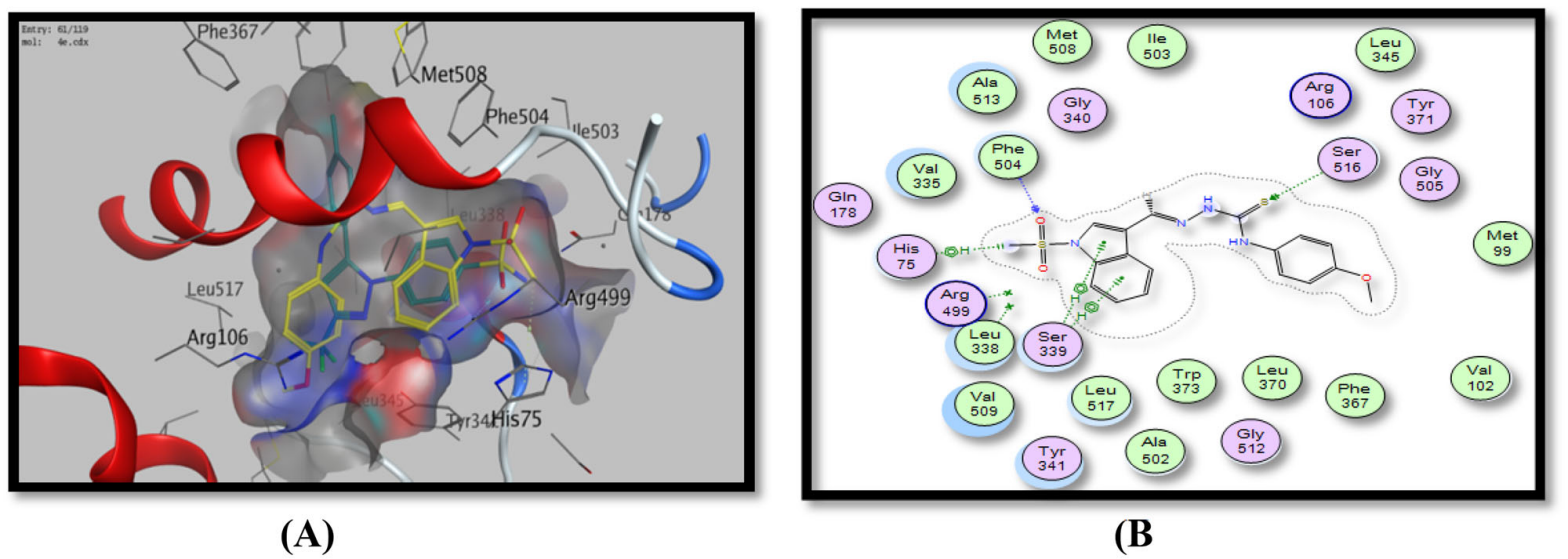
Binding mode of 4e inside COX-2 active site, (A) 3D visualisation of **4e** (yellow colour) superimposed with celecoxib (cyan colour), indicating good fitting inside the pocket, (B) 2D binding mode of **4e** inside COX-2 active site showing two H-bonding interactions with Ser516 and Phe504 amino acid residues and three arene-H interactions with Ser339 and His75 amino acid residues.

**Figure 9.**
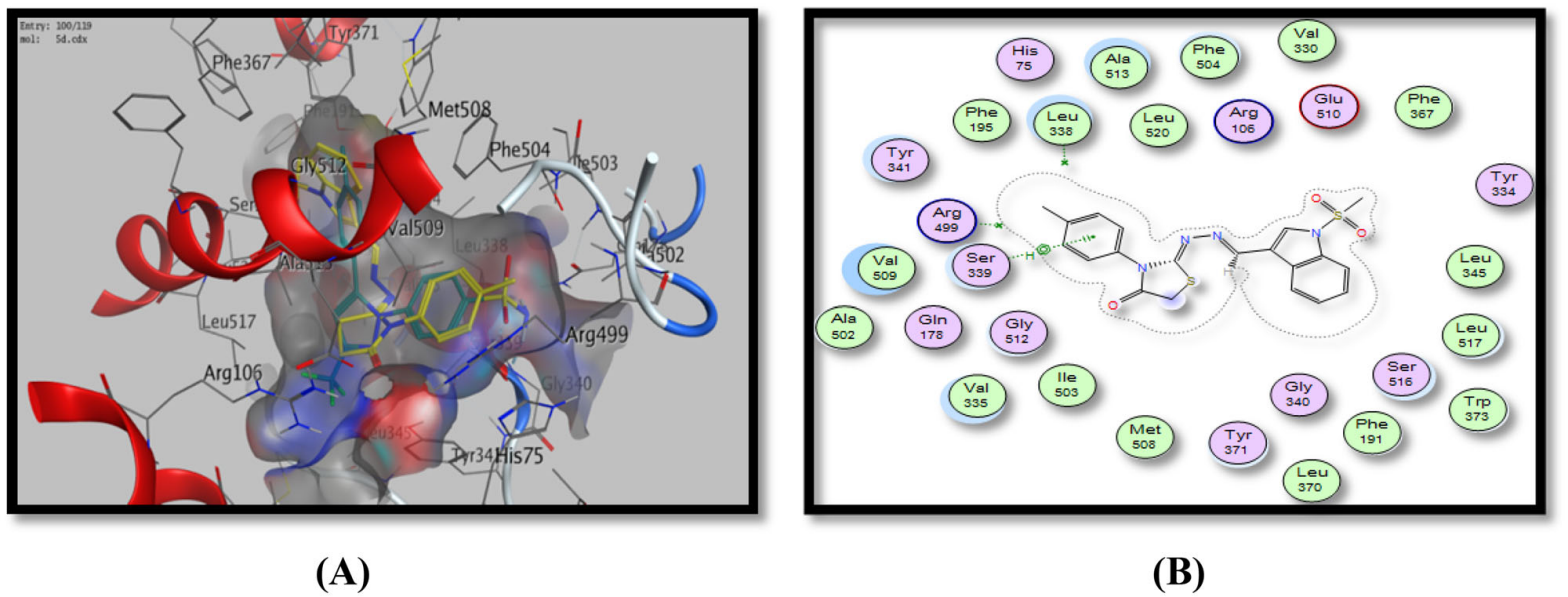
Binding mode of **5d** inside COX-2 active site, (A) 3D visualisation of **5d** superimposed with celecoxib, indicating good fitting inside the pocket, (B) 2D binding mode of **5d** inside COX-2 active site showing one arene-H interaction with Ser339 amino acid residue.

Additionally, docking studies for testing derivatives revealed that the energy of binding interactions ranged from −6.44 to −2.40 Kcal/Mol, and formed one to five binding interactions or without any interactions at all. The most active COX-2 inhibitors, *p*-methoxyphenylthiosemicarbazide derivative, **4e** and *p-*tolylthiazolidinone derivative, **5d** showed the highest energy scores of −6.37 and −6.44 Kcal/Mol and formed five and one binding interaction(s), respectively, with Ser516, Phe504, His75 and Ser339 amino acid residues with indole ring (phenyl and pyrrole), C = S and SO_2_CH_3_ pharmacophores, in addition to a p-tolyl moiety in compound **5d**, (Figures 8 and 9, Table 6).

On the other hand, the last two derivatives, as COX-2 inhibitors, were ethyl derivatives of thiosemicarbazide, **4b,** and thiazolidinone, **5b**. They revealed binding energy scores of −2.62 and −2.4026, sequentially. Compound **4b** didn’t form any type of binding interaction, while compound **5b** exerted one arene-hydrogen interaction between the pyrrole ring of indole and Ser339 amino acid residue, (Table 6).

**Figure 10.**
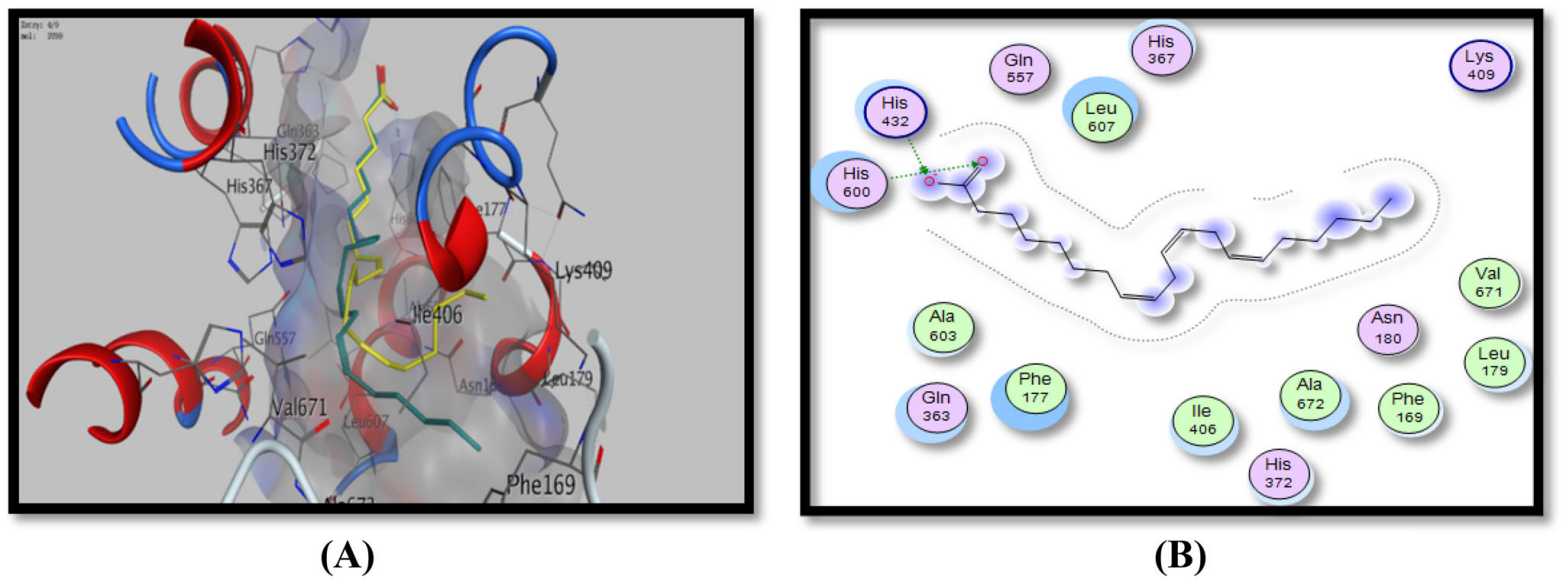
Binding mode of arachidonic acid inside 5-LOX active site, (A) 3D visualisation of co-crystallised arachidonic acid (yellow colour) superimposed with redocked arachidonic acid (cyan colour), indicating good fitting inside the pocket, (B) 2D binding mode of arachidonic acid inside 5-LOX active site showing two H-bonding interactions with His432 and His600 amino acid residues.

Concerning docking studies inside the 5-LOX enzyme, data obtained revealed that the active site of the enzyme formed two hydrogen bonding interactions between His432 and His600 amino acid residues and a –(-COO^-^) group of arachidonic acid, the redocked co-crystallised ligand compound with a binding energy score of −7.82 Kcal/Mol (Figure 10, Table 6).

**Figure 11.**
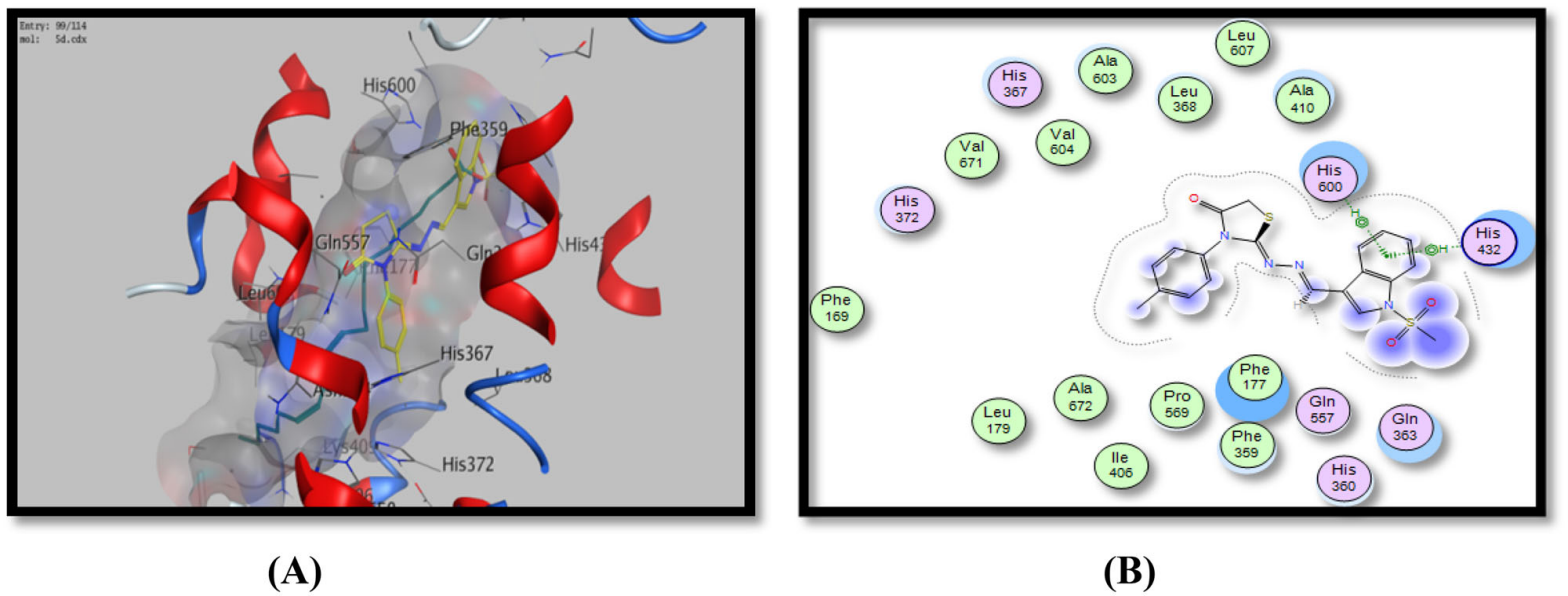
Binding mode of **5d** inside 5-LOX active site, (A) 3D visualisation of **5d** (yellow colour) superimposed with redocked arachidonic acid (cyan colour), indicating good fitting inside the pocket, (B) 2D binding mode of **5d** inside 5-LOX active site showing two H-bonding interactions with His432 and His600 amino acid residues.

The most active *p-*tolyl thiosemicarbazide derivative as a 5-LOX inhibitor, **5d**, had the highest binding energy score of −9.91 Kcal/Mol, higher than that of the ligand. It had two hydrophobic interactions between the phenyl of the indole ring and His432 and His600 amino acid residues (the same as that of the ligand) (Figure 11, Table 6).

Moreover, most of the tested derivatives showed energy binding interactions nearly equal to the ligand from −7.78 to −7.19 Kcal/Mol and formed binding interactions of one to two with Gln363, Asn554, His600, His432, His367, and His372 amino acid residues, (Table 6).

On the other hand, semicarbazide derivatives **3a&b**, ethylthiosemicarbazide derivative **4b**, *p*-tolylthiosemicarbazide **4d,** and ethyl or phenyl thiazolidinone derivatives **5b&c** showed energy binding interactions between −6.96 to −6.03 Kcal/mol, formed one or two binding interaction(s) with His367, Gln557, His432, and Lys 409 amino acid residues or without any binding interactions as in **3a**, **4b,** and **5b**, (Table 6).

In conclusion, the molecular docking study results of COX-2 and 5‐LOX enzymes indicated that the *p-*methoxy derivative of thiosemicarbazide **4e** had COX-2 binding activity, while the *p*-tolyl derivative of thiazolidinone **5d**, showed dual COX-2/5-LOX binding affinities. Both of them contain an electron-donating group in the *para* position of the phenyl ring.

## Computational analysis

### Molecular properties and drug-likeness

Lipiniski’s rule of five (RO5) analysis was used in this study to predict the biochemical properties of the synthesised target compounds. All compounds achieved Lipinisk’s rule of five standard values with a violation number of zero, (Table 7). Thus, the molecular weight of the target derivatives ranged from 280–442 g/Mol. That means less than the standard value of 500 g/Mol. Also, both hydrogen-bond acceptor and donor of tested compounds were in the range of 4 − 7 and 0–3, respectively, they didn’t exceed the acceptable values (No. HBA <10 and No. HBD <5). Moreover, the number of rotatable bonds, which indicate molecular flexibility, was from 3 to 5 (standard value <10). Lipophilic indicator LogP (octanol/water) partition co-efficient was evaluated for the target compounds. All showed high to moderate membrane permeability with partition co-efficient values in the range of 0.74–3.13 (within the acceptable range, <5).

**Table 7.** Molecular properties and drug-likeness results for the synthesised indole derivatives **3a&b**, **4a–e** and **5a–e**.

Compd. No.	*^a^*M.wt. (g/Mol)	*^b^*HBA	*^c^*HBD	*^d^*Rotatable bonds	*^e^*Mol. Logp	*^f^*TPSA (Å)	n-Violation	Drug-likeness score
**3a**	280.31	4	3	3	0.93	106.56	0	−0.07
**3b**	356.41	4	2	4	3.01	92.56	0	−0.27
**4a**	296.38	4	3	4	1.48	89.49	0	−0.21
**4b**	324.43	4	2	6	2.23	75.49	0	−0.39
**4c**	372.48	4	2	6	2.91	75.49	0	−0.30
**4d**	386.50	4	2	6	3.36	75.49	0	−0.20
**4e**	402.50	5	2	7	2.97	84.73	0	−0.04
**5a**	336.40	6	1	3	0.74	92.90	0	−0.61
**5b**	364.45	6	0	4	1.36	84.11	0	−0.68
**5c**	412.50	6	0	4	2.69	84.11	0	−0.55
**5d**	426.52	6	0	4	3.13	84.11	0	−0.66
**5e**	442.52	7	0	5	2.74	93.35	0	−0.63

*^a^*Molecular weight, *^b^*number of hydrogen-bond acceptors, *^c^*number of hydrogen-bond donors, *^d^*number of rotable bonds, *^e^*octanol/water partition coefficient, *^f^*topological polar surface area.

Prediction of oral bioavailability was determined from polar surface area (PSA) which should be less than 140 Ǻ. This was achieved by the target compounds (75 − 106 Ǻ). On the other hand, drug-likeness scores were acceptable for the target compounds, especially those containing semicarbazone moiety **3a** (-0.07) and for *p*-methoxyphenyl thiosemicarbazide derivative **4e** (-0.04).

### Bioactivity prediction

Interaction between synthesised compounds and certain drug targets might be a tool for their bioactivity prediction. From these drug targets, a G-protein coupled receptor (GPCR), an ion channel modulator, a kinase inhibitor, a nuclear receptor ligand, and protease inhibitor were studied. To consider the activity of the synthesised compounds, they should possess values >0.00 to be good lead structure, −0.50 to 0.00 as moderately bioactive, or < −0.50 to be inactive.

By inspecting the results obtained (Table 8), it was found that semicarbazone derivatives **3a** and **3b** showed good lead structures for G-protein coupled receptor (GPCR), while the rest of the compounds had moderate bioactivity.

**Table 8.** Bioactivity prediction for the synthesised indole derivatives **3a&b**, **4a–e** and **5a–e**.

Compd. No.	GPCR	Ion channel modulator	Kinase inhibitor	Nuclear receptor Ligand	Protease inhibitor
**3a**	0.14	−0.72	−0.32	−0.58	−0.56
**3b**	0.22	−0.56	−0.10	−0.33	−0.34
**4a**	−0.33	−1.06	−0.58	−0.80	−0.67
**4b**	−0.14	−0.94	−0.67	−0.60	−0.71
**4c**	−0.18	−0.79	−0.42	−0.49	−0.58
**4d**	−0.21	−0.82	−0.45	−0.51	−0.61
**4e**	−0.21	−0.80	−0.42	−0.47	−0.59
**5a**	−0.34	−1.15	−1.00	−0.81	−0.86
**5b**	−0.16	−0.90	−0.89	−0.70	−0.75
**5c**	−0.21	−0.87	−0.71	−0.55	−0.62
**5d**	−0.23	−0.90	−0.73	−0.56	−0.65
**5e**	−0.23	−0.87	−0.70	−0.53	−0.63

All tested derivatives were inactive as ion channel modulators and protease inhibitors. Compounds **3b**, **4c,** and **4e** bearing phenyl semicarbazone/thiosemicarbazone or *p*-methoxyphenylthiosemicarbazone scaffolds were moderately active as kinase inhibitors and nuclear receptor ligand, in addition to semicarbazone derivative **3a** and *p*-tolylthiosemicarbazone derivative **4d** that might be considered as kinase inhibitors.

### In silico ADME prediction

Pharmacokinetic properties (absorption, distribution, metabolism and excretion) for the synthesised compounds were predicted using in silico ADME prediction.

The obtained results were recorded in Table 9. All the target compounds showed high intestinal absorption with values ranging from 93.40 to 99.66% and reached 100% in the case of thiazolidinone derivative **5e**. Low to moderate permeability results were observed for *in vitro* CaCo-2 and MDCK cells in the range 0.58–24.16 and 0.04–19.81 nm/sec, respectively. Most of the compounds exerted a strong binding effect on plasma proteins higher than 90% except semicarbazone derivative **3a** and thiosemicarbazone derivatives **4a** and **4b**. They possessed plasma binding values of 57.14, 79.56, and 86.83%, sequentially.

**Table 9.** ADME prediction for the synthesised indole derivatives **3a&b**, **4a–e** and **5a–e**.

Compd. No.	HIA (%)	*In vitro* Caco-2 cell permeability (nm/sec)	*In vitro* MDCK cell permeability (nm/sec)	BPB (%)	*In vivo* BBB (C.brain/C.blood)	logKp (cm/hour)
**3a**	93.40	0.58	19.81	57.14	0.21	−2.88
**3b**	94.86	0.91	0.24	100.00	0.01	−2.43
**4a**	96.53	6.33	9.07	79.56	0.29	−2.64
**4b**	95.41	8.66	4.46	86.83	0.01	−2.43
**4c**	95.76	19.17	0.07	100.00	0.03	−2.17
**4d**	95.88	24.16	0.05	100.00	0.06	−2.10
**4e**	95.66	15.95	0.06	100.00	0.02	−2.33
**5a**	97.48	0.88	1.02	100.00	0.03	−2.53
**5b**	99.60	8.21	0.05	98.52	0.06	−2.03
**5c**	99.66	15.47	0.04	100.00	0.09	−1.79
**5d**	99.50	18.63	0.04	100.00	0.09	−1.74
**5e**	100.00	15.79	0.04	100.00	0.10	−1.84

Moreover, all the synthesised compounds exerted low absorption into the CNS. They couldn’t penetrate it. They had values in the range of 0.01–0.21 (i.e. <0.40, the standard value). Consequently, all the synthesised targets might be a good lead for the transdermal delivery systems. They showed maximum skin permeability with logKp values ranging from −2.88 to −1.74 cm/h.

### Metabolism prediction

Metabolism prediction was obtained by studying phase I metabolism parameters. Data in Table 10 shows that most of the compounds could inhibit the cytochrome P450 isoform CYP-2C9. While none of them could inhibit other cytochrome isoforms such as CYP-2C19, CYP-2D6 and CYP-3A4.

**Table 10.** Metabolism prediction for the synthesised indole derivatives **3a&b**, **4a-e** and **5a-e**.

Compd. No.	CYP-2C19 inhibitor	CYP-2C9 inhibitor	CYP-2D6 inhibitor	CYP-3A4 inhibitor	Compd. No.	CYP-2C19 inhibitor	CYP-2C9 inhibitor	CYP-2D6 inhibitor	CYP-3A4 inhibitor
**3a**	No	No	No	No	**4e**	No	Yes	No	No
**3b**	No	Yes	No	No	**5a**	No	No	No	No
**4a**	No	No	No	No	**5b**	No	No	No	No
**4b**	No	No	No	No	**5c**	No	Yes	No	No
**4c**	No	Yes	No	No	**5d**	No	Yes	No	No
**4d**	No	Yes	No	No	**5e**	No	Yes	No	No

## Conclusion

**Figure 12.**
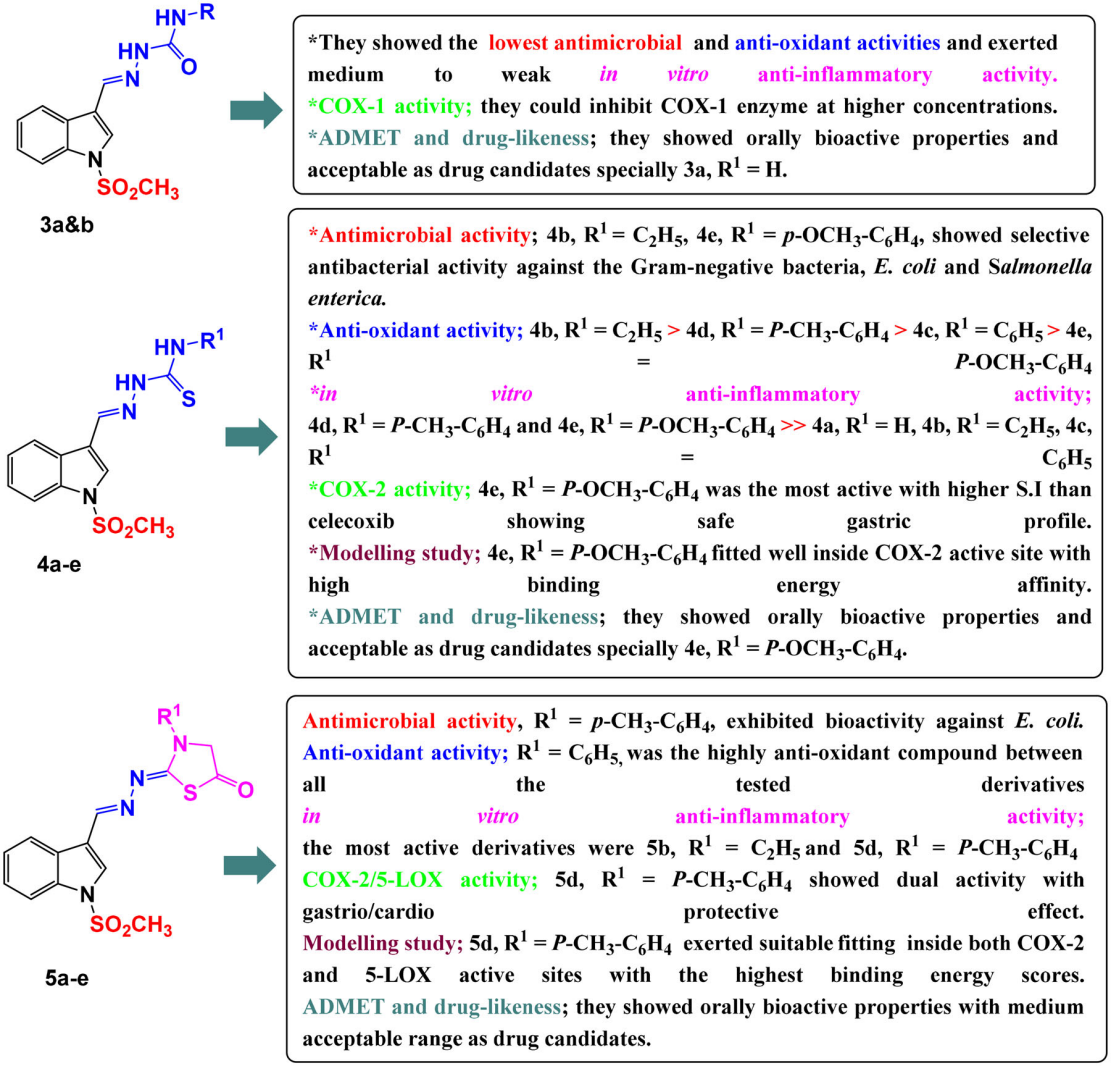
SAR for the synthesised indole derivatives **3a&b**, **4a–e** and **5a–e**.

A novel series of *N*-methylsulfonyl indole derivatives fused with semicarbazone **3a&b**, thiosemicarbazone **4a–e,** and thiazolidinone **5a–e** scaffolds were synthesised and evaluated for their biological activities. The antimicrobial screening revealed that thiosemicarbazide derivatives, **4b** and **4e** showed selective antibacterial activity against the Gram-negative bacteria, *E. coli* and *Salmonella enterica*. Whereas thiazolidinone derivative **5d** exhibited bioactivity against *E. coli* only. On the other hand, the tested compounds did not display antifungal activity. The *in vitro* antioxidant activity of the target compounds showed that phenylthiazolidinone derivative **5c** had the highest anti-oxidant activity among all the tested derivatives with a value of 707.5 µM Trolox equivalent/mg. For most thiosemicarbazide derivatives, their anti-oxidant activity was higher than their thiazolidinone analogs. The lowest scavenging activity on DPPH was observed in semicarbazone derivatives **3a** and **3b**, unsubstituted thiosemicarbazide derivative **4a**, its thiazolidinone analog **5a** and *p*-methoxyphenylthiazolidinone derivative **5e**. The *in vitro* anti-inflammatory results by measuring TNF-α in RAW264.7 macrophage cells revealed that compounds **4d**, **4e**, **5b** and **5d** had the highest anti-inflammatory activity, while, phenyl semicarbazone derivative, **3b** and thiazolidinone derivative, **5a** exerted the lowest activity. Furthermore, the synthesised compounds were tested against COX-1, COX-2 and 5-LOX enzymes. Compound **4e** could be classified as a selective COX-2 inhibitor and compound **5d** had dual COX-2/5-LOX inhibitory activity. As a result, measuring cardiac biomarkers (LDH, CK-MB and Tn-I) for compound **5d** elicited low levels. A histopathological study affirmed the cardioprotective profile for the target compound **5d**. Additionally, for both derivatives, **4e** and **5d**, ulcerogenic liability and the histopathological study confirmed their safety effect on the gastric mucosa. Moreover, a docking study for the target compounds inside COX-2 and 5-LOX active sites was performed to explain their plausible binding mode. Finally, an ADMET study and pharmacokinetic properties were applied and proved the promising activity of the new compounds. In conclusion, the newly developed compounds represent biologically active multi-target candidates with fewer side effects. Structure-activity relationship (SAR) is represented in the following Figure 12.

## Supplementary Material

Supplemental Material

## Data Availability

The data supporting the findings of this study are available within the article [and/or] its supplementary materials.
