# Supplementary material for: Novel N-methylsulfonyl-indole derivatives: biological activity and COX-2/5-LOX inhibitory effect with improved gastro protective profile and reduced cardio vascular risks
Source: J Enzyme Inhib Med Chem. 2022 Dec 1;38(1):246–66. doi: 10.1080/14756366.2022.2145283 (PMC9721424; doi:10.1080/14756366.2022.2145283)
Supplement: Supplemental Material [file IENZ_A_2145283_SM4212.zip › IENZ_2145283_SuppMat1.pdf]

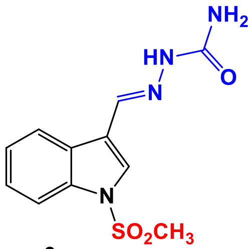

3a

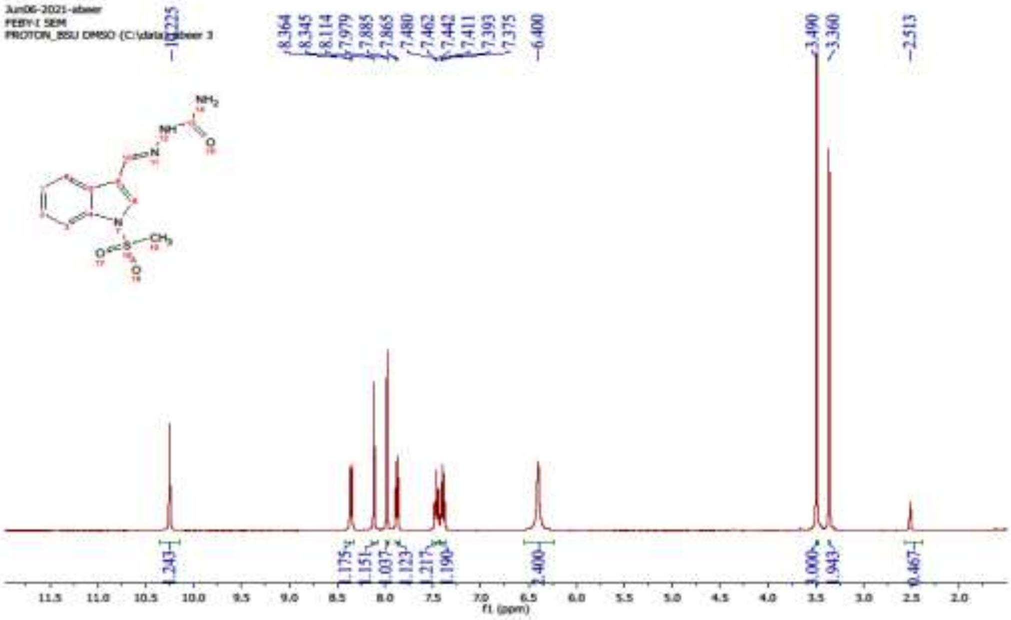

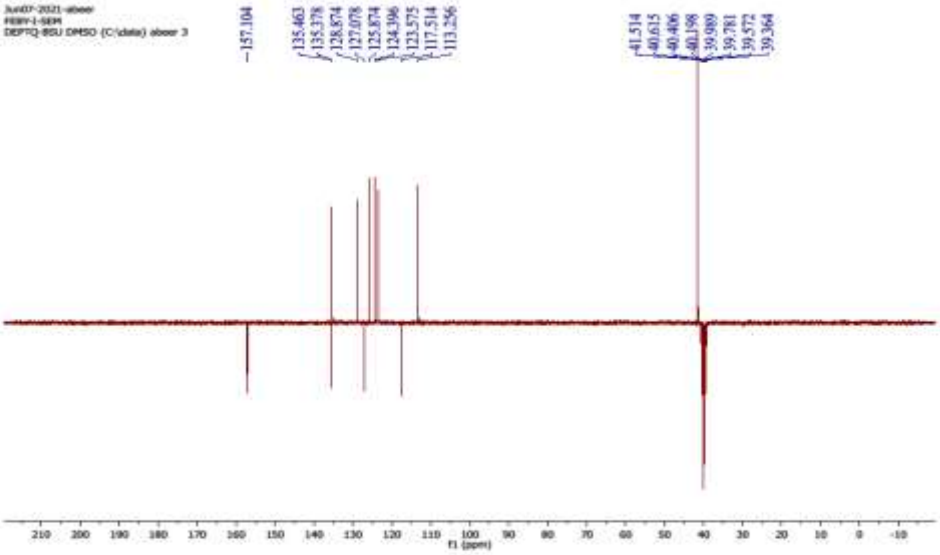

Information Classification: General

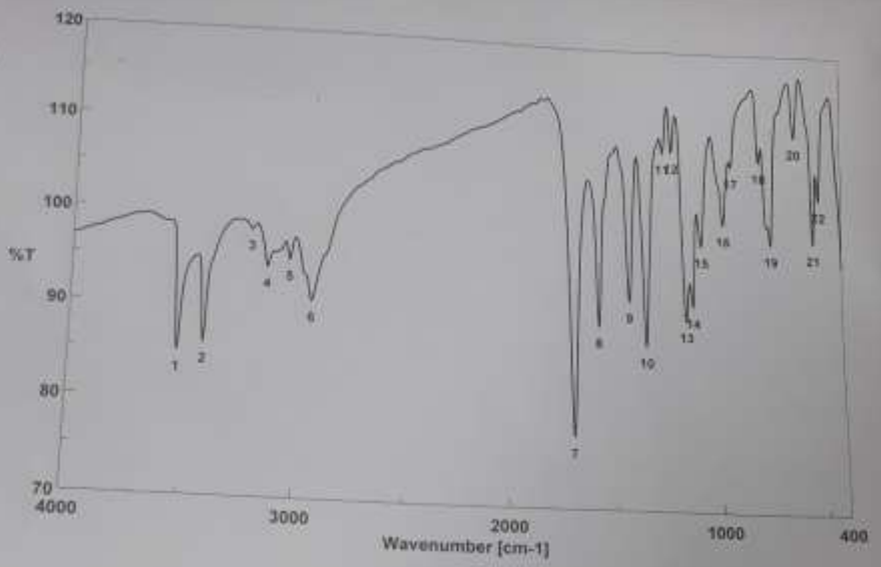

Accumulation	16
Resolution	4 cm-1
Zero Filling	ON
Apodization	Cosine
Gain	Auto (2)
Scanning Speed	Auto (2 mm/sec)
Date/Time	9/26/2021 1:14PM
Update	9/26/2021 1:15PM
Operator	IR
File Name	Memory#74
Sample Name	3a
Comment	

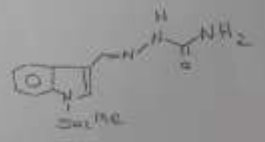

No.	cm-1	%T	No.	cm-1	%T	No.	cm-1	%T
1	3517.52	84.8818	2	3403.74	85.8423	3	3194.51	97.9868
4	3122.19	94.0494	5	3020.94	94.946	6	2920.66	90.6165
7	1696.09	77.5661	8	1581.34	89.3196	9	1436.71	92.1659
10	1356.68	87.3809	11	1269.9	108.817	12	1227.47	108.93
13	1104.79	90.3437	14	1131.05	91.8929	15	1087.66	98.6159
16	978.697	101.092	17	938.199	107.573	18	799.35	108.252
19	746.317	99.1137	20	626.752	111.098	21	537.078	99.4531
22	509.115	104.243						

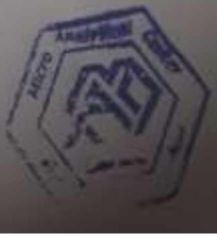

Information Classification: General

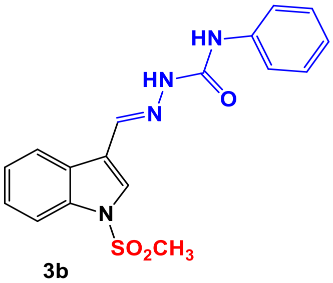
HN
HN
N
O
N
SO2CH3

3b

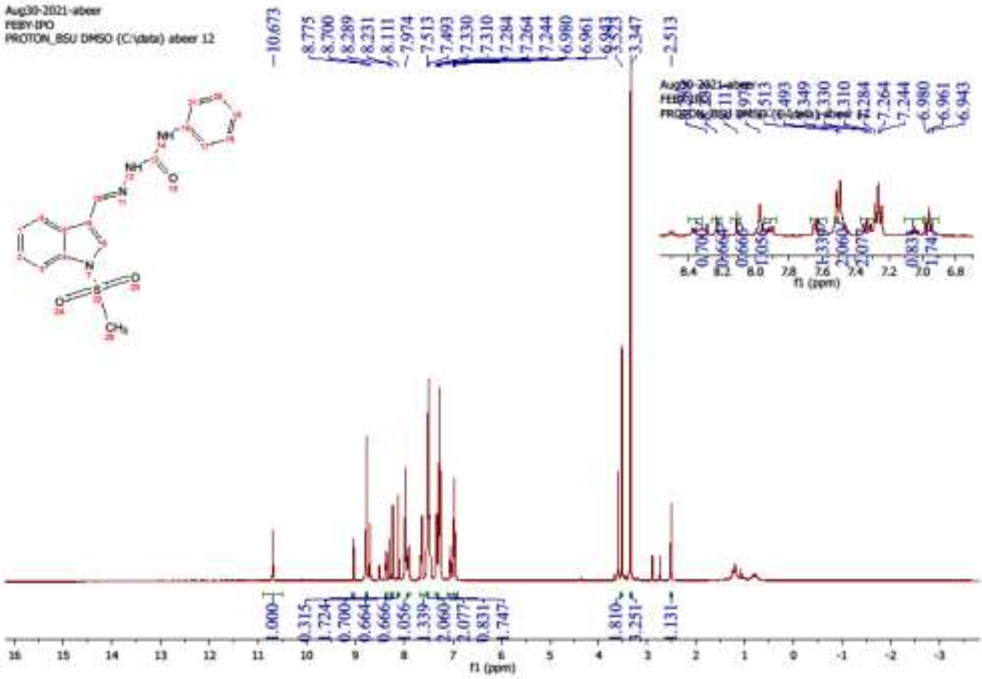
Aug30-2021-abeer
FEBY-IPO
PROTON_BSU DMSO {C:\data} abeer 12
f1 (ppm)

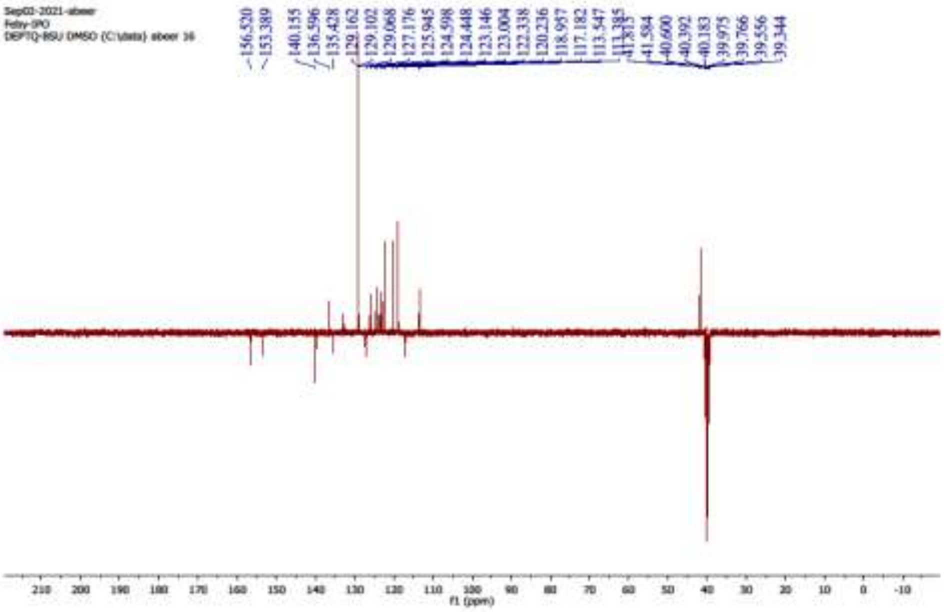
Sep02-2021-abeer
Feby-IPO
DEPTQ-BSU DMSO {C:\data} abeer 16
f1 (ppm)

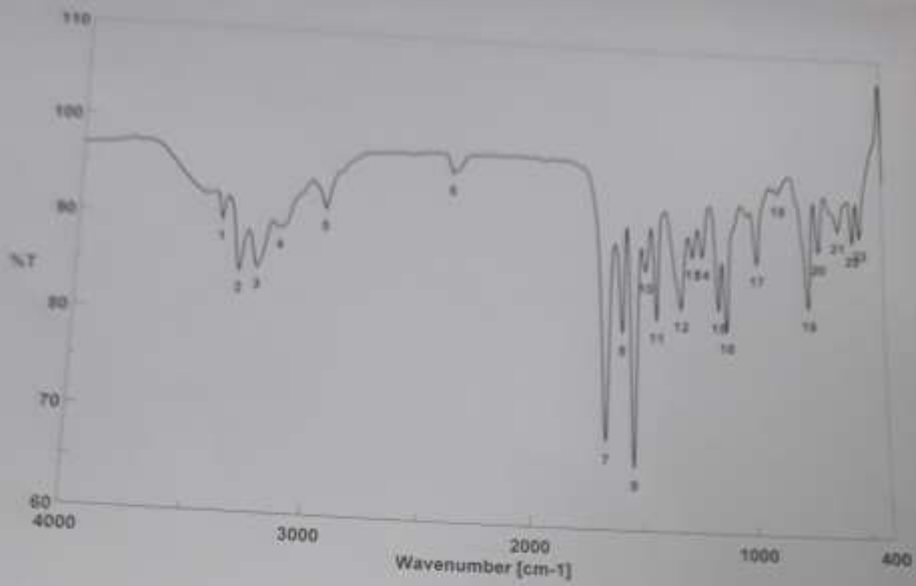

Accumulation	16
Resolution	4 cm-1
Zero Filling	ON
Apodization	Cosine
Gain	Auto (2)
Scanning Speed	Auto (2 mm/sec)
Date/Time	9/26/2021 1:08PM
Update	9/26/2021 1:09PM
Operator	IR
File Name	Memory#52
Sample Name	3b
Comment	

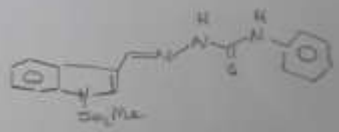

No.	cm-1	%T	No.	cm-1	%T	No.	cm-1	%T
1	3375.78	89.4906	2	3296.71	84.2436	3	3218.61	84.6948
4	3123.15	88.9282	5	2925.48	91.084	6	2360.44	95.5155
7	1672.95	68.7425	8	1599.66	79.8324	9	1547.59	66.2544
10	1494.56	86.3361	11	1446.35	81.2455	12	1336.43	82.5529
13	1282.43	88.0284	14	1238.08	88.1228	15	1165.76	82.5767
16	1129.12	80.5306	17	987.375	87.6896	18	886.131	95.2899
19	753.066	83.297	20	700.998	89.3229	21	609.396	91.7173
22	541.899	90.4488	23	509.115	91.0476			

Information Classification: General

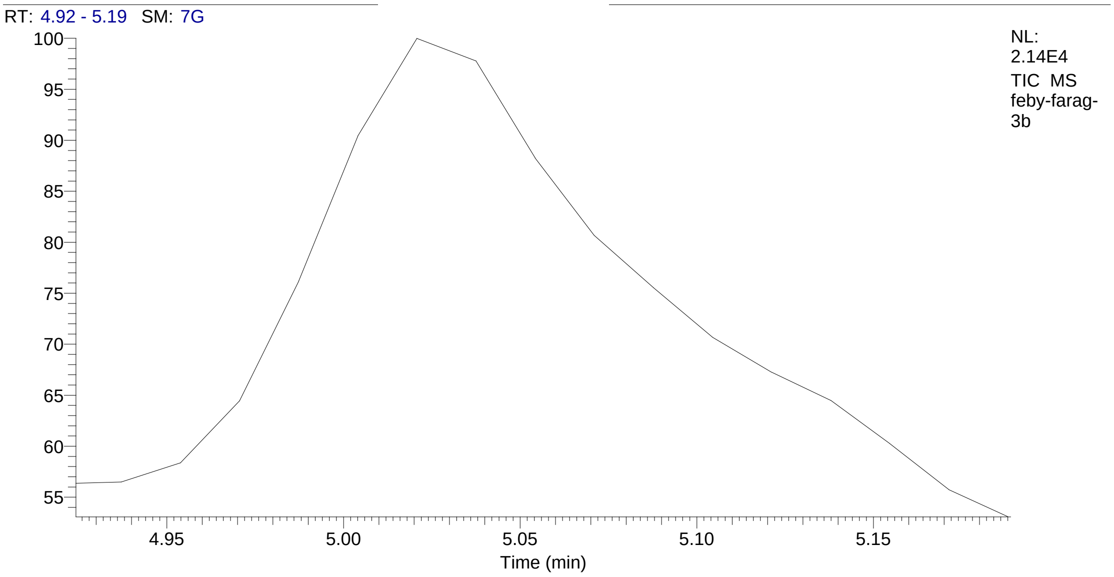

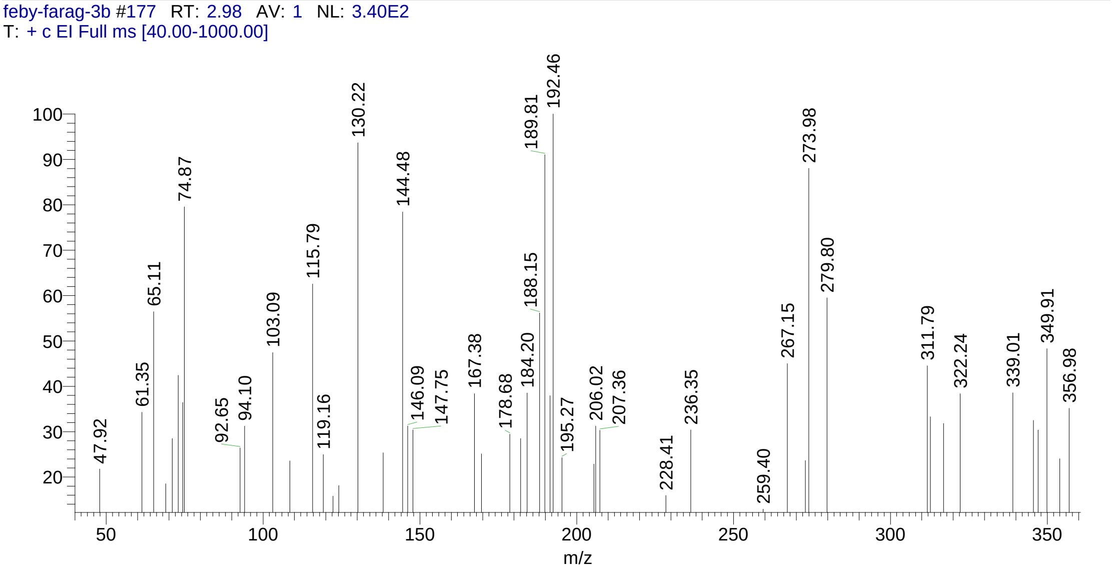

Information Classification: General

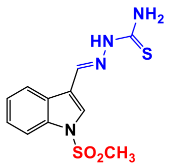

4a

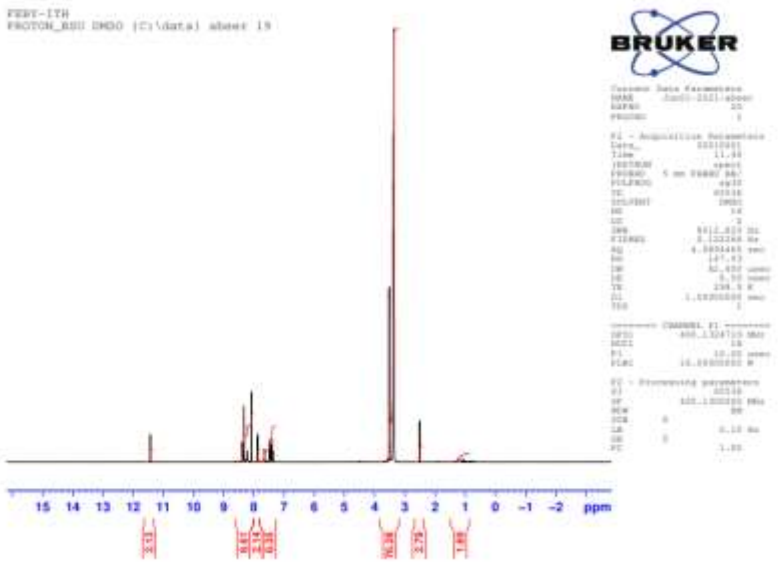

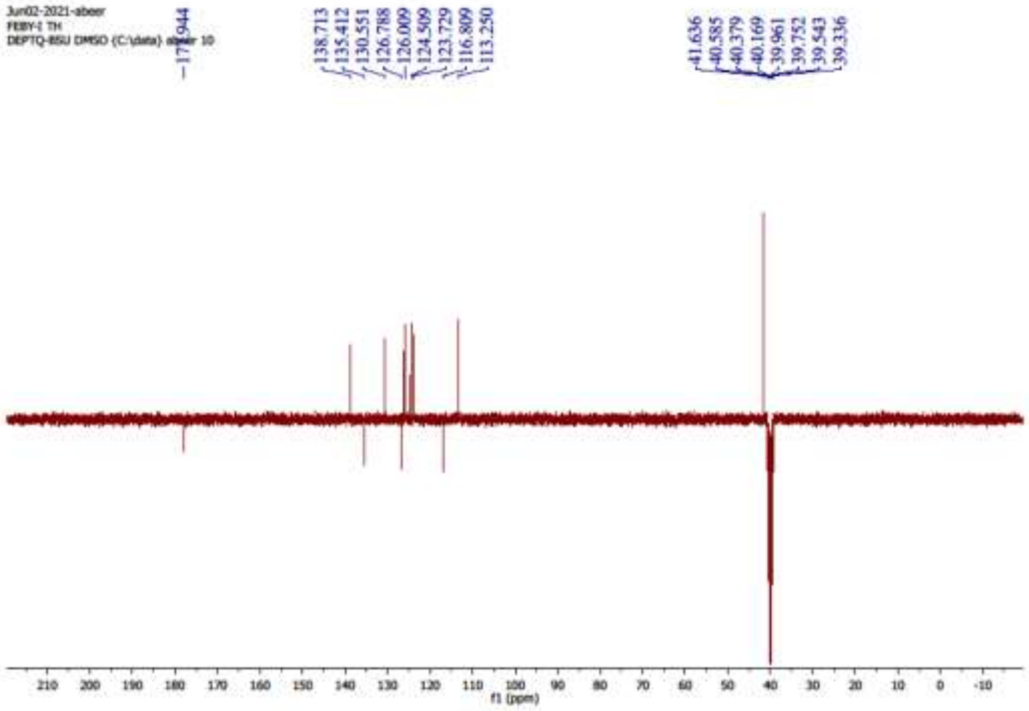

Information Classification: General

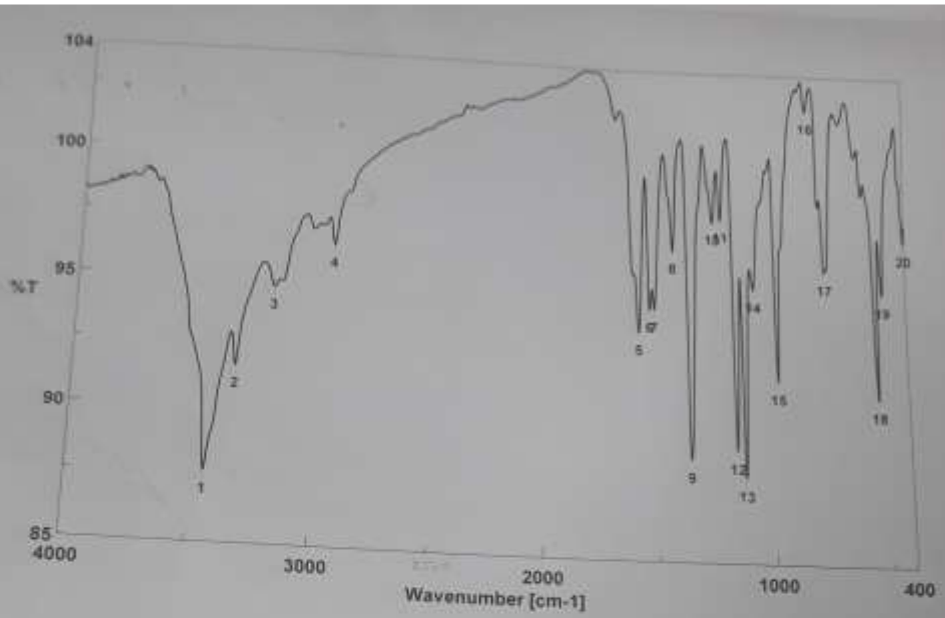

Accumulation	16
Resolution	4 cm-1
Zero Filling	ON
Apodization	Cosine
Gain	Auto (2)
Scanning Speed	Auto (2 mm/sec)
Date/Time	9/26/2021 1:15PM
Update	9/26/2021 1:16PM
Operator	IR
File Name	Memory#77
Sample Name	4a
Comment	

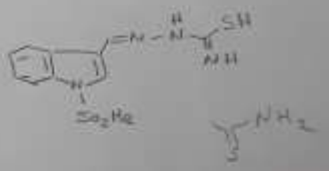

No.	cm-1	%T	No.	cm-1	%T	No.	cm-1	%T
1	3435.56	87.6044	2	3323.71	91.6079	3	3171.36	94.6752
4	2920.66	96.3914	5	1592.91	93.5783	6	1545.67	94.4576
7	1525.42	94.4757	8	1444.42	96.797	9	1364.39	88.8066
10	1267	97.9951	11	1230.36	98.117	12	1165.76	89.1839
13	1129.12	88.1743	14	1088.62	95.4175	15	983.518	91.869
16	840.812	102.586	17	765.601	96.218	18	537.078	91.3087
19	506.223	95.4268	20	403.05	97.5028			

Analytical Center

Information Classification: General

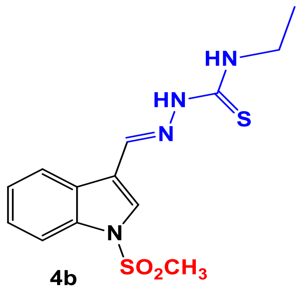

HN
HN
N
S
N
SO2CH3

4b

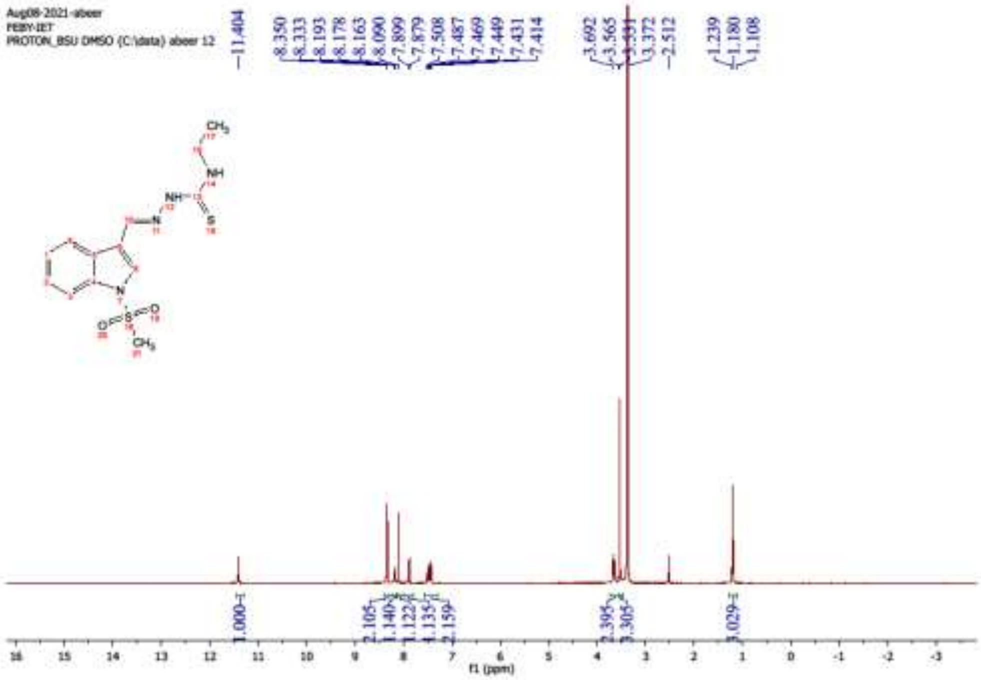

Aug08-2021-abeer
FEBY-IET
PROTON_BSU DMSO {C:\data} abeer 12
11.404
8.350
8.333
8.193
8.178
8.163
8.090
7.899
7.879
7.508
7.487
7.469
7.449
7.431
7.414
3.692
3.565
3.331
3.372
2.512
1.239
1.180
1.108
1.000
2.105
1.140
1.122
1.135
2.159
2.395
3.305
3.029
f1 (ppm)

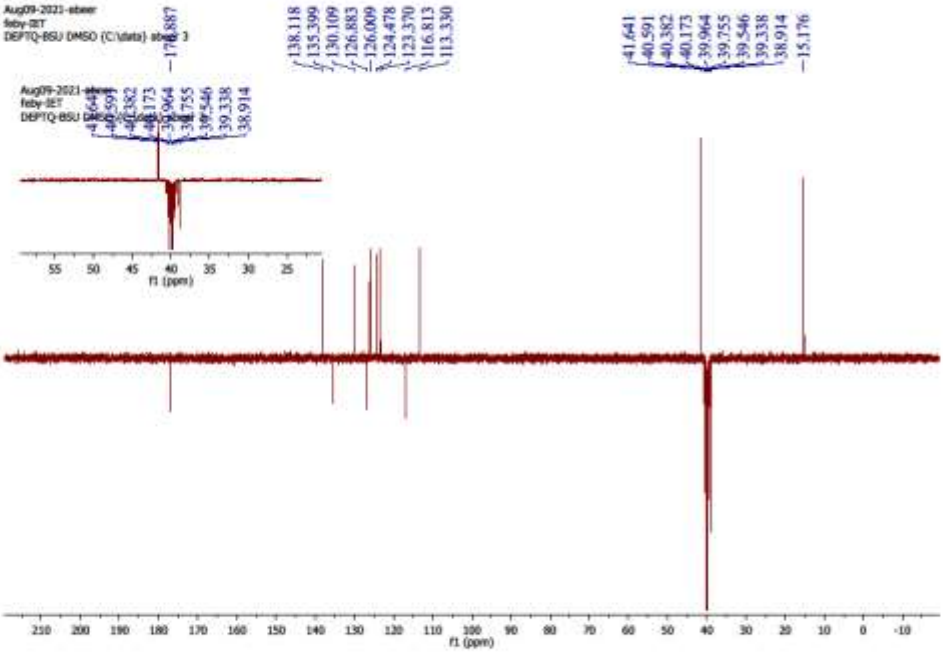

Aug09-2021-abeer
feby-IET
DEPTQ-BSU DMSO {C:\data} abeer 3
176.887
138.118
135.399
130.109
126.883
126.009
124.478
123.370
116.813
113.330
41.641
40.591
40.382
40.173
39.964
39.755
39.546
39.338
38.914
15.176
f1 (ppm)

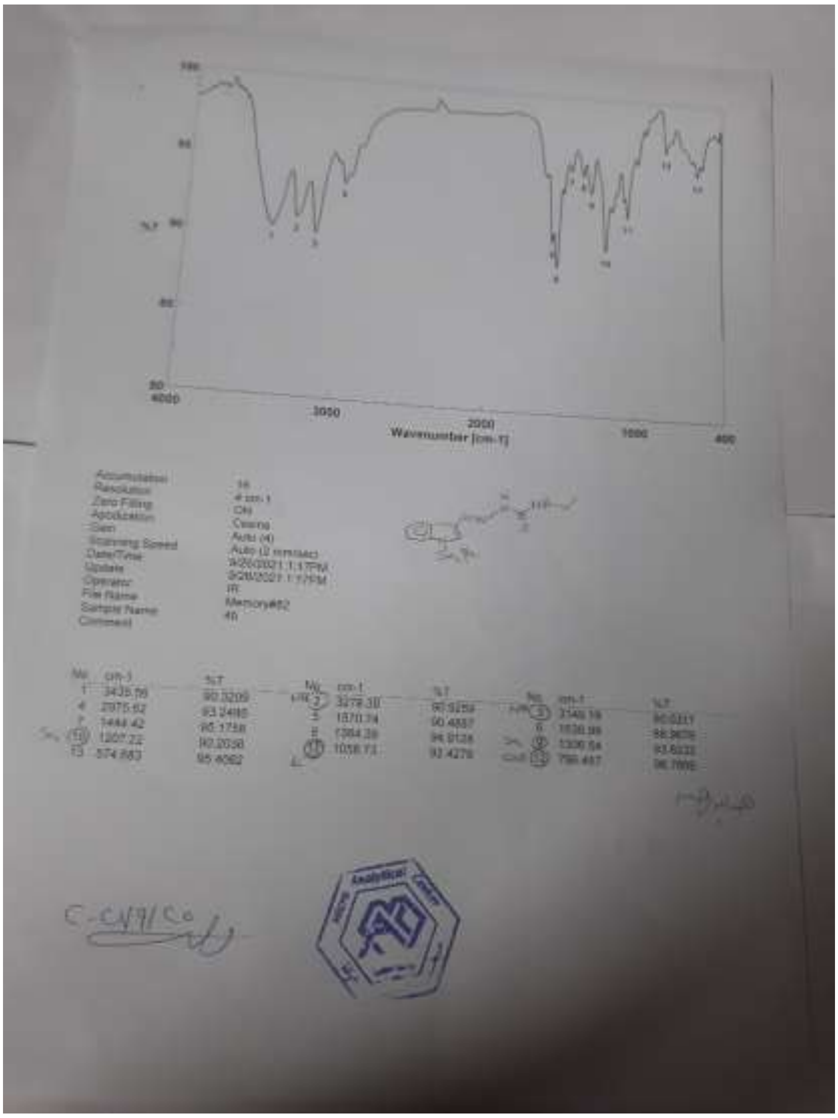

Parameter	Value
Accumulation	16
Resolution	4 cm-1
Zero Filling	ON
Apodization	Cosine
Gain	Auto (4)
Scanning Speed	Auto (2 mm/sec)
Date/Time	3/26/2021 1:17PM
Update	3/26/2021 1:17PM
Operator	IR
File Name	Memory#82
Sample Name	46
Comment	

No.	cm-1	%T	No.	cm-1	%T	No.	cm-1	%T
1	3435.56	90.3209	2	3278.30	90.9259	3	3146.19	[illegible]
4	2975.62	93.2486	5	1570.74	90.4887	6	1530.99	[illegible]
7	1444.42	95.1758	8	1384.39	94.9128	9	1336.54	[illegible]
10	1207.22	90.2036	11	1056.73	92.4276	12	756.487	[illegible]
13	574.683	95.4062						

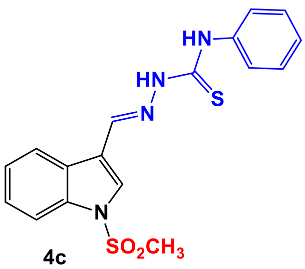

4c

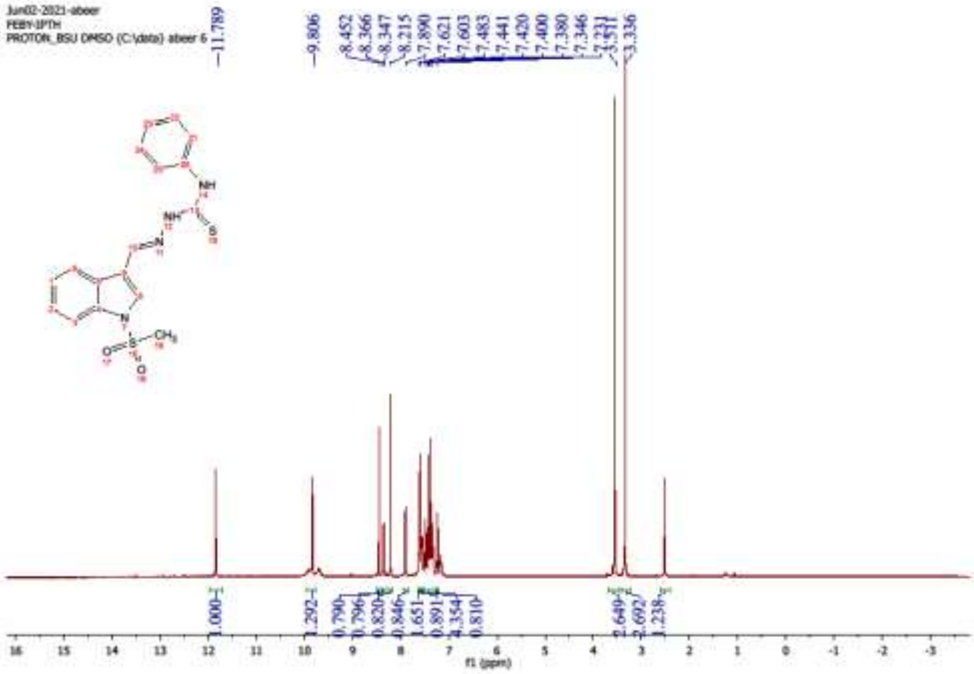

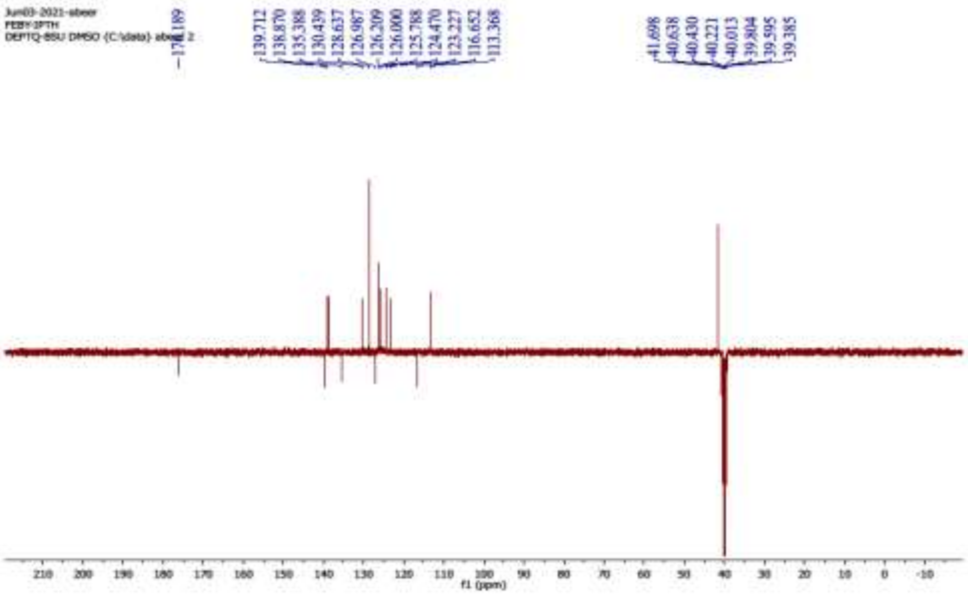

Information Classification: General

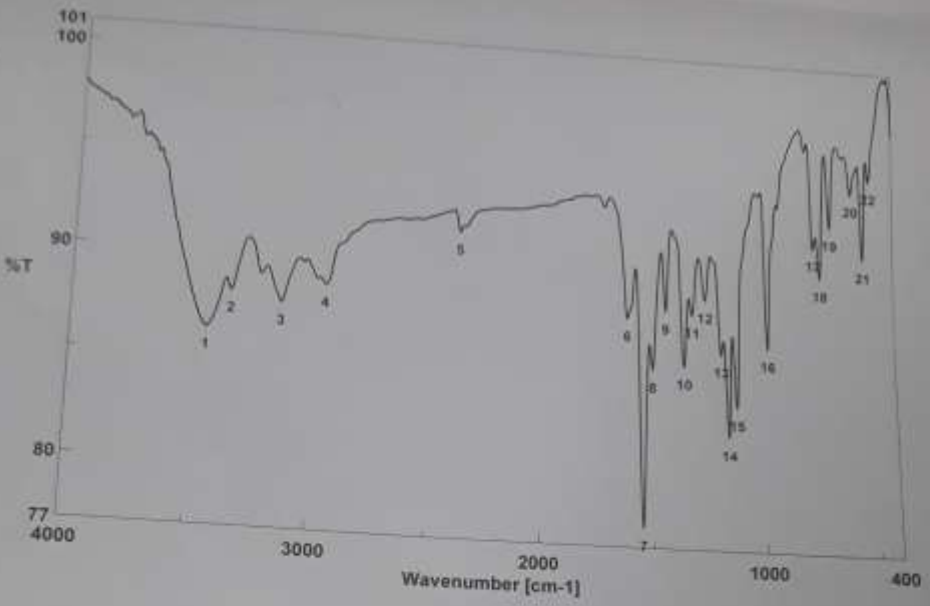

Accumulation	16
Resolution	4 cm-1
Zero Filling	ON
Apodization	Cosine
Gain	Auto (2)
Scanning Speed	Auto (2 mm/sec)
Date/Time	9/26/2021 1:11PM
Update	9/26/2021 1:12PM
Operator	IR
File Name	Memory#65
Sample Name	4C
Comment	

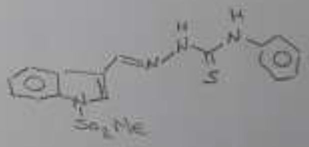

No.	cm-1	%T	No.	cm-1	%T	No.	cm-1	%T
1	3433.64	86.2549	2	3333.36	88.0625	3	3122.19	87.6327
4	2935.13	88.6206	5	2354.66	91.5773	6	1615.09	87.907
7	1545.67	77.9682	8	1503.24	85.482	9	1445.39	88.3592
10	1363.43	85.7141	11	1326.79	88.2438	12	1269.9	89.0136
13	1197.58	86.3917	14	1165.76	82.4065	15	1129.12	83.8575
16	987.375	86.8008	17	771.387	91.953	18	741.496	90.4799
19	693.284	93.0415	20	593.968	94.8265	21	539.971	91.5527
22	509.115	95.5347						

Micro Analytical Center

Information Classification: General

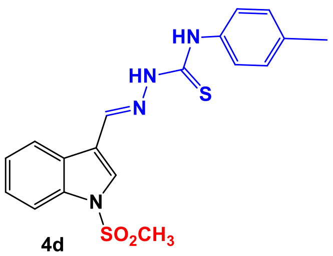
HN
HN
N
S
N
SO_2CH_3

4d

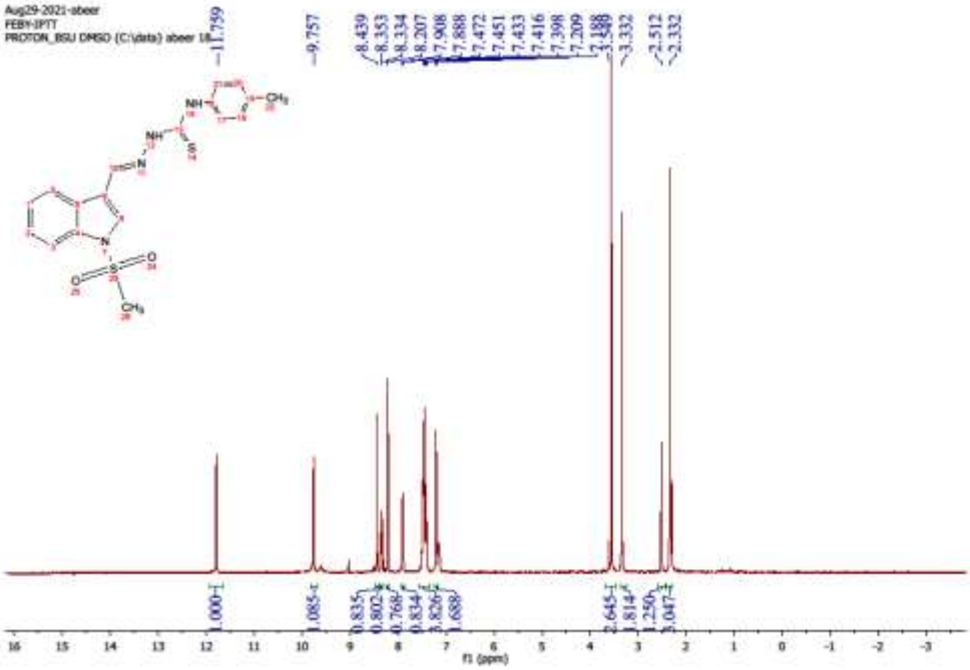
Aug29-2021-abeer
FEBY-IPTT
PROTON_BSU DMSO {C:\data} abeer 18
11.759
9.757
8.439
8.353
8.334
8.207
7.908
7.888
7.472
7.451
7.433
7.416
7.398
7.209
7.188
3.5…
[illegible]
3.332
2.512
2.332
1.000
1.085
0.835
0.802
0.768
0.834
3.826
1.688
2.645
1.814
1.250
3.047
f1 (ppm)

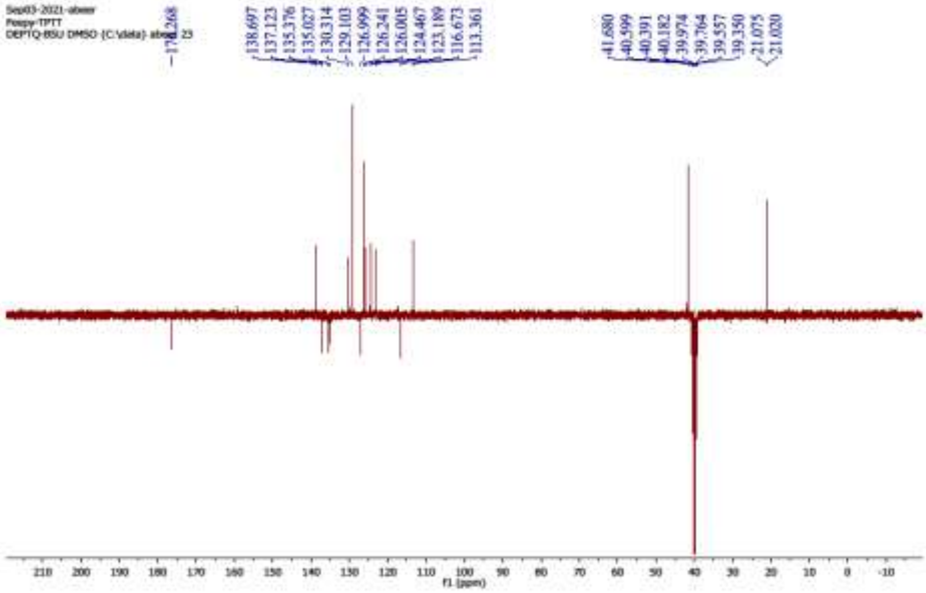
Sep03-2021-abeer
Feepy-TPTT
DEPTQ-BSU DMSO {C:\data} abee[illegible] 23
176.268
138.697
137.123
135.376
135.027
130.314
129.103
126.999
126.241
126.005
124.467
123.189
116.673
113.361
41.680
40.599
40.391
40.182
39.974
39.764
39.557
39.350
21.075
21.020
f1 (ppm)

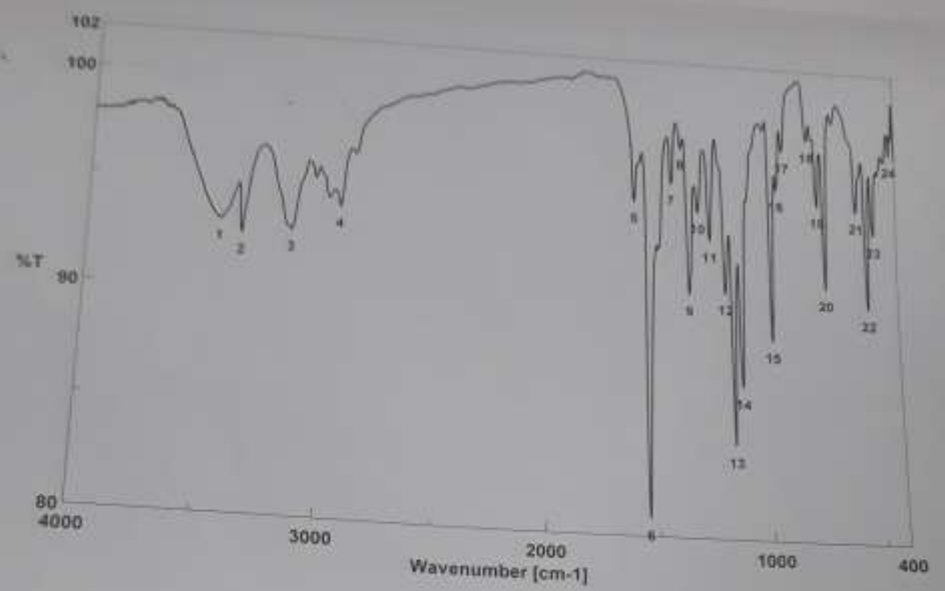

Accumulation	16
Resolution	4 cm-1
Zero Filling	ON
Apodization	Cosine
Gain	Auto (2)
Scanning Speed	Auto (2 mm/sec)
Date/Time	9/26/2021 1:13PM
Update	9/26/2021 1:13PM
Operator	IR
File Name	Memory#69
Sample Name	4d
Comment	

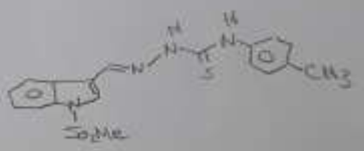

No	cm-1	%T	No.	cm-1	%T	No	cm-1	%T
1	3435.56	93.1149	2	3341.07	92.5266	3	3128.94	92.8076
4	2919.7	94.0263	5	1613.16	95.2284	6	1543.74	80.8289
7	1445.39	96.177	8	1402	97.9163	9	1364.39	91.029
10	1324.86	94.9019	11	1271.82	93.5752	12	1200.47	91.1675
13	1162.87	84.2065	14	1127.19	86.8607	15	988.339	89.0648
16	961.341	96.2223	17	933.378	98.0755	18	815.742	98.6721
19	770.423	95.5644	20	736.674	91.6567	21	588.182	95.4065
22	539.971	90.8244	23	508.151	94.3358	24	427.155	98.2259

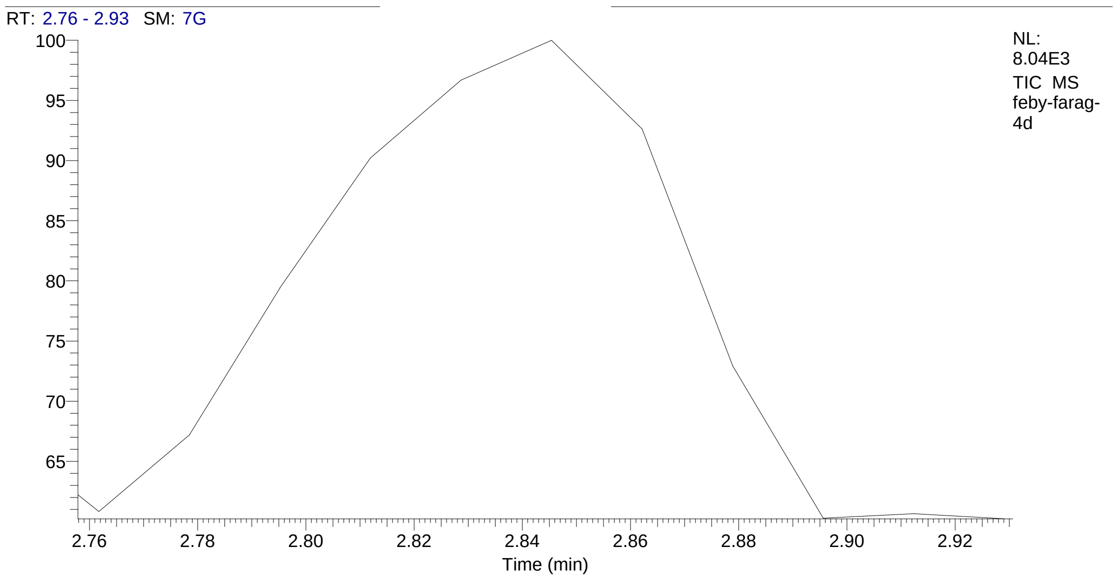

RT: 2.76 - 2.93 SM: 7G
NL: 8.04E3
TIC MS feby-farag-4d
100
95
90
85
80
75
70
65
2.76
2.78
2.80
2.82
2.84
2.86
2.88
2.90
2.92
Time (min)

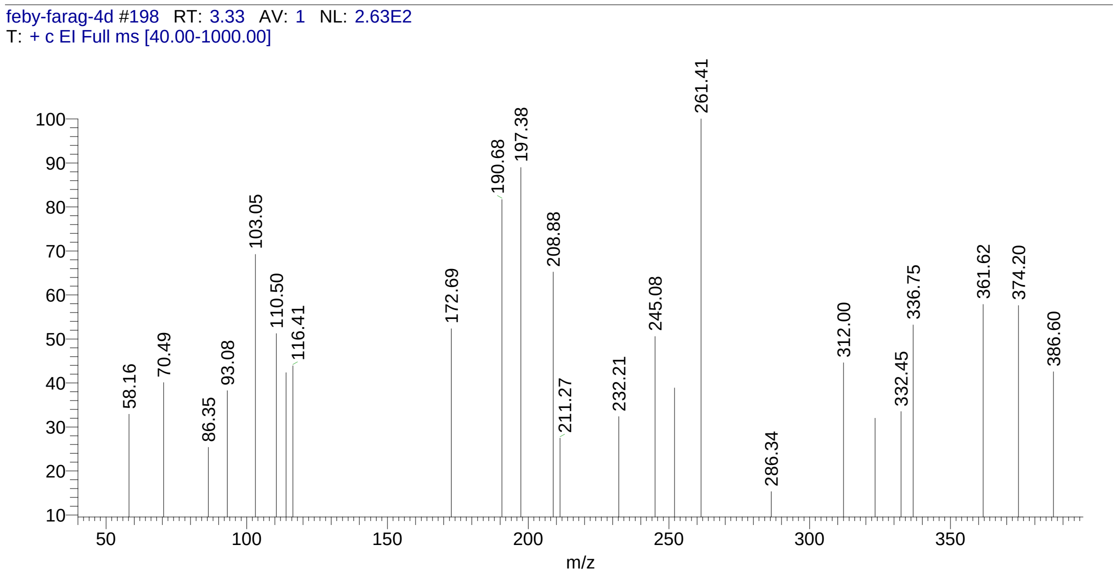

feby-farag-4d #198 RT: 3.33 AV: 1 NL: 2.63E2
T: + c EI Full ms [40.00-1000.00]
58.16
70.49
86.35
93.08
103.05
110.50
116.41
172.69
190.68
197.38
208.88
211.27
232.21
245.08
261.41
286.34
312.00
332.45
336.75
361.62
374.20
386.60
100
90
80
70
60
50
40
30
20
10
50
100
150
200
250
300
350
m/z

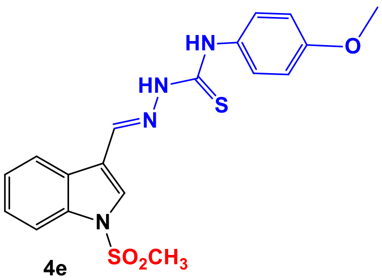
HN
O
HN
N
S
N
SO2CH3

4e

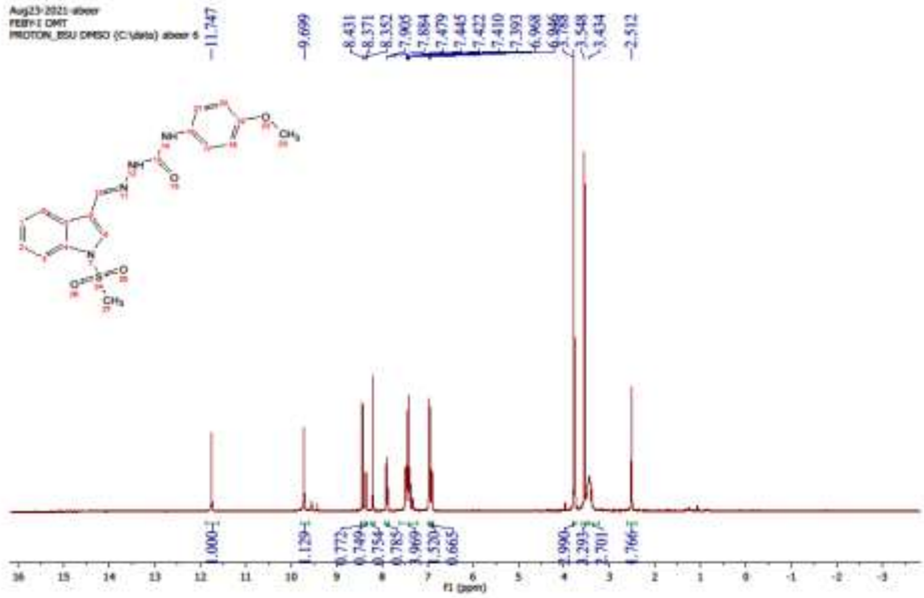
Aug23-2021-abeer
FEBY-I OMT
PROTON_BSU DMSO {C:\data} abeer 6
11.747
9.699
8.431
8.371
8.352
7.905
7.884
7.479
7.445
7.422
7.410
7.393
6.968
3.788
3.548
3.434
2.512
1.000
1.129
0.772
0.749
0.754
0.785
3.969
1.520
0.665
2.990
3.293
2.701
1.766
f1 (ppm)

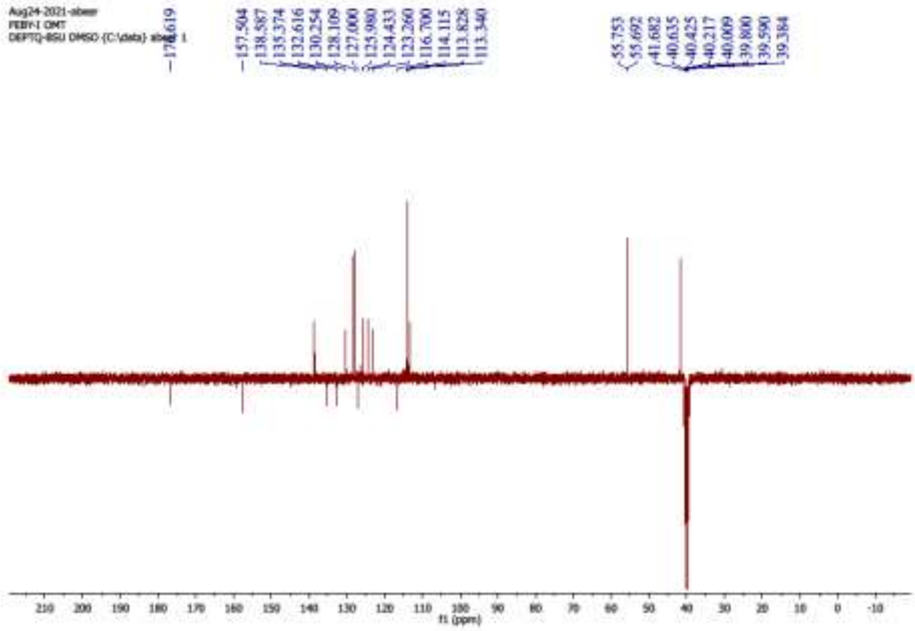
Aug24-2021-abeer
FEBY-I OMT
DEPTQ-BSU DMSO {C:\data} abeer 1
176.619
157.504
138.587
135.374
132.616
130.254
128.109
127.000
125.980
124.433
123.260
116.700
114.115
113.828
113.340
55.753
55.692
41.682
40.635
40.425
40.217
40.009
39.800
39.590
39.384
f1 (ppm)

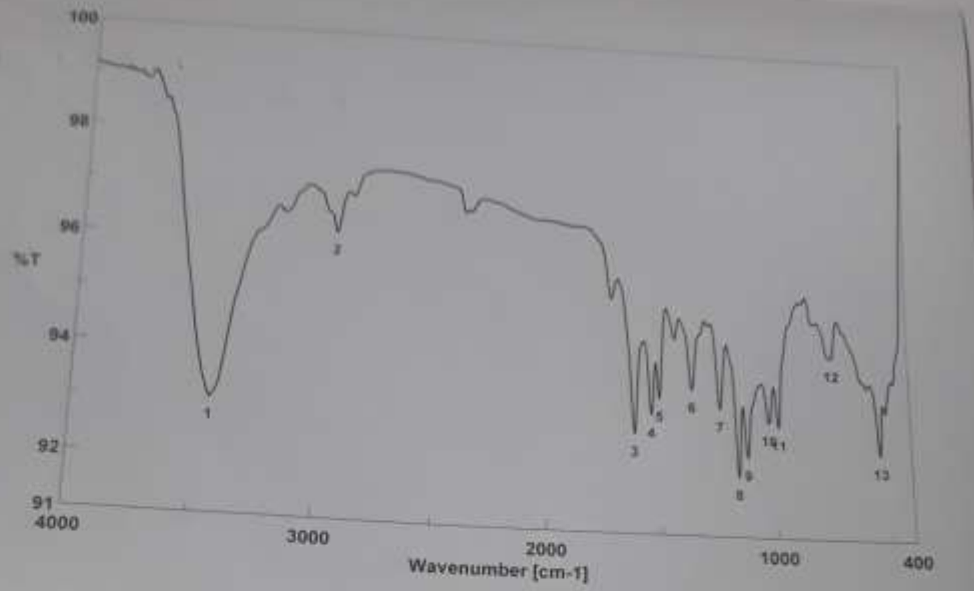

Accumulation	16
Resolution	4 cm-1
Zero Filling	ON
Apodization	Cosine
Gain	Auto (2)
Scanning Speed	Auto (2 mm/sec)
Date/Time	9/26/2021 1:05PM
Update	9/26/2021 1:05PM
Operator	IR
File Name	Memory#40
Sample Name	4e
Comment	

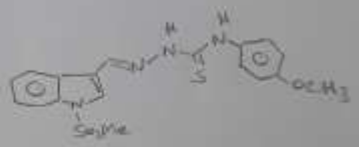

No	cm-1	%T	No.	cm-1	%T	No.	cm-1	%T
1	3431.71	93.0497	2	2923.56	96.1518	3	1620.88	92.7892
4	1546.63	93.1403	5	1511.92	93.436	6	1370.18	93.6226
7	1247.72	93.2896	8	1166.72	92.0754	9	1127.19	92.4321
10	1032.69	93.0707	11	989.304	92.9957	12	748.245	94.3102
13	540.935	92.5474						

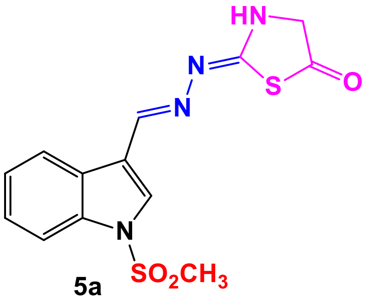

5a

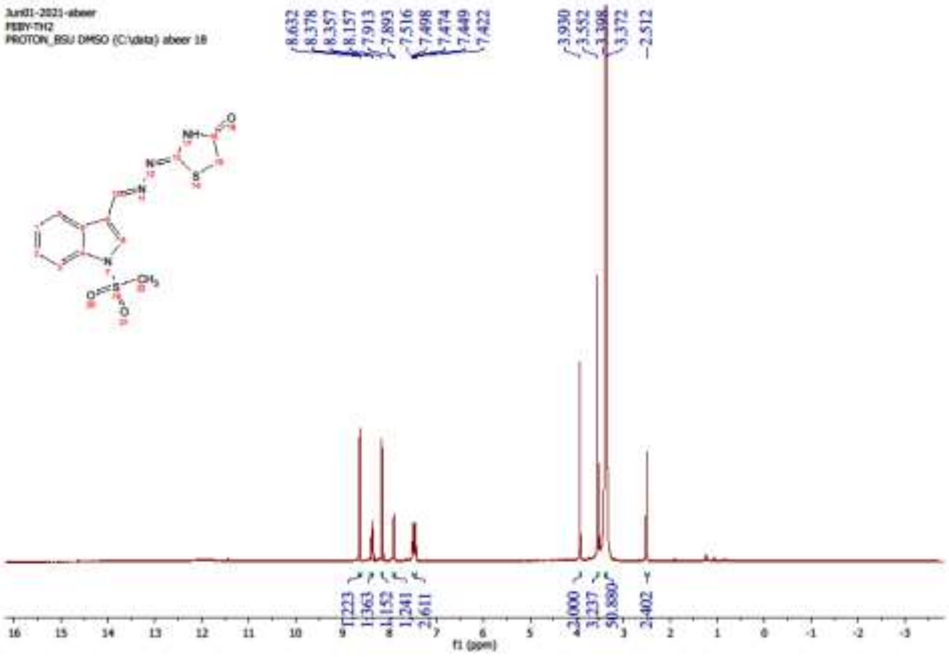

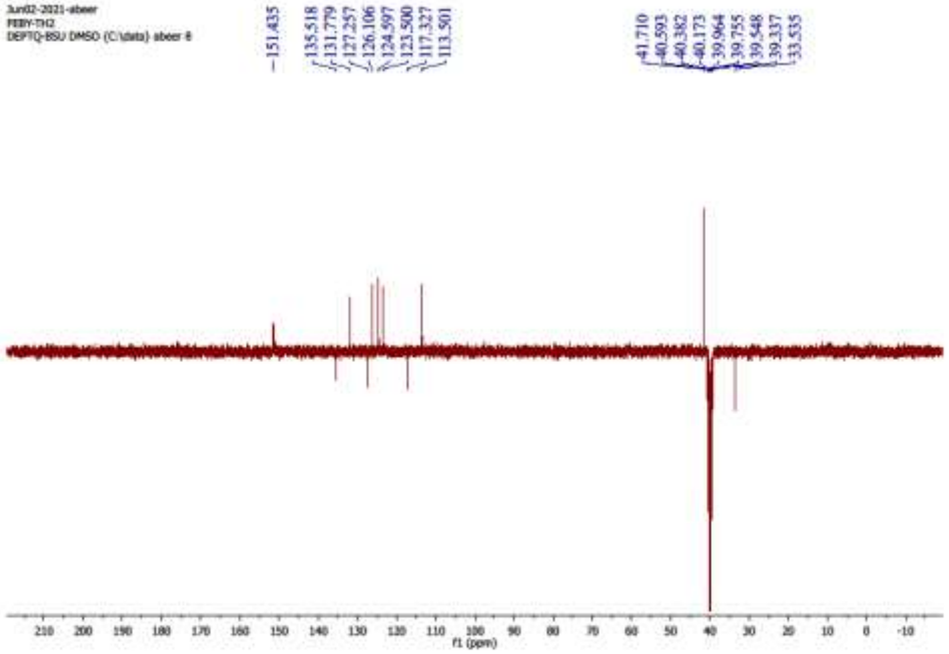

Information Classification: General

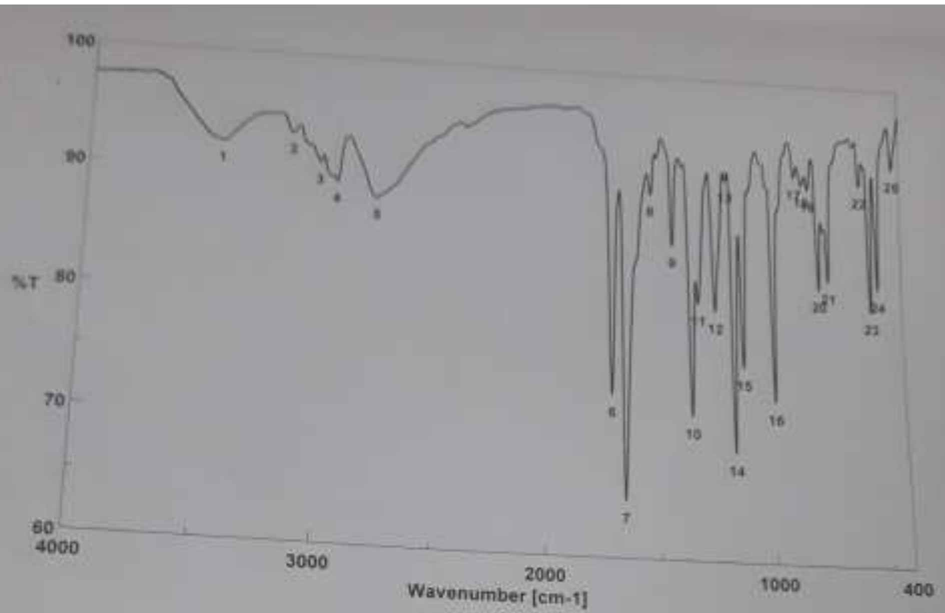

Accumulation	16
Resolution	4 cm-1
Zero Filling	ON
Apodization	Cosine
Gain	Auto (2)
Scanning Speed	Auto (2 mm/sec)
Date/Time	9/26/2021 1:06PM
Update	9/26/2021 1:06PM
Operator	IR
File Name	Memory#44
Sample Name	5a
Comment	

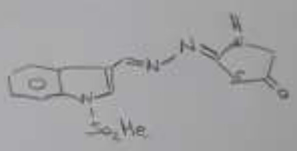

No.	cm-1	%T	No.	cm-1	%T	No.	cm-1	%T
1	3428.81	92.1153	2	3125.08	93.1629	3	3004.55	90.7274
4	2928.38	89.1991	5	2752.89	88.0476	6	1712.46	72.9976
7	1652.7	64.6243	8	1541.81	89.837	9	1446.35	85.6089
10	1360.53	71.558	11	1330.64	80.8594	12	1255.43	80.2993
13	1205.29	91.4732	14	1171.54	68.733	15	1131.05	75.7103
16	993.16	73.0205	17	889.023	92.1203	18	855.275	91.4981
19	826.348	91.1501	20	781.993	82.6631	21	738.603	83.4125
22	587.218	91.7258	23	543.828	81.0859	24	510.08	82.9583
25	435.834	93.2816						

Information Classification: General

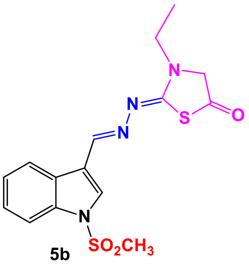

5b

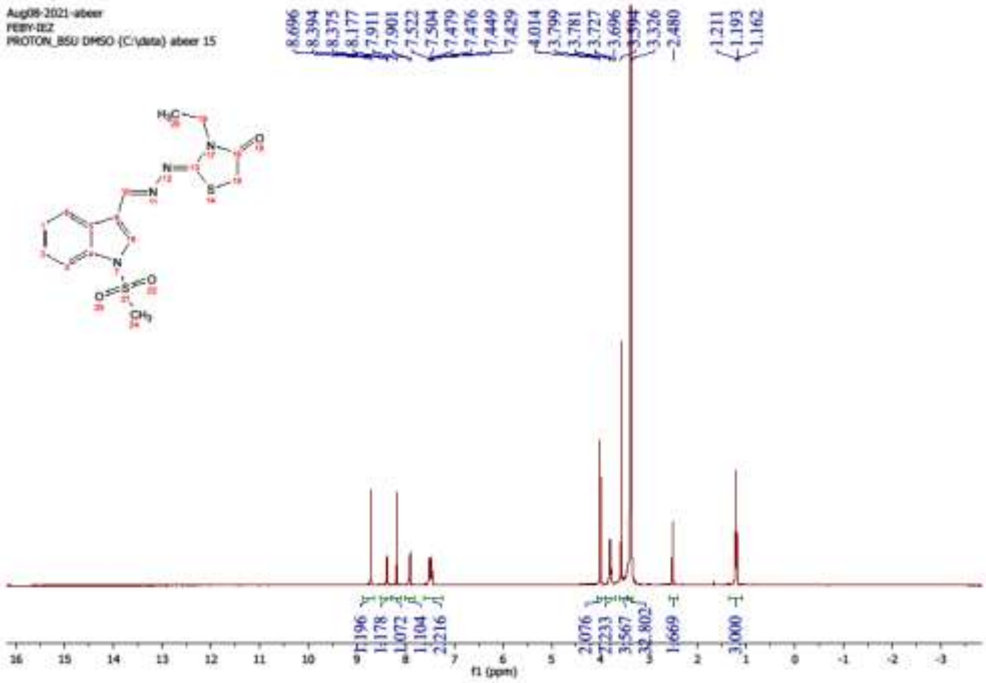

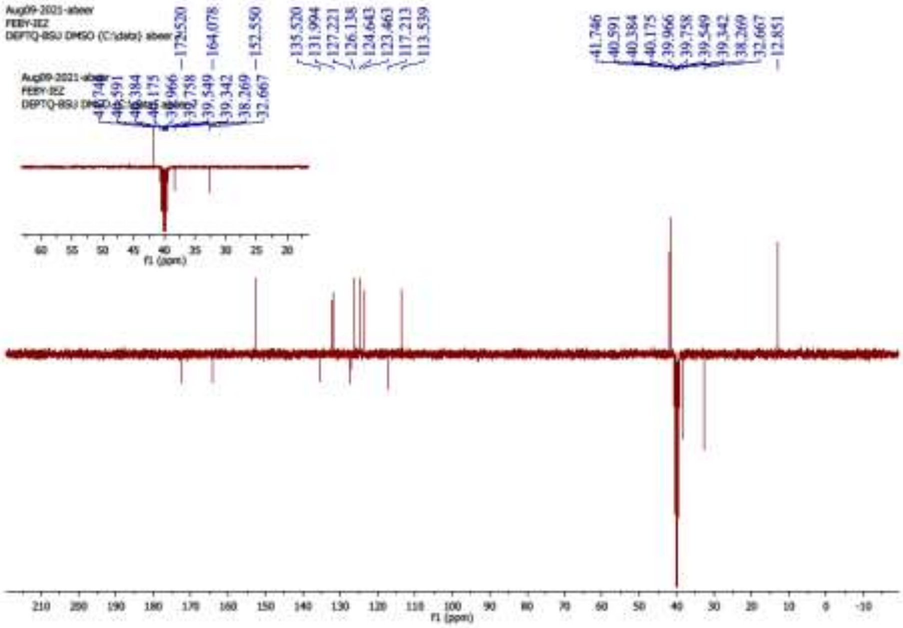

Information Classification: General

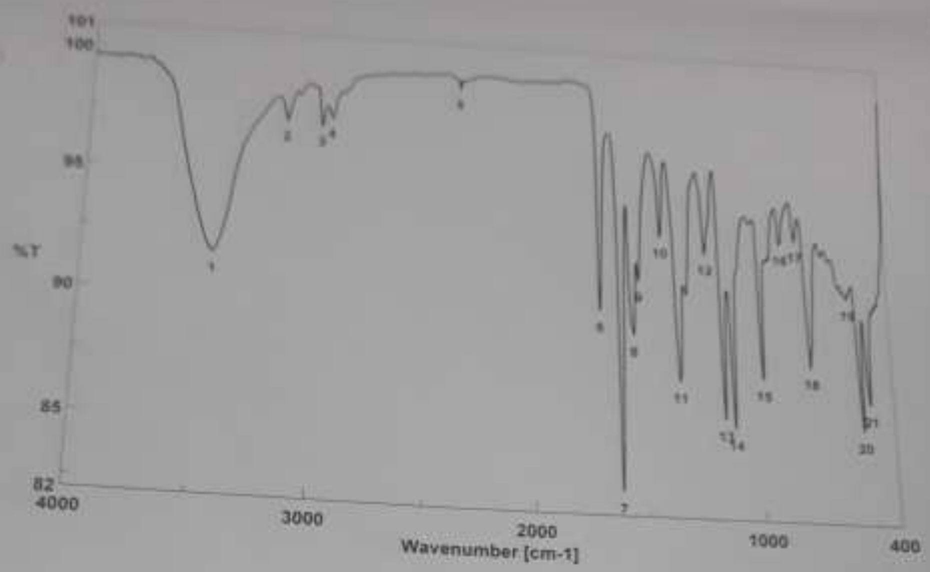

Accumulation	16
Resolution	4 cm-1
Zero Filling	ON
Apodization	Cosine
Gain	Auto (2)
Scanning Speed	Auto (2 mm/sec)
Date/Time	9/26/2021 1:09PM
Update	9/26/2021 1:10PM
Operator	IR
File Name	Memory#57
Sample Name	5b
Comment	

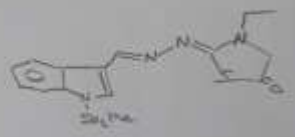

No.	cm-1	%T	No.	cm-1	%T	No.	cm-1	%T
1	3428.81	91.5879	2	3128.94	97.2015	3	2971.77	97.0571
4	2928.36	97.4275	5	2348.87	99.0291	6	1715.37	90.0849
7	1620.88	82.9574	8	1565.92	89.1693	9	1541.81	91.4016
10	1442.49	93.2873	11	1363.43	87.3801	12	1239.04	92.673
13	1165.76	85.9426	14	1122.37	85.5986	15	989.304	87.6304
16	890.952	93.2902	17	820.563	93.4561	18	771.387	88.2106
19	588.182	91.1644	20	542.863	85.7195	21	507.187	86.7964

Information Classification: General

RT: 3.81 - 3.94 SM: 7G

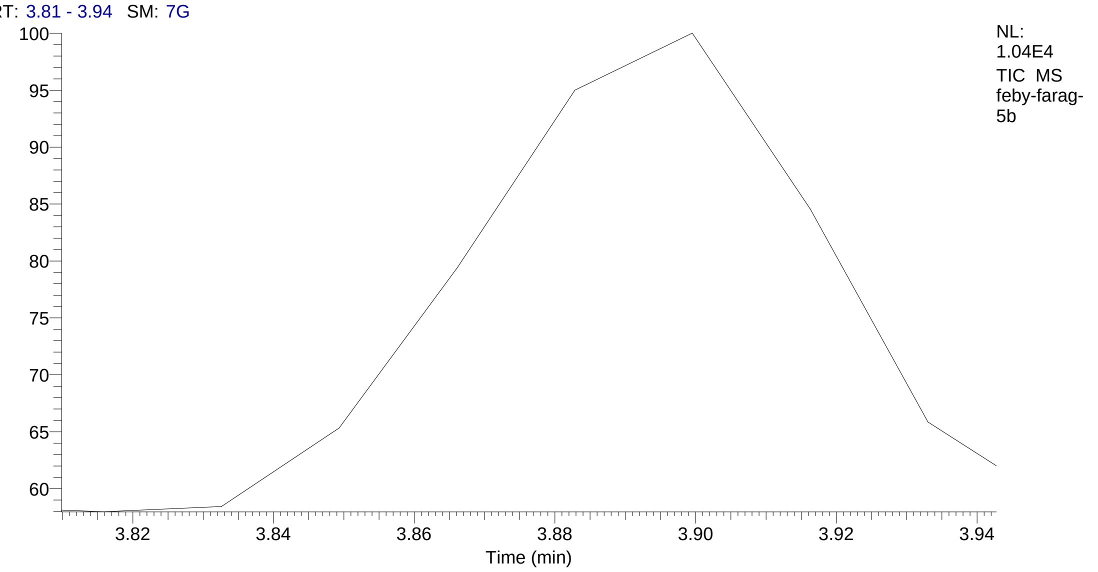

feby-farag-5b #207 RT: 3.48 AV: 1 NL: 4.28E2
T: + c EI Full ms [40.00-1000.00]

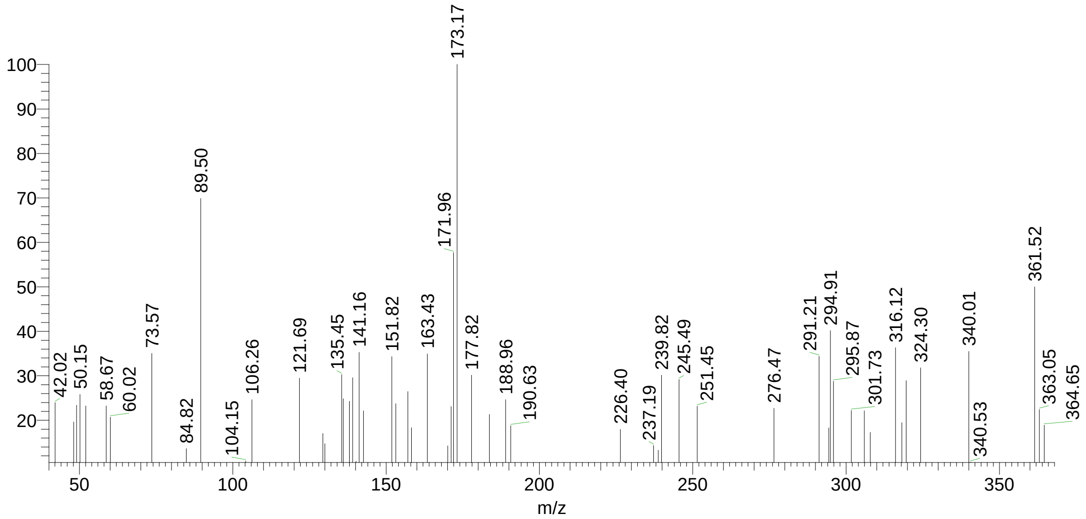

Information Classification: General

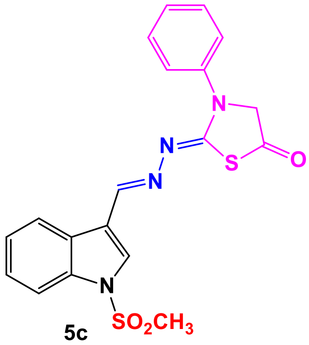

5c

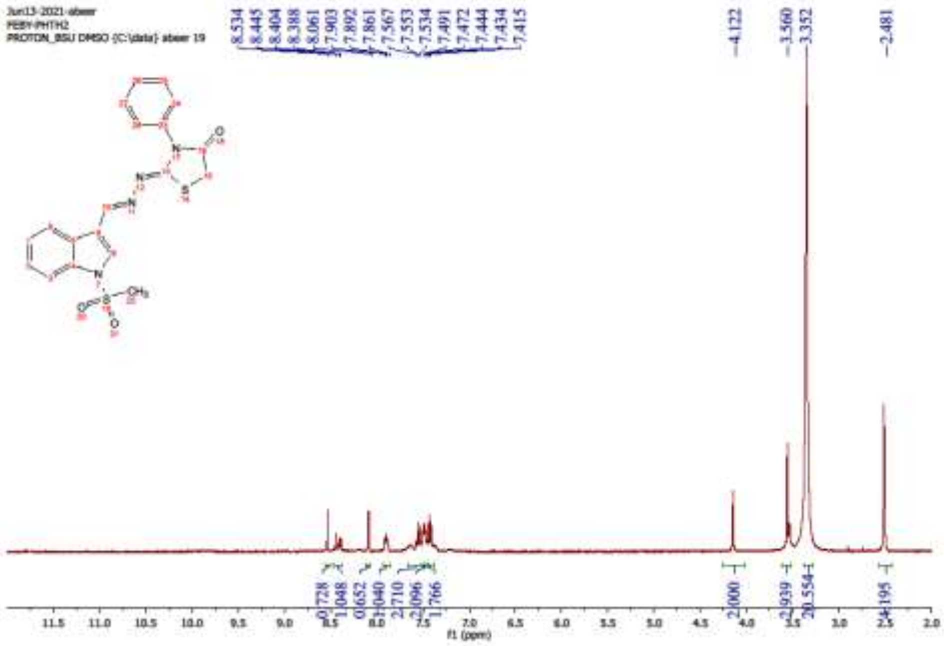

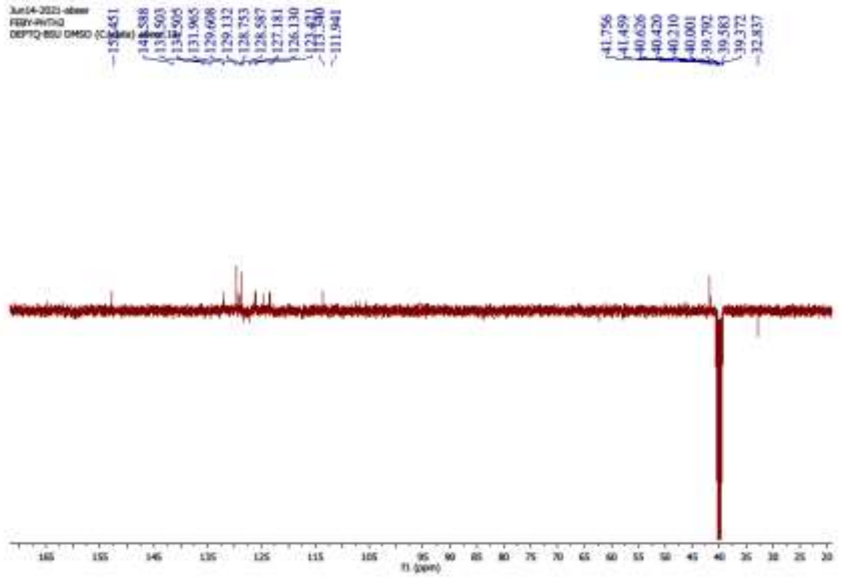

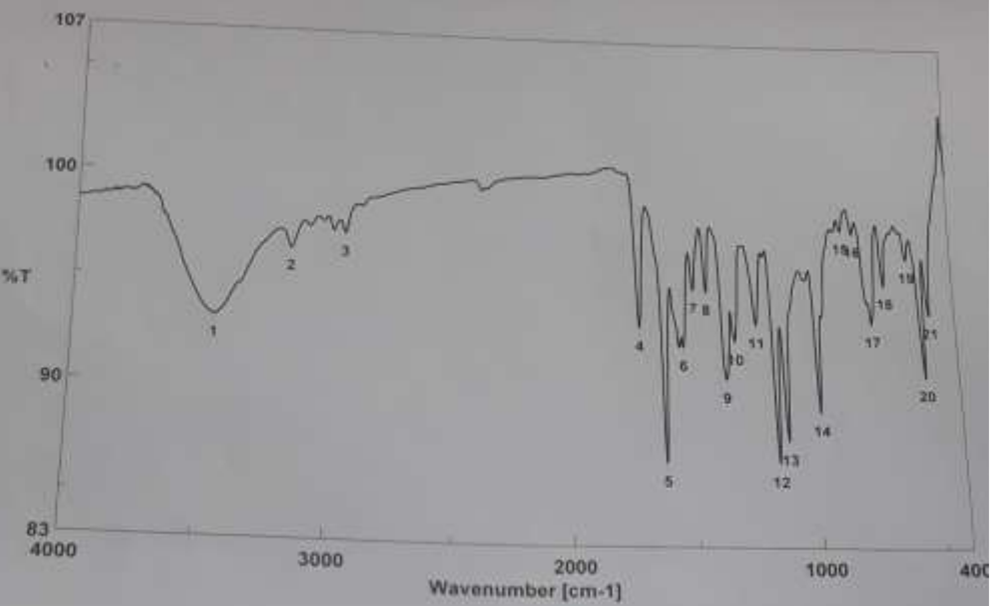

Accumulation	16
Resolution	4 cm-1
Zero Filling	ON
Apodization	Cosine
Gain	Auto (2)
Scanning Speed	Auto (2 mm/sec)
Date/Time	9/26/2021 1:07PM
Update	9/26/2021 1:07PM
Operator	IR
File Name	Memory#47
Sample Name	5c
Comment	

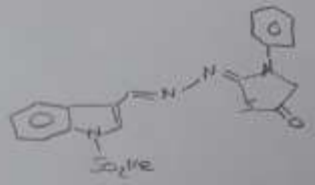

No.	cm-1	%T	No.	cm-1	%T	No.	cm-1	%T
1	3439.42	93.1052	2	3140.51	96.3554	3	2922.59	97.1
4	1722.12	93.0486	5	1619.91	86.8533	6	1543.74	92.1833
7	1497.45	94.9215	8	1444.42	94.8507	9	1369.21	90.7135
10	1330.64	92.5184	11	1241.93	93.3309	12	1164.79	86.8265
13	1124.3	87.652	14	988.339	89.2089	15	866.846	98.0309
16	817.67	97.9032	17	754.995	93.4861	18	692.32	95.3839
19	592.039	96.7196	20	541.899	90.9219	21	506.151	94.0093

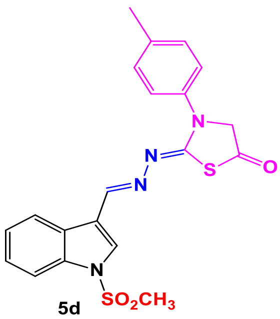

N
N
N
S
O
N
SO2CH3

5d

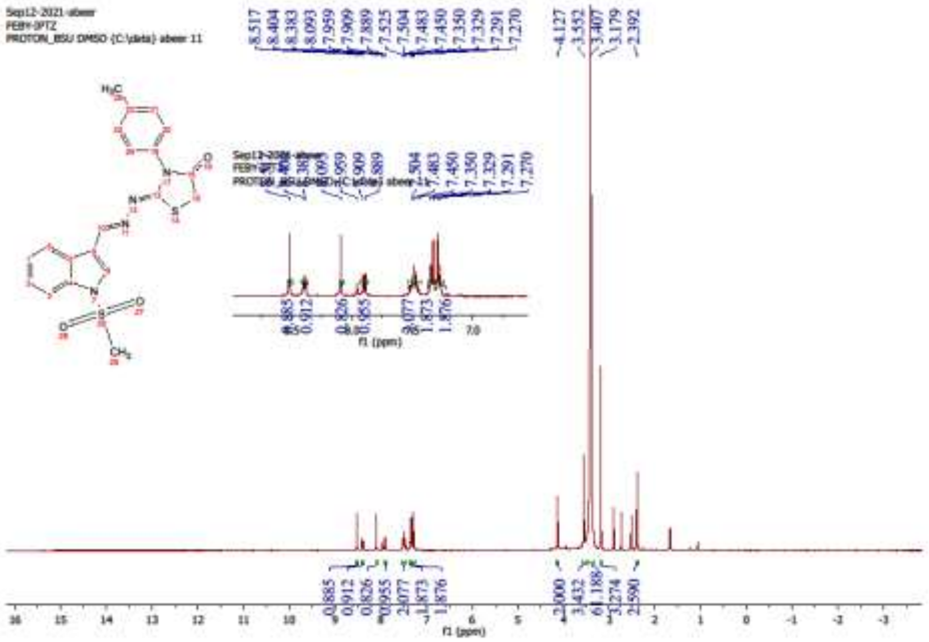

Sep12-2021-abeer
FEBY-IPTZ
PROTON_BSU DMSO {C:\data} abeer 11
8.517
8.404
8.383
8.093
7.999
7.909
7.889
7.525
7.504
7.483
7.450
7.350
7.329
7.291
7.270
4.127
3.552
3.407
3.179
2.392
0.885
0.912
0.826
0.955
2.077
1.873
1.876
2.000
3.432
61.188
3.274
2.590
f1 (ppm)

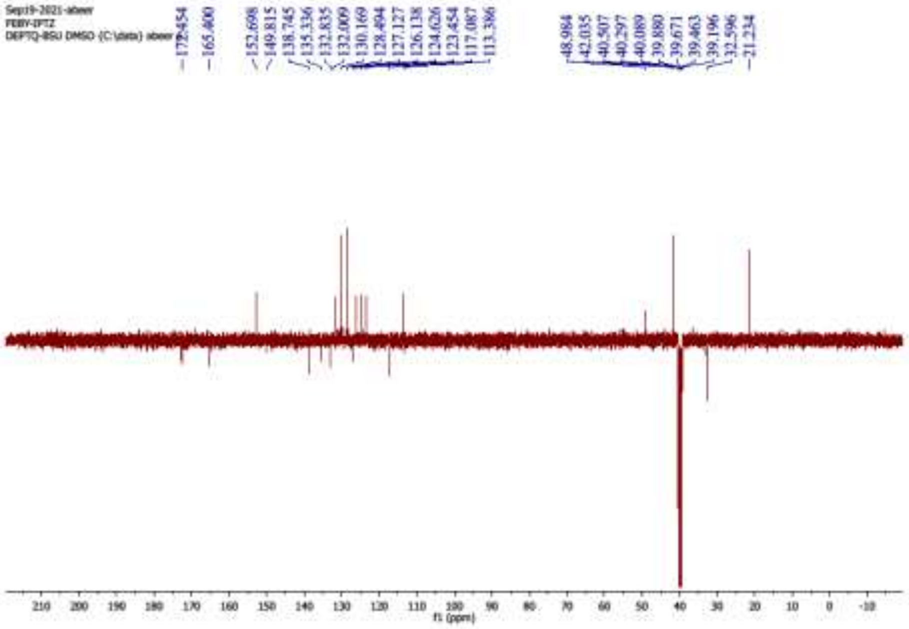

Sep19-2021-abeer
FEBY-IPTZ
DEPTQ-BSU DMSO {C:\data} abeer
172.454
165.400
152.698
149.815
138.745
135.336
132.835
132.009
130.169
128.494
127.127
126.138
124.626
123.454
117.087
113.386
48.984
42.035
40.507
40.297
40.089
39.880
39.671
39.463
39.196
32.596
21.234
f1 (ppm)

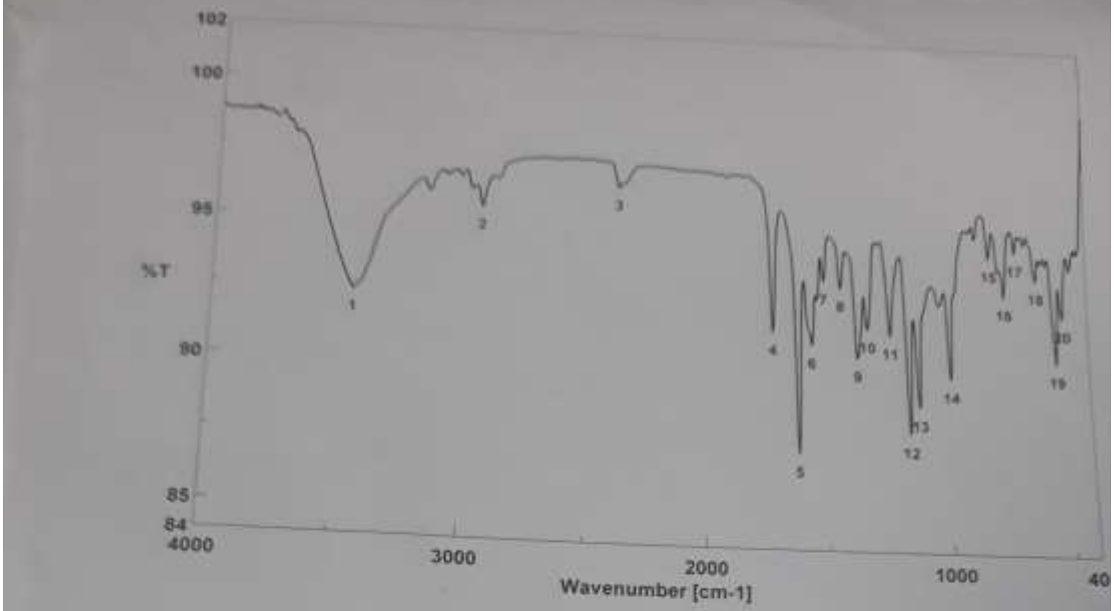

Accumulation	16
Resolution	4 cm-1
Zero Filling	ON
Apodization	Cosine
Gain	Auto (2)
Scanning Speed	Auto (2 mm/sec)
Date/Time	9/26/2021 1:18PM
Update	9/26/2021 1:18PM
Operator	IR
File Name	Memory#85
Sample Name	5d
Comment	

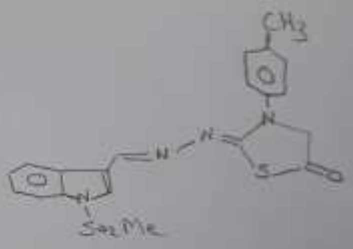

No.	cm-1	%T	No.	cm-1	%T	No.	cm-1	%T
1	3432.67	92.3869	2	2923.56	95.471	3	2359.48	96.3492
[illegible]	1725.01	91.4607	5	1620.88	87.2879	6	1564.95	91.0579
7	1513.85	93.3325	8	1444.42	93.0957	9	1376.93	90.6491
10	1334.5	91.6884	11	1240.97	91.4849	12	1163.83	88.0575
13	1124.3	89.0032	14	990.268	90.0306	15	815.742	94.4194
16	754.995	93.062	17	702.926	94.6605	18	617.109	93.6892
19	539.971	90.748	20	507.187	92.3885			

Information Classification: General

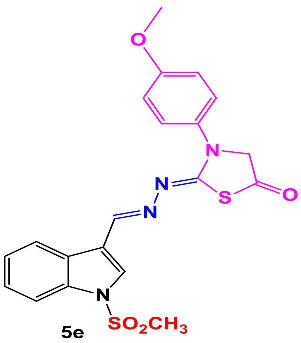

5e

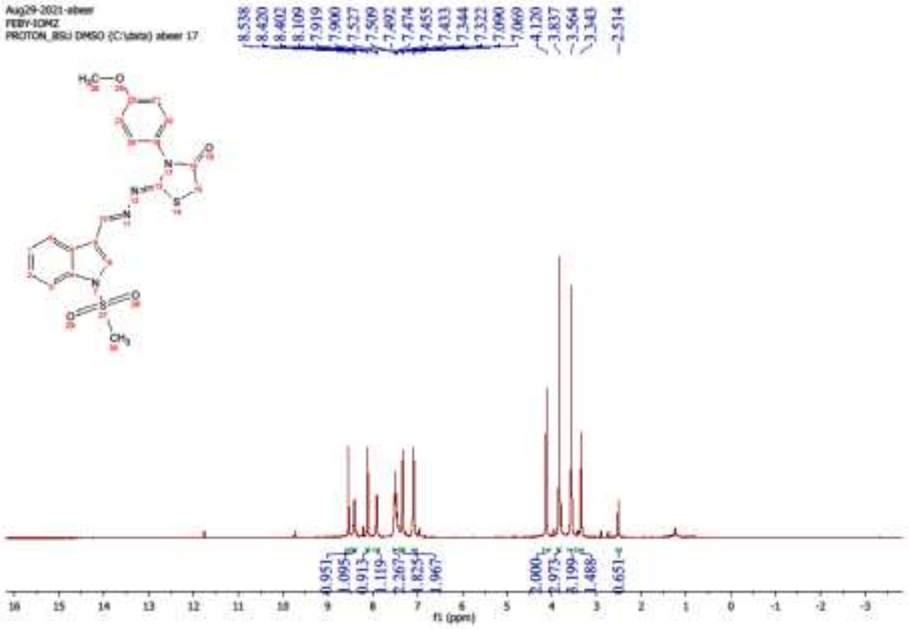

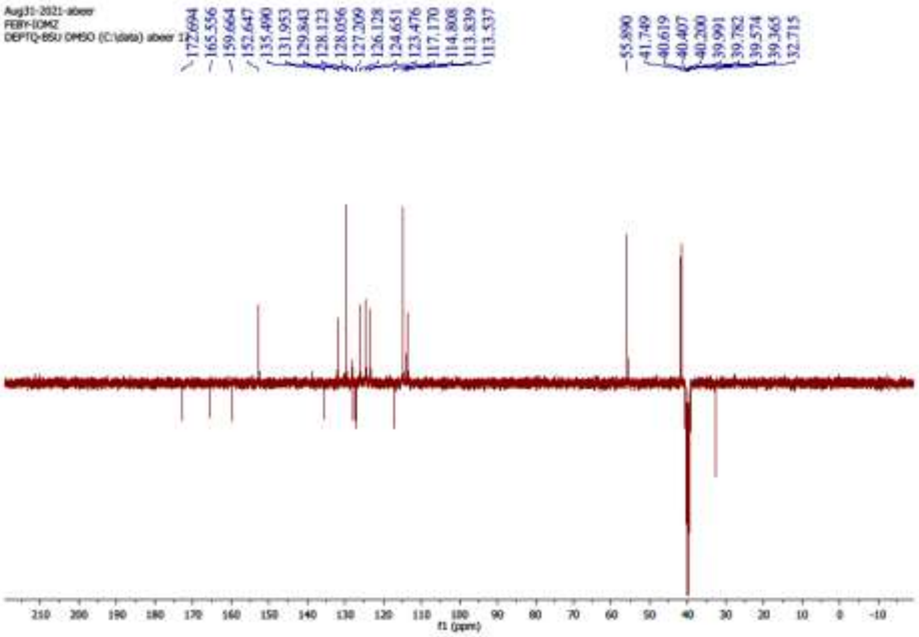

Information Classification: General

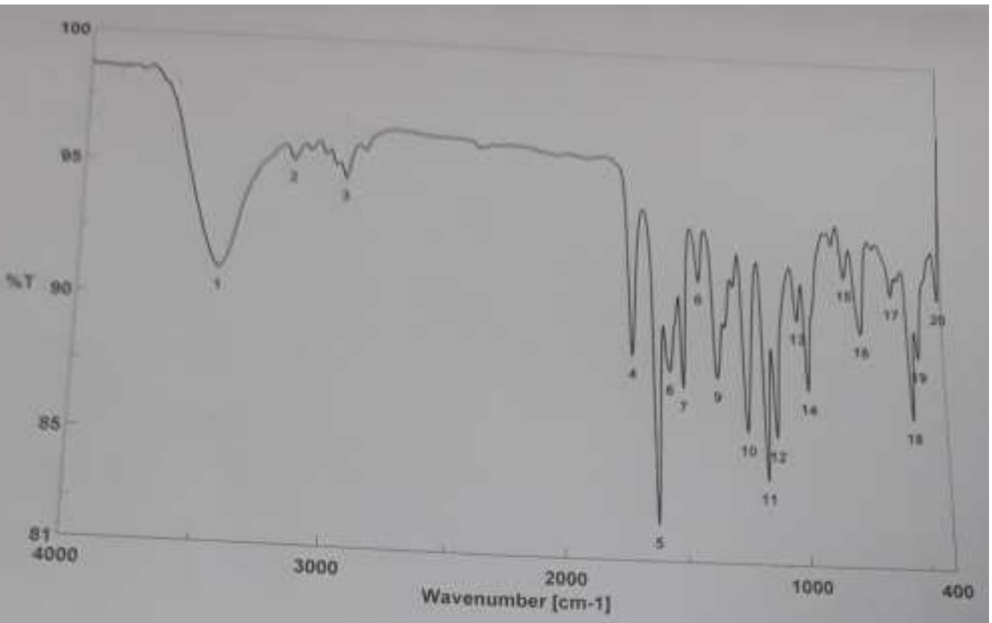

Accumulation	16
Resolution	4 cm-1
Zero Filling	ON
Apodization	Cosine
Gain	Auto (2)
Scanning Speed	Auto (2 mm/sec)
Date/Time	9/26/2021 1:03PM
Update	9/26/2021 1:04PM
Operator	IR
File Name	Memory#36
Sample Name	5e
Comment	

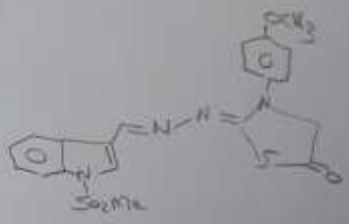

No.	cm-1	%T	No.	cm-1	%T	No.	cm-1	%T
1	3432.67	91.0319	2	3140.51	95.2372	3	2924.52	94.6068
4	1723.09	88.4405	5	1619.91	82.3445	6	1568.81	87.9047
7	1512.88	87.3229	8	1446.35	91.3524	9	1370.18	87.7259
10	1247.72	85.7882	11	1165.76	84.0527	12	1125.26	85.6204
13	1029.8	90.0345	14	988.339	87.4046	15	821.527	91.8058
16	755.959	89.6678	17	619.038	91.1926	18	542.863	86.5172
19	510.08	88.8472	20	416.549	91.0939			

Information Classification: General
