# Supplementary material for: Novel N-methylsulfonyl-indole derivatives: biological activity and COX-2/5-LOX inhibitory effect with improved gastro protective profile and reduced cardio vascular risks
Source: J Enzyme Inhib Med Chem. 2022 Dec 1;38(1):246–66. doi: 10.1080/14756366.2022.2145283 (PMC9721424; doi:10.1080/14756366.2022.2145283)
Supplement: Supplemental Material [file IENZ_A_2145283_SM4212.zip › IENZ_2145283_SuppMat2.pdf]

Researcher : Dr.Phoebe Farag email: Feby.Farag@yahoo.com mob. 01
Assay : COX1/2, 5LOX inhibitor screening
Samples : 12 compounds
Cell lines : ---
Ref. : ---
Date : 04-11-2021
Reader : Tecan Spark
Kit used : ---
Solvent : DMSO

Lab Report

ser	Compound		IC50 uM			SI
	code	MW	COX1	COX2	5-LOX	
1	3a		**21.14**±0.6	27.14±1.43	48.83±2.58	**0.779**
2	3b		**32.02**±0.91	22.01±1.16	14.81±0.78	**1.455**
3	4a		**11.34**±0.32	4.051±0.21	2.311±0.12	**2.799**
4	4b		**28.46**±0.8	86.15±4.55	29.17±1.54	**0.236**
5	4c		**5.01**±0.14	1.608±0.09	4.034±0.21	**3.116**
6	4d		**17.13**±0.49	7.212±0.38	17.92±0.95	**2.375**
7	4e		**24.71**±0.7	0.819±0.04	4.082±0.22	**30.17**
8	5a		**3.95**±0.11	6.667±0.35	9.23±0.49	**0.592**
9	5b		**22.2**±0.63	89.22±4.72	47.82±2.53	**0.249**
10	5c		**29.24**±0.83	8.06±0.43	79.43±4.2	**3.628**
11	5d		**5.785**±0.16	0.673±0.04	1.103±0.06	**8.596**
12	5e		**47.5**±1.35	4.884±0.26	6.646±0.35	**9.726**
*	**indomethacin**		**0.385**±0.01	---	---	
**	**Celecoxib**		**---**	0.463±0.02	---	
***	**Zileuton**		**---**	**---**	0.577±0.03	

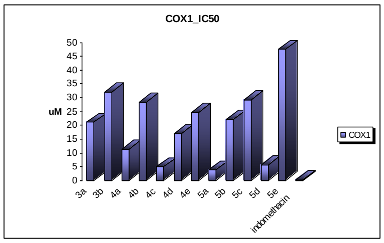

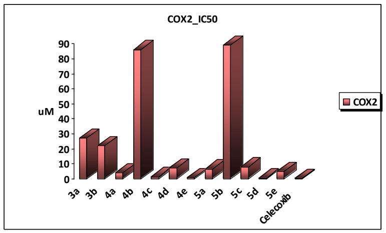

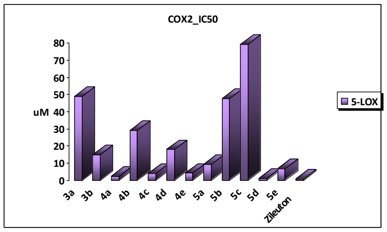

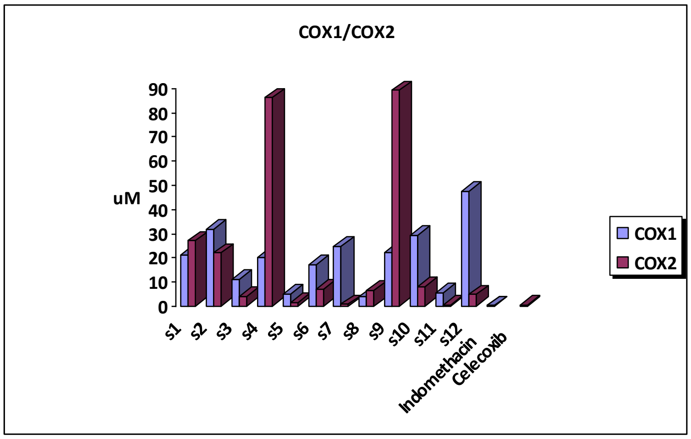

<u>Detailed results</u>

COX1

code	IC50	conc	log	%inh	T2	T1	ΔT	OD2	OD1	ΔOD	slope	K.Activity
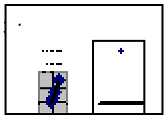		100	2	71	30	0	30	28.96	0	28.96	3.333	34.7555
		10	1	37.8	30	0	30	62.19	0	62.19	3.333	74.6355
		1	0	12.4	30	0	30	87.59	0	87.59	3.333	105.119
		0.1	-1	4.87	30	0	30	95.12	0	95.12	3.333	114.155
EC				0	30	0	30	100	0	100	3.3333	120
code	IC50	conc	log	%inh	T2	T1	ΔT	OD2	OD1	ΔOD	slope	K.Activity
3b		100	2	68.5	30	0	30	31.54	0	31.54	3.333	37.8518
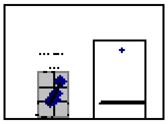		10	1	30.5	30	0	30	69.52	0	69.52	3.333	83.4323
		1	0	11.6	30	0	30	88.39	0	88.39	3.333	106.079
		0.1	-1	3.48	30	0	30	96.51	0	96.51	3.333	115.824
EC				0	30	0	30	100	0	100	3.3333	120
code	IC50	conc	log	%inh	T2	T1	ΔT	OD2	OD1	ΔOD	slope	K.Activity
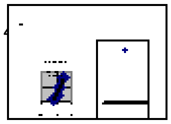		100	2	77.6	30	0	30	22.41	0	22.41	3.333	26.8947
		10	1	44	30	0	30	55.96	0	55.96	3.333	67.1587
		1	0	18.6	30	0	30	81.43	0	81.43	3.333	97.7258
		0.1	-1	7.54	30	0	30	92.45	0	92.45	3.333	110.951
EC				0	30	0	30	100	0	100	3.3333	120
code	IC50	conc	log	%inh	T2	T1	ΔT	OD2	OD1	ΔOD	slope	K.Activity
4b		100	2	73.3	30	0	30	26.71	0	26.71	3.333	32.0552
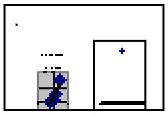		10	1	27.4	30	0	30	72.64	0	72.64	3.333	87.1767
		1	0	8.96	30	0	30	91.03	0	91.03	3.333	109.247
		0.1	-1	3.74	30	0	30	96.25	0	96.25	3.333	115.512
EC				0	30	0	30	100	0	100	3.3333	120
code	IC50	conc	log	%inh	T2	T1	ΔT	OD2	OD1	ΔOD	slope	K.Activity
4c		100	2	83.5	30	0	30	16.54	0	16.54	3.333	19.85
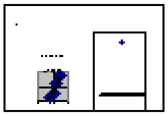		10	1	60.6	30	0	30	39.41	0	39.41	3.333	47.2967
		1	0	24.7	30	0	30	75.31	0	75.31	3.333	90.381
		0.1	-1	11	30	0	30	88.96	0	88.96	3.333	106.763
EC				0	30	0	30	100	0	100	3.3333	120
code	IC50	conc	log	%inh	T2	T1	ΔT	OD2	OD1	ΔOD	slope	K.Activity
4d		100	2	75.8	30	0	30	24.18	0	24.18	3.333	29.0189
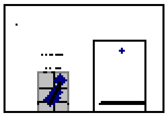		10	1	36.4	30	0	30	63.61	0	63.61	3.333	76.3396
		1	0	13.4	30	0	30	86.55	0	86.55	3.333	103.87
		0.1	-1	7.22	30	0	30	92.77	0	92.77	3.333	111.335
EC				0	30	0	30	100	0	100	3.3333	120

code	IC50	conc	log	%inh	T2	T1	ΔT	OD2	OD1	ΔOD	slope	K.Activity
4e		100	2	73.5	30	0	30	26.46	0	26.46	3.333	31.7552
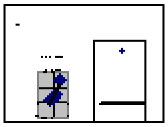		10	1	28.6	30	0	30	71.35	0	71.35	3.333	85.6286
		1	0	14.4	30	0	30	85.64	0	85.64	3.333	102.778
		0.1	-1	5.87	30	0	30	94.12	0	94.12	3.333	112.955
EC				0	30	0	30	100	0	100	3.3333	120
code	IC50	conc	log	%inh	T2	T1	ΔT	OD2	OD1	ΔOD	slope	K.Activity
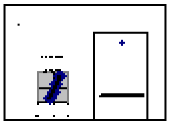		100	2	85.6	30	0	30	14.39	0	14.39	3.333	17.2697
		10	1	59	30	0	30	41.03	0	41.03	3.333	49.2409
		1	0	32.4	30	0	30	67.58	0	67.58	3.333	81.1041
		0.1	-1	13.6	30	0	30	86.35	0	86.35	3.333	103.63
EC				0	30	0	30	100	0	100	3.3333	120
code	IC50	conc	log	%inh	T2	T1	ΔT	OD2	OD1	ΔOD	slope	K.Activity
5b		100	2	73.6	30	0	30	26.38	0	26.38	3.333	31.6592
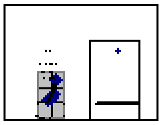		10	1	31.1	30	0	30	68.85	0	68.85	3.333	82.6283
		1	0	15.2	30	0	30	84.82	0	84.82	3.333	101.794
		0.1	-1	7.23	30	0	30	92.76	0	92.76	3.333	111.323
EC				0	30	0	30	100	0	100	3.3333	120
code	IC50	conc	log	%inh	T2	T1	ΔT	OD2	OD1	ΔOD	slope	K.Activity
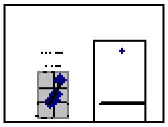		100	2	72.1	30	0	30	27.92	0	27.92	3.333	33.5074
		10	1	27.9	30	0	30	72.12	0	72.12	3.333	86.5527
		1	0	10.5	30	0	30	89.47	0	89.47	3.333	107.375
		0.1	-1	4.55	30	0	30	95.44	0	95.44	3.333	114.539
EC				0	30	0	30	100	0	100	3.3333	120
code	IC50	conc	log	%inh	T2	T1	ΔT	OD2	OD1	ΔOD	slope	K.Activity
5d		100	2	83.6	30	0	30	16.43	0	16.43	3.333	19.718
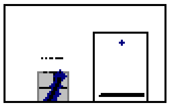		10	1	53.7	30	0	30	46.25	0	46.25	3.333	55.5056
		1	0	27.6	30	0	30	72.41	0	72.41	3.333	86.9007
		0.1	-1	8.83	30	0	30	91.16	0	91.16	3.333	109.403
EC				0	30	0	30	100	0	100	3.3333	120
code	IC50	conc	log	%inh	T2	T1	ΔT	OD2	OD1	ΔOD	slope	K.Activity
5e		100	2	68.5	30	0	30	31.52	0	31.52	3.333	37.8278
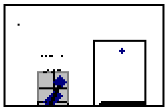		10	1	20	30	0	30	79.96	0	79.96	3.333	95.9616
		1	0	13.1	30	0	30	86.92	0	86.92	3.333	104.314
		0.1	-1	3.76	30	0	30	96.23	0	96.23	3.333	115.488
EC				0	30	0	30	100	0	100	3.3333	120
code	IC50	conc	log	%inh	T2	T1	ΔT	OD2	OD1	ΔOD	slope	K.Activity
Indomethacin		100	2	88.3	30	0	30	11.65	0	11.65	3.333	13.9814
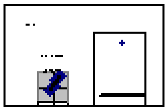		10	1	73.5	30	0	30	26.51	0	26.51	3.333	31.8152
		1	0	56.4	30	0	30	43.59	0	43.59	3.333	52.3132
		0.1	-1	40.5	30	0	30	59.47	0	59.47	3.333	71.3711
EC				0	30	0	30	100	0	100	3.3333	120

**3a**	y = 22.39x + 20.333
**3b**	y = 21.38x + 17.813
**4a**	y = 23.561x + 25.151
**4b**	y = 22.703x + 16.984
**4c**	y = 25.319x + 32.28
**4d**	y = 22.873x + 21.779
**4e**	y = 21.729x + 19.736
**5a**	y = 24.245x + 35.535
**5b**	y = 21.513x + 21.034
**5c**	y = 21.993x + 17.759
**5d**	y = 25.038x + 30.913
**5e**	y = 20.111x + 16.28
**Indomethacin**	y = 16.056x + 56.664

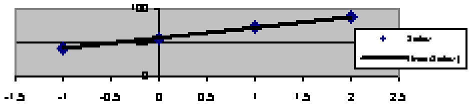

COX2

code	IC50	conc	log	%inh	T2	T1	ΔT	OD2	OD1	ΔOD	slope	K.Activity
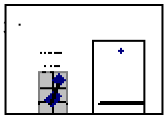		100	2	76	30	0	30	24.02	0	24.02	3.333	28.8269
		10	1	24.1	30	0	30	75.88	0	75.88	3.333	91.0651
		1	0	10.4	30	0	30	89.63	0	89.63	3.333	107.567
		0.1	-1	5.87	30	0	30	94.12	0	94.12	3.333	112.955
EC				0	30	0	30	100	0	100	3.3333	120
code	IC50	conc	log	%inh	T2	T1	ΔT	OD2	OD1	ΔOD	slope	K.Activity
3b		100	2	75.2	30	0	30	24.75	0	24.75	3.333	29.703
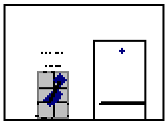		10	1	28.8	30	0	30	71.15	0	71.15	3.333	85.3885
		1	0	15.4	30	0	30	84.62	0	84.62	3.333	101.554
		0.1	-1	7.4	30	0	30	92.59	0	92.59	3.333	111.119
EC				0	30	0	30	100	0	100	3.3333	120
code	IC50	conc	log	%inh	T2	T1	ΔT	OD2	OD1	ΔOD	slope	K.Activity
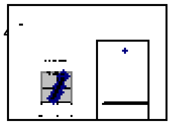		100	2	84.6	30	0	30	15.39	0	15.39	3.333	18.4698
		10	1	55.9	30	0	30	44.12	0	44.12	3.333	52.9493
		1	0	34.1	30	0	30	65.91	0	65.91	3.333	79.0999
		0.1	-1	15.7	30	0	30	84.33	0	84.33	3.333	101.206
EC				0	30	0	30	100	0	100	3.3333	120
code	IC50	conc	log	%inh	T2	T1	ΔT	OD2	OD1	ΔOD	slope	K.Activity
4b		100	2	61.5	30	0	30	38.51	0	38.51	3.333	46.2166
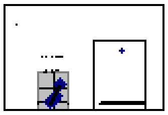		10	1	20.3	30	0	30	79.66	0	79.66	3.333	95.6016
		1	0	5.98	30	0	30	94.01	0	94.01	3.333	112.823
		0.1	-1	2.68	30	0	30	97.31	0	97.31	3.333	116.784
EC				0	30	0	30	100	0	100	3.3333	120
code	IC50	conc	log	%inh	T2	T1	ΔT	OD2	OD1	ΔOD	slope	K.Activity
4c		100	2	88.7	30	0	30	11.26	0	11.26	3.333	13.5134
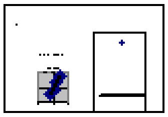		10	1	68.8	30	0	30	31.19	0	31.19	3.333	37.4317
		1	0	42.8	30	0	30	57.23	0	57.23	3.333	68.6829
		0.1	-1	25.2	30	0	30	74.84	0	74.84	3.333	89.817
EC				0	30	0	30	100	0	100	3.3333	120
code	IC50	conc	log	%inh	T2	T1	ΔT	OD2	OD1	ΔOD	slope	K.Activity
4d		100	2	81.4	30	0	30	18.61	0	18.61	3.333	22.3342
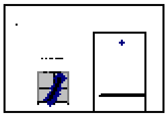		10	1	48.7	30	0	30	51.29	0	51.29	3.333	61.5542
		1	0	27	30	0	30	72.96	0	72.96	3.333	87.5608
		0.1	-1	8.42	30	0	30	91.57	0	91.57	3.333	109.895
EC				0	30	0	30	100	0	100	3.3333	120
code	IC50	conc	log	%inh	T2	T1	ΔT	OD2	OD1	ΔOD	slope	K.Activity
4e		100	2	90.4	30	0	30	9.55	0	9.55	3.333	11.4611

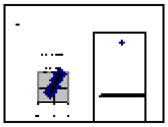		10	1	72.5	30	0	30	27.51	0	27.51	3.333	33.0153
		1	0	50.1	30	0	30	49.89	0	49.89	3.333	59.874
		0.1	-1	32.4	30	0	30	67.61	0	67.61	3.333	81.1401
EC				0	30	0	30	100	0	100	3.3333	120
code	IC50	conc	log	%inh	T2	T1	ΔT	OD2	OD1	ΔOD	slope	K.Activity
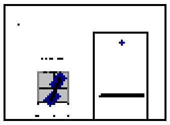		100	2	81.1	30	0	30	18.91	0	18.91	3.333	22.6943
		10	1	57.2	30	0	30	42.84	0	42.84	3.333	51.4131
		1	0	22.5	30	0	30	77.52	0	77.52	3.333	93.0333
		0.1	-1	5.34	30	0	30	94.65	0	94.65	3.333	113.591
EC				0	30	0	30	100	0	100	3.3333	120
code	IC50	conc	log	%inh	T2	T1	ΔT	OD2	OD1	ΔOD	slope	K.Activity
5b		100	2	66.4	30	0	30	33.56	0	33.56	3.333	40.276
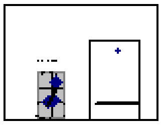		10	1	10.9	30	0	30	89.11	0	89.11	3.333	106.943
		1	0	5.87	30	0	30	94.12	0	94.12	3.333	112.955
		0.1	-1	2.34	30	0	30	97.65	0	97.65	3.333	117.192
EC				0	30	0	30	100	0	100	3.3333	120
code	IC50	conc	log	%inh	T2	T1	ΔT	OD2	OD1	ΔOD	slope	K.Activity
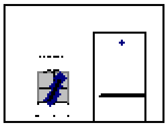		100	2	77.9	30	0	30	22.06	0	22.06	3.333	26.4746
		10	1	50.4	30	0	30	49.62	0	49.62	3.333	59.55
		1	0	26.1	30	0	30	73.86	0	73.86	3.333	88.6409
		0.1	-1	7.05	30	0	30	92.94	0	92.94	3.333	111.539
EC				0	30	0	30	100	0	100	3.3333	120
code	IC50	conc	log	%inh	T2	T1	ΔT	OD2	OD1	ΔOD	slope	K.Activity
5d		100	2	89	30	0	30	11.03	0	11.03	3.333	13.2373
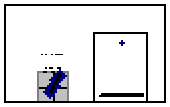		10	1	73	30	0	30	26.96	0	26.96	3.333	32.3552
		1	0	54.7	30	0	30	45.32	0	45.32	3.333	54.3894
		0.1	-1	33.2	30	0	30	66.79	0	66.79	3.333	80.156
EC				0	30	0	30	100	0	100	3.3333	120
code	IC50	conc	log	%inh	T2	T1	ΔT	OD2	OD1	ΔOD	slope	K.Activity
5e		100	2	83.6	30	0	30	16.42	0	16.42	3.333	19.706
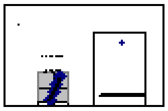		10	1	58.2	30	0	30	41.82	0	41.82	3.333	50.189
		1	0	30.9	30	0	30	69.14	0	69.14	3.333	82.9763
		0.1	-1	8.27	30	0	30	91.72	0	91.72	3.333	110.075
EC				0	30	0	30	100	0	100	3.3333	120
code	IC50	conc	log	%inh	T2	T1	ΔT	OD2	OD1	ΔOD	slope	K.Activity
Indomethacin		100	2	91.6	30	0	30	8.36	0	8.36	3.333	10.033
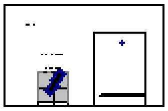		10	1	75.8	30	0	30	24.15	0	24.15	3.333	28.9829
		1	0	53.8	30	0	30	46.19	0	46.19	3.333	55.4335
		0.1	-1	38.9	30	0	30	61.12	0	61.12	3.333	73.3513
EC				0	30	0	30	100	0	100	3.3333	120

**3a**	y = 22.407x + 17.877
**3b**	y = 21.701x + 20.865
**4a**	y = 22.863x + 36.126
**4b**	y = 19.077x + 13.081
**4c**	y = 21.68x + 45.526
**4d**	y = 24.057x + 29.358
**4e**	y = 19.658x + 51.527
**5a**	y = 26.193x + 28.418
**5b**	y = 19.73x + 11.517
**5c**	y = 23.69x + 28.529
**5d**	y = 18.566x + 53.188
**5e**	y = 25.325x + 32.557
**Indomethacin**	y = 18.034x + 56.025

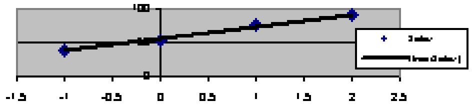

5-LOX

code	IC50	conc	log	%inh	T2	T1	ΔT	OD2	OD1	ΔOD	slope	K.Activity
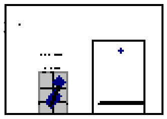		100	2	70.3	30	0	30	29.68	0	29.68	3.333	35.6196
		10	1	18.6	30	0	30	81.39	0	81.39	3.333	97.6778
		1	0	5.83	30	0	30	94.16	0	94.16	3.333	113.003
		0.1	-1	2.63	30	0	30	97.36	0	97.36	3.333	116.844
EC				0	30	0	30	100	0	100	3.3333	120
code	IC50	conc	log	%inh	T2	T1	ΔT	OD2	OD1	ΔOD	slope	K.Activity
3b		100	2	78.4	30	0	30	21.64	0	21.64	3.333	25.9706
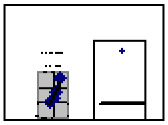		10	1	36.1	30	0	30	63.89	0	63.89	3.333	76.6757
		1	0	16.1	30	0	30	83.91	0	83.91	3.333	100.702
		0.1	-1	5.27	30	0	30	94.72	0	94.72	3.333	113.675
EC				0	30	0	30	100	0	100	3.3333	120
code	IC50	conc	log	%inh	T2	T1	ΔT	OD2	OD1	ΔOD	slope	K.Activity
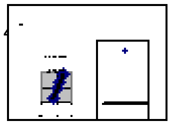		100	2	90.3	30	0	30	9.67	0	9.67	3.333	11.6052
		10	1	69	30	0	30	31.02	0	31.02	3.333	37.2277
		1	0	43.6	30	0	30	56.39	0	56.39	3.333	67.6748
		0.1	-1	11.4	30	0	30	88.61	0	88.61	3.333	106.343
EC				0	30	0	30	100	0	100	3.3333	120
code	IC50	conc	log	%inh	T2	T1	ΔT	OD2	OD1	ΔOD	slope	K.Activity
4b		100	2	70.1	30	0	30	29.86	0	29.86	3.333	35.8356
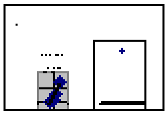		10	1	30.5	30	0	30	69.53	0	69.53	3.333	83.4443
		1	0	11.6	30	0	30	88.44	0	88.44	3.333	106.139
		0.1	-1	4.17	30	0	30	95.82	0	95.82	3.333	114.995
EC				0	30	0	30	100	0	100	3.3333	120
code	IC50	conc	log	%inh	T2	T1	ΔT	OD2	OD1	ΔOD	slope	K.Activity
4c		100	2	83.8	30	0	30	16.23	0	16.23	3.333	19.4779
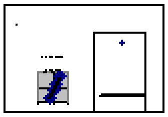		10	1	57.1	30	0	30	42.89	0	42.89	3.333	51.4731
		1	0	37.1	30	0	30	62.89	0	62.89	3.333	75.4755
		0.1	-1	12.1	30	0	30	87.91	0	87.91	3.333	105.503
EC				0	30	0	30	100	0	100	3.3333	120
code	IC50	conc	log	%inh	T2	T1	ΔT	OD2	OD1	ΔOD	slope	K.Activity
4d		100	2	77.2	30	0	30	22.82	0	22.82	3.333	27.3867
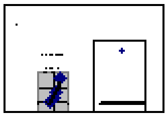		10	1	33.7	30	0	30	66.29	0	66.29	3.333	79.556
		1	0	12.5	30	0	30	87.49	0	87.49	3.333	104.998
		0.1	-1	4.88	30	0	30	95.11	0	95.11	3.333	114.143
EC				0	30	0	30	100	0	100	3.3333	120
code	IC50	conc	log	%inh	T2	T1	ΔT	OD2	OD1	ΔOD	slope	K.Activity
4e		100	2	85	30	0	30	15.02	0	15.02	3.333	18.0258

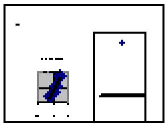		10	1	55.7	30	0	30	44.29	0	44.29	3.333	53.1533
		1	0	30.9	30	0	30	69.08	0	69.08	3.333	82.9043
		0.1	-1	17.9	30	0	30	82.11	0	82.11	3.333	98.5419
EC				0	30	0	30	100	0	100	3.3333	120
code	IC50	conc	log	%inh	T2	T1	ΔT	OD2	OD1	ΔOD	slope	K.Activity
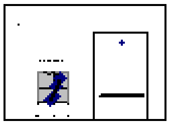		100	2	79	30	0	30	21.03	0	21.03	3.333	25.2385
		10	1	50.3	30	0	30	49.71	0	49.71	3.333	59.658
		1	0	17.4	30	0	30	82.64	0	82.64	3.333	99.1779
		0.1	-1	7.21	30	0	30	92.78	0	92.78	3.333	111.347
EC				0	30	0	30	100	0	100	3.3333	120
code	IC50	conc	log	%inh	T2	T1	ΔT	OD2	OD1	ΔOD	slope	K.Activity
5b		100	2	73.1	30	0	30	26.91	0	26.91	3.333	32.2952
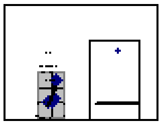		10	1	15.1	30	0	30	84.92	0	84.92	3.333	101.914
		1	0	3.84	30	0	30	96.15	0	96.15	3.333	115.392
		0.1	-1	1.82	30	0	30	98.17	0	98.17	3.333	117.816
EC				0	30	0	30	100	0	100	3.3333	120
code	IC50	conc	log	%inh	T2	T1	ΔT	OD2	OD1	ΔOD	slope	K.Activity
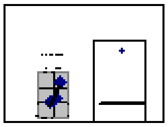		100	2	66.7	30	0	30	33.29	0	33.29	3.333	39.952
		10	1	13	30	0	30	87.02	0	87.02	3.333	104.434
		1	0	5.84	30	0	30	94.15	0	94.15	3.333	112.991
		0.1	-1	2.32	30	0	30	97.67	0	97.67	3.333	117.216
EC				0	30	0	30	100	0	100	3.3333	120
code	IC50	conc	log	%inh	T2	T1	ΔT	OD2	OD1	ΔOD	slope	K.Activity
5d		100	2	86.9	30	0	30	13.08	0	13.08	3.333	15.6976
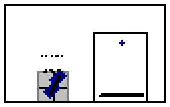		10	1	70.6	30	0	30	29.42	0	29.42	3.333	35.3075
		1	0	47.6	30	0	30	52.43	0	52.43	3.333	62.9223
		0.1	-1	30.3	30	0	30	69.74	0	69.74	3.333	83.6964
EC				0	30	0	30	100	0	100	3.3333	120
code	IC50	conc	log	%inh	T2	T1	ΔT	OD2	OD1	ΔOD	slope	K.Activity
5e		100	2	80.8	30	0	30	19.17	0	19.17	3.333	23.0063
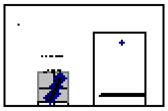		10	1	53	30	0	30	47.02	0	47.02	3.333	56.4296
		1	0	24.1	30	0	30	75.92	0	75.92	3.333	91.1131
		0.1	-1	11.6	30	0	30	88.39	0	88.39	3.333	106.079
EC				0	30	0	30	100	0	100	3.3333	120
code	IC50	conc	log	%inh	T2	T1	ΔT	OD2	OD1	ΔOD	slope	K.Activity
**Zileuton**		100	2	92.8	30	0	30	7.22	0	7.22	3.333	8.66487
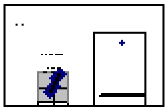		10	1	77.1	30	0	30	22.91	0	22.91	3.333	27.4947
		1	0	56.5	30	0	30	43.51	0	43.51	3.333	52.2172
		0.1	-1	32.9	30	0	30	67.12	0	67.12	3.333	80.5521
EC				0	30	0	30	100	0	100	3.3333	120

**3a**	y = 21.583x + 13.553
**3b**	y = 23.928x + 21.989
**4a**	y = 26.222x + 40.462
**4b**	y = 21.681x + 18.24
